# How to Make Epidemiological Training Infectious

**DOI:** 10.1371/journal.pbio.1001295

**Published:** 2012-04-03

**Authors:** Steve E. Bellan, Juliet R. C. Pulliam, James C. Scott, Jonathan Dushoff, the MMED Organizing Committee

**Affiliations:** 1 Department of Environmental Science, Policy & Management, University of California, Berkeley, California, United States of America; 2 Fogarty International Center, National Institutes of Health, Bethesda, Maryland, United States of America; 3 Department of Biology and Emerging Pathogens Institute, University of Florida, Gainesville, Florida, United States of America; 4 Department of Mathematics and Statistics, Colby College, Waterville, Maine, United States of America; 5 Department of Biology, McMaster University, Hamilton, Ontario, Canada; University of California Berkeley/Joint Genome Institute, United States of America

## Abstract

In this fun, interactive exercise, students simulate an infectious disease outbreak among themselves that conceptually integrates two historically distinct fields in epidemiology.

**Summary**

Modern infectious disease epidemiology builds on two independently developed fields: classical epidemiology and dynamical epidemiology. Over the past decade, integration of the two fields has increased in research practice, but training options within the fields remain distinct with few opportunities for integration in the classroom. The annual Clinic on the Meaningful Modeling of Epidemiological Data (MMED) at the African Institute for Mathematical Sciences has begun to address this gap. MMED offers participants exposure to a broad range of concepts and techniques from both epidemiological traditions. During MMED 2010 we developed a pedagogical approach that bridges the traditional distinction between classical and dynamical epidemiology and can be used at multiple educational levels, from high school to graduate level courses. The approach is hands-on, consisting of a real-time simulation of a stochastic outbreak in course participants, including realistic data reporting, followed by a variety of mathematical and statistical analyses, stemming from both epidemiological traditions. During the exercise, dynamical epidemiologists developed empirical skills such as study design and learned concepts of bias while classical epidemiologists were trained in systems thinking and began to understand epidemics as dynamic nonlinear processes. We believe this type of integrated educational tool will prove extremely valuable in the training of future infectious disease epidemiologists. We also believe that such interdisciplinary training will be critical for local capacity building in analytical epidemiology as Africa continues to produce new cohorts of well-trained mathematicians, statisticians, and scientists. And because the lessons draw on skills and concepts from many fields in biology—from pathogen biology, evolutionary dynamics of host–pathogen interactions, and the ecology of infectious disease to bioinformatics, computational biology, and statistics—this exercise can be incorporated into a broad array of life sciences courses.

## Introduction

The goal of epidemiology is to identify the biological, behavioral, and environmental causes of health outcomes or diseases and apply this knowledge to the development of effective disease interventions to improve public health [1]–[3]. Diseases are complex phenomena that arise from various interacting processes, challenging epidemiologists to extract important causal relationships from observational and experimental data [1]. Infectious diseases add a level of complexity because infection occurs through (direct or indirect) interaction between susceptible and infectious individuals. The rate of new infections depends on the number or proportion of individuals who are susceptible and infectious. These quantities themselves evolve through time as new individuals become infected. In other words, infectious disease dynamics are nonlinear. The biological, behavioral, evolutionary, and environmental nuances of each disease–host system determine the dynamics of disease incidence and have important implications for designing and evaluating interventions. Patterns of disease do not only arise from individual-level characteristics (e.g., genetics, behavior, age, sex, health status), but also from the history of infection in the greater population. Thus, consideration of individual-level data independent of the population context may be misleading [4],[5]. In fact, the number or proportion of susceptible, infected, and immune individuals in the population often plays a greater role in determining the current individual-level risk of infection than an individual's characteristics [6],[7].

Thus, population-level epidemiological patterns emerge from the complex, interacting processes governing pathogen biology, host biology, host behavior, the environment, and their interactions [4],[8]. The field of epidemiology has taken two broad methodological approaches to understand these interacting processes. While various terms have been used to describe these two subfields, we call them classical epidemiology and dynamical epidemiology (see Text S1 for more details). Classical epidemiology builds on assumptions of independence between individuals (or clusters thereof) to identify correlations between risk (or protective) factors and disease, with the goal of establishing a causal relationship between them [1],[3],[9]. Dynamical (mechanistic) epidemiology has largely developed from ecology and its subfield, population dynamics, along with other applications of mathematics to biological systems [10],[11]. In contrast to the correlational approach of classical epidemiology, dynamical epidemiology aims to understand disease dynamics with nonlinear transmission models that explicitly account for the interactions between individuals [5],[8],[12].

Firmly rooted in empirical research, classical epidemiology takes a phenomenalistic approach that focuses more on whether a causal relationship exists [13] than the mechanistic nature of the relationship [5]. While extremely practical, this reductionist approach frequently ignores the dependence between observations of infectious outcomes and can lead to spurious conclusions [5],[14],[15]. Dynamical epidemiology, in contrast, focuses on how mechanistic interactions between individuals lead to population-scale patterns and emphasizes understanding realistic dynamics. Ironically, this realism often comes at the expense of being partially or completely divorced from real data, because detailed data on mechanistic processes are rare. Further, dynamic models frequently ignore sources of bias or random error in the data, which can dramatically alter model results [6],[16]. For example, dynamic models often use parameters from the literature to describe mechanistic processes without careful attention to the original source of the parameter's estimation and associated bias or error therein. Model results are also often used to claim strong conclusions without comparing model outputs to real epidemiological time series data. Dynamic epidemiologists nevertheless attempt to make robust conclusions using sensitivity analyses that explore model behavior over a large set of viable hypothetical scenarios.

Both approaches have played successful roles in public health [8],[17]–[19], though remaining rather distinct fields with studies using dynamical models often published in ecological, mathematical, or interdisciplinary journals but rarely in epidemiological journals. Fifteen years ago Susser and Susser (1996) advocated the more coherent discipline of eco-epidemiology, with classical epidemiology subsumed by a greater ecological framework that considers mechanistic processes at various physical, temporal, and demographic scales [3],[20],[21]. And though, in our experience, scientists at advanced stages in either field are often largely unaware of the other field's existence, projects integrating these approaches have increased in research practice, often yielding otherwise unattainable insight. For example, using a dynamic model Lietman et al. (1999) found that trachoma (the leading cause of infectious blindness worldwide) elimination may be feasible by biannual treatment of children with a single dose of azithromycin [22]. They then formulated a cluster-randomized controlled trial that verified this finding empirically [23]. Finally, after fitting a dynamic model to the empirical data collected during this trial they demonstrated that individual-level transmission efficiency decreases with decreasing disease prevalence, making elimination of the disease easier than expected [24]. As a second example, Eisenberg et al. (2011) found that individual-level antibiotic use proved insufficient to explain geographic variation in the prevalence of *Escherichia coli* antibiotic resistance, but that this phenomenon could be explained by interactions between village-specific antibiotic use and transmission rates in a dynamic model [15]. In both examples, a close alliance between methods in classical and dynamical epidemiology allowed systems mechanisms to inform study design and vice versa, yielding insight with a real potential to improve public health.

Despite such successes, training options within the fields remain distinct. Furthermore, the very few scientists with training in both disciplines seldom receive formal instruction explaining how they complement each other. Reflecting the severity of this rift, a recent published symposium on the future of epidemiological education lacked any discussion of dynamical approaches [25]–[28]. We do not believe that all dynamical epidemiologists must be able to design empirical studies nor that all classical epidemiologists must be able to construct dynamical models. However, understanding the utility of and fundamental concepts within each discipline will allow fruitful collaboration between the fields as demonstrated in the examples above. The ability to think from both perspectives allows researchers to choose the most suitable set of methods for a given question [1],[3],[9],[20],[21].

The annual Clinic on the Meaningful Modeling of Epidemiological Data (MMED) at the African Institute for Mathematical Sciences in Muizenberg, South Africa, is working to address this gap. African capacity in epidemiology is in short supply despite the continent's disproportionate burden of disease and burgeoning new cohorts of African students trained in biomathematics, biology, and public health. MMED offers participants (ranging from undergraduate students to professors) exposure to a broad range of concepts and techniques from both classical and dynamical epidemiology, while explicitly highlighting how the two approaches complement each other and fit into a larger context. Selected lecture material from MMED is provided in the Figures S1, S2, S3, S4, S5, S6, S7, S8.

During MMED we designed the following pedagogical tool to address the rift between classical and dynamical epidemiology in which participants not only design studies and collect data prior to analysis, but also observe pathogen transmission in a way that reveals how epidemics are inherently nonlinear stochastic (i.e., random) processes. The approach is hands-on, consisting of the real-time simulation of a stochastic outbreak among course participants, including realistic data reporting, followed by analyses from both epidemiological traditions. We believe this type of integrated educational tool can stimulate the training of a cohort of infectious disease epidemiologists who are well acquainted with both the dynamical systems nature of epidemiological processes as well as the empirical design and analytical issues associated with investigating causal relationships between risk factors and disease.

## The Emergence of an Infectious Disease: Muizenberg Mathematical Fever

At the 2010 and 2011 MMED Clinics we instigated outbreaks of a novel infectious agent, Muizenberg Mathematical Fever (MMF), in our course participants. The infectious agent was a paper form (Text S2). Index cases were initiated through surreptitious distribution of a small number of infection forms on the first day of the Clinic. The paper provided simple instructions for newly infected individuals. Following the instructions, individuals used random number generators in the free statistical programming language R [29] to determine a Poisson random number (see Box 1) of potentially infectious contacts they would generate. The infected individual would then print out new infection forms from a webpage and discreetly hand these to other participants without knowledge of who had already been infected, recording the time of those infectious contacts. A Bernoulli random number generator was similarly used to determine whether or not an infection was symptomatic. Only symptomatic individuals would report to our health care system (a specified faculty member) that they were sick. However, all new infections were reported to a second faculty member via email for tracking purposes. Recovery consisted of delivering the infecting paper to a third faculty member. Thus, each outbreak generated two datasets: (1) a realistic provider-based surveillance system that recorded symptomatic cases and time of symptom onset; and (2) an unrealistically accurate knowledge of the underlying transmission dynamics.

**Box 1. Teaching Tools**

**Prior to adopting this exercise, we recommend reviewing Datasets S1–S7, Figures S1–S8, Texts S1–S5, and linked resources. These include:**

- MMED lecture slides (Figures S1–S8)
- An example document that provides instructions that students are to carry out upon being exposed (this document doubles as the infectious agent; being handed this document constitutes exposure to MMF)
- Data obtained (by the authors) from prior MMF outbreaks
- An example survey used to identify risk factors associated with contracting MMF and corresponding data.
- R code that organizers may experiment with to assist them in determining initial parameter values (for more information on the R computing language and to download the latest version of R, please see http://www.r-project.org)
- Additional R code that provides illustrative exercises that can be performed using MMF data (e.g., identification of risk factors, calculating measures of effect, confidence intervals, etc.)
- Additional online resources containing introductory materials on both epidemiological traditions
- Information on infectious diseases and epidemics to share with students (e.g., ProMED, Center for Disease Control website, and other such resources)

**Preparation guide**

- Before implementation, organizers should set values for epidemiological parameters (initial number of infections, potential protective factors, *R*_0_, the proportion symptomatic) that take into account the number of participants and the types of analyses that students will perform; we suggest doing this by simulating outbreak dynamics and have provided example R code Texts S3–S4.
- If a protective factor is induced (e.g., “vaccination” or other form of immunity), ensure that the number of immune individuals will be sufficient for an effect to be detected and that the epidemic is likely to take off when accounting for immunity; also, attempt to make the factor something that will be readily detectable via an appropriately designed questionnaire
- Prepare a full roster of participating individuals (i.e., those that could be exposed) for tracking purposes
- Provide some brief training for individuals so that they are able to generate the appropriate random numbers (note: while we have had success with R, any software package that can generate Poisson and Bernoulli random variables could be used)

**Tips for running the exercise as part of a course for credit**

- To ensure timely and full participation, we suggest using incentives (e.g., linking participation to student evaluation, course participation credit, etc.).
- Do not initiate the outbreak until the course list has been finalized (i.e., after the end of the drop/add period)
- If possible, provide a link to the infectious agent (i.e., the instruction document) that is only accessible to course participants (e.g., through a Blackboard or Sakai site) and cannot be found by searching; this will help ensure that the epidemic is confined to the closed population of course participants
- Make sure participants know whom they may and may not infect (e.g., provide a course roster to each student or refer them to a course management website).
- Once the outbreak is underway, discretely remind participants to follow through accordingly (i.e., “I was expecting to receive emails from some of you—don't forget to follow up”)

The outbreaks percolated through participants before burning out, much like epidemics of biological pathogens. While we determined *R*_0_ (the number of susceptible individuals infected by an index case introduced into an entirely susceptible population; see glossary in Text S1) and the symptomatic proportion, participants' behavior (e.g., who they infected and when they did it) also affected disease dynamics. The unpredictable nature of these events added to the realism of our outbreak. During the 2011 outbreak, we dictated that repeat attendees would be immune to MMF, thereby seeding a protective (risk) factor for participants to find during the analytical stage of the exercise.

## Outbreak Investigation

**Figure 1:**
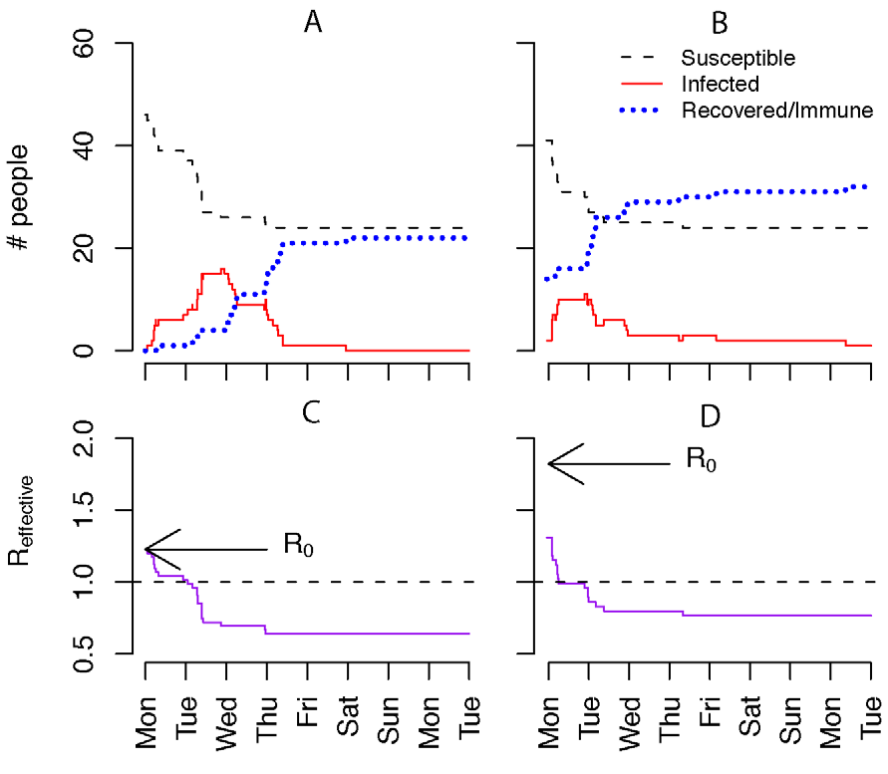
Muizenberg Mathematical Fever epidemic time series. Epidemic time series for the outbreaks at the 2010 (A) and 2011 (B) outbreaks. The former and latter outbreaks differ by their different basic reproductive numbers (defined as the average number of people an infectious individual infects if the rest of the population is susceptible; *R*_0_ = 1.23 and 1.82, respectively), the initial number of infectious individuals in the population (2 and 4, respectively), and the number of individuals immune at the start of the outbreak (0 and 14, respectively). (C and D) demonstrate how the effective reproductive number (*R*_eff_; average number of individuals each infected person infects) changes during the course of the outbreak as the number of susceptibles decreases and that the epidemic begins to burn out when *R*_eff_ decreases below 1 and infectious individuals no longer replace themselves with new infections. The script for production of and further detail on this figure are given in Text S3.

**Figure 2 pbio-1001295-g002:**
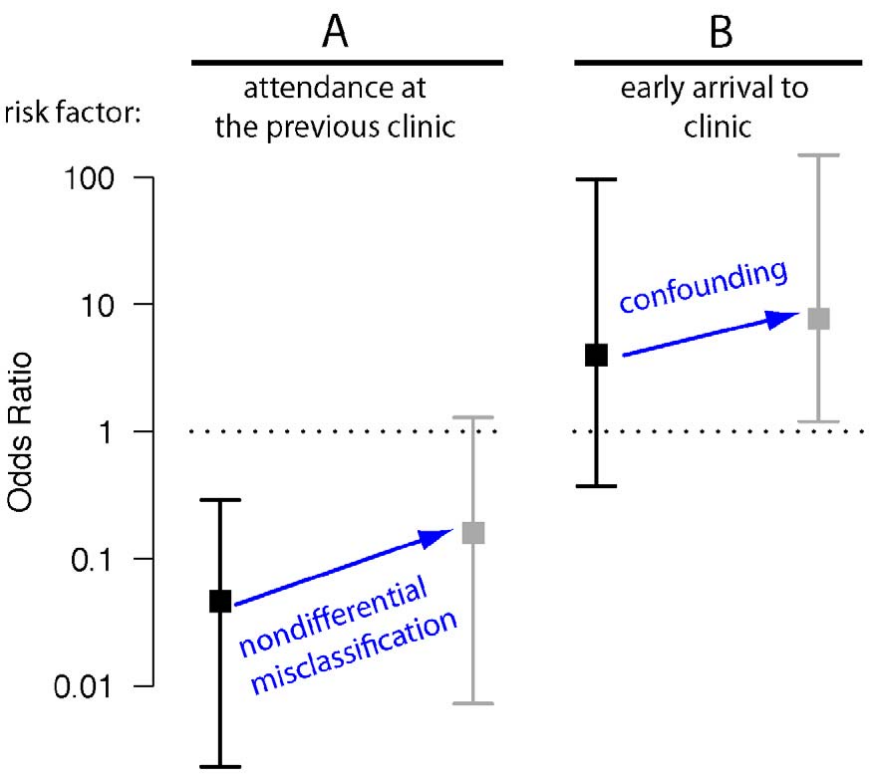
Demonstration of information and confounding bias with simulated outbreak data. (A) Muizenberg Mathematical Fever 2011 outbreak data to illustrate how using a case definition with imperfect sensitivity (symptomatic disease) can cause nondifferential misclassification bias (the category of information bias where exposed and unexposed individuals are equally likely to be misclassified). Nondifferential misclassification biases the association between a risk factor and a disease outcome towards the null hypothesis of no association (odds ratio = 1). While attendance at the prior year's clinic was actually protective (black square and 95% CI), this bias was sufficient to cause the confidence interval for the odds ratio of this very protective variable to overlap (gray). (B) illustrates how a risk factor (arrival a day or more early to the clinic) that has no real association to a disease outcome can appear associated through confounding. Individuals who had attended the clinic in prior years were less likely to come to the clinic early and were also protected (i.e., A). Consequently, early attendance appeared associated with a higher risk of disease in a univariate analysis (gray) though the CI contains the null hypothesis of no association in a multivariate analysis that adjusts for prior attendance (black). The script for production of and further detail on this figure are given in Text S3.

**Figure 3:**
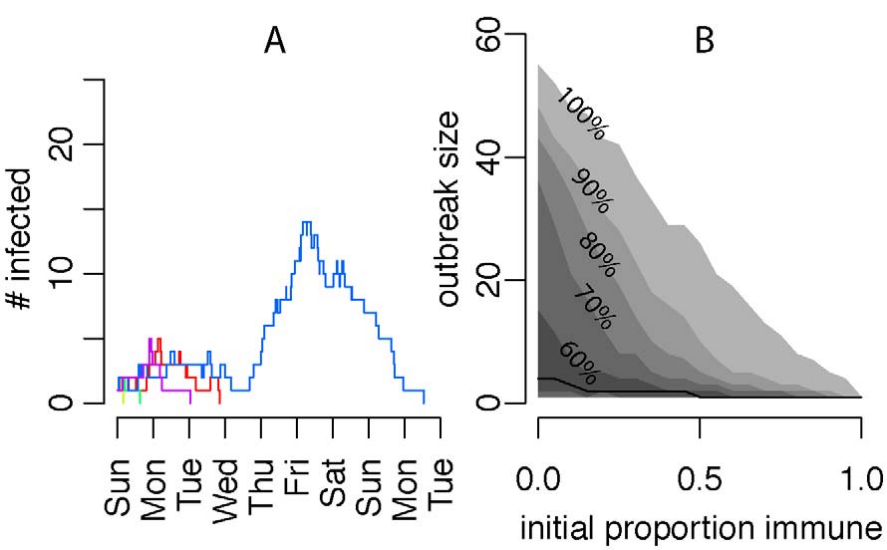
Utility of stochastic simulation to study the effect of vaccination on future outbreaks. (A) Five stochastic simulations of Muizenberg Mathematical Fever outbreaks using transmission parameters fit from the 2011 outbreak data but with only one infected individual initiating the outbreak (instead of four) but the same proportion initially immune (25%). In comparison to Figure 1A, the outbreak would appear less likely to take off (with four simulated outbreaks burning out by Wednesday) if only one infected individual initiated the outbreak. (B) shows the distribution of outbreak size (total number infected before burnout) among simulated outbreaks in populations with different immune proportions at the outbreak initiation. The black line shows the median outbreak size among 1,000 stochastic simulations for each immune proportion and the gray regions show 10% quantiles of outbreak size. Only small levels of immunity are necessary to make outbreaks rare (with a fifth of the population immune 50% of simulated outbreaks never exceed more than two secondary cases), but just by chance larger outbreaks still occur. The script for production of and further detail on this figure are given in Text S3.

Initiation of the MMF outbreaks resulted in a complex epidemiological process (Figure 1) that produced several datasets. These data gave participants the opportunity to apply a variety of methods from both classical epidemiology and dynamical epidemiology to both characterize and understand the transmission process. Many participants were well trained in dynamical epidemiology, but lacked strong empirical skills. Thus, we placed an emphasis on understanding all the steps from data generation to analysis [25]. Participants learned to clean raw datasets, putting them into analyzable form, formed small groups and chose one or more research goals, and then selected the appropriate methods to achieve those goals (Table 1), thereby learning concepts such as confounding, selection bias, information bias, and random error as they proceeded to identify risk factors for disease exposure (Box 2; Figure 2). In order to collect data for a retrospective cohort study participants crafted surveys (Text S5), learning aspects of survey design. Participants conducting mathematical analyses learned the relationship between susceptible depletion and epidemic fadeout (Box 2; Figure 1), simulated the effect of vaccination or immunity on outbreak dynamics (Figure 3), and explored how heterogeneity in disease network structure affects transmission dynamics and, subsequently, disease incidence. Groups then presented their results, describing their goals, methods, results, an interpretation of these results, and any shortcomings. Importantly, these presentations revealed methods' assumptions and utilities, clarifying how the various risk factors and mathematical methods differ from and complement each other. After their presentations groups were asked various questions to assess their understandings of the various concepts and methods presented (Box 3). A complete how to description of the exercise (including R scripts for reproducing the various projects, Figures 1–3, the necessary datasets, and other analyses) and additional variations thereof are given in Texts S3, S4 and Datasets S1, S2, S3, S4, S5, S6, S7.

**Table 1:** Examples of epidemiological methods that can be used during the exercise.

Field	Study	Analysis	Data	Goal	Pedagogical Value
Risk factor	Case-control or cohort	Logistic or Poisson regression (generalized linear model)	Disease outcomes, risk factors	Understand what variables increase (or decrease) risk of disease/infection	Exposure to survey design, data entry and cleaning, and statistical methods; understand effects of confounding and bias in data analysis
Mathematical	Estimation of *R*_0_ and infectious period	Probability distribution fit to infectious contact data and infectious periods	Contact tracing data and observed infectious periods	Characterize individual heterogeneity in infectiousness and overall pathogen contagiousness	Understand utility of contact tracing data in characterizing disease dynamics, understand *R*_0_ as an epidemic threshold, understand how individual variation can affect disease dynamics
	Outbreak simulation	Stochastic SIR individual-based model with Gillespie algorithm	Estimates of *R*_0_, incubation and infectious periods	Understand how outbreak size is affected by immune proportion	Awareness of effects of stochasticity in outbreaks of small sizes, gain intuition for how simulation can be used to answer applied questions

Further explanation and R scripts are provided in Texts S1, S3, and S4.

**Box 2. Concepts at a Glance**

**Understanding Concepts in the Natural History of a Disease**

- Introduction to the concepts of incubation, latency, infectiousness, being a/symptomatic, virulence, pathogenicity, immunity, transmissibility, pathogen evolution

**Classical Epidemiology**

- Epidemiological study designs (e.g., case-control and cohort studies)
- Outbreak investigation methodology (case definition, contact tracing, epidemic curves)
- Measures of effect (e.g., odds ratios, relative risk)
- Confounding, bias, and interaction

**Dynamical Epidemiology**

- Introduction to a simple Susceptible-Infected-Recovered (SIR) model
- Introduction to concepts of the basic and effective reproduction numbers, attack rate, and herd immunity
- Using dynamic models to answer public health questions (e.g., using models as a means to explore counterfactual instances of disease occurrence)

**Biostatistics and Probability**

- Probability distributions and generation of random variables
- Regression, confidence intervals, and hypothesis testing
- Parameter estimation (e.g., maximum likelihood estimation)

**Practical Experience with Broad Application**

- Questionnaire design
- Data collection, cleaning, visualization, and analysis
- Verbal communication skills (i.e., presentation of results)

**Box 3. Evaluation Tools**

**Questions to gauge knowledge and understanding of the exercise**

***For students with little or no prior knowledge or technical training (i.e., high school level and early undergraduates)***

- What is the difference between an infectious disease and a communicable disease?
- How might you collect information on a disease outbreak?
- What might cause an outbreak to end?

***For students with some prior knowledge and/or technical training (i.e., advanced undergraduates and graduate students)***

- What determines how many cases occur in an outbreak?
- What determines how long an outbreak lasts and when the peak occurs?
- Why aren't data a perfect representation of reality?
- Is it possible to predict whether an epidemic will occur when a pathogen is introduced into a population?
- How and why might an individual's risk of infection change over the course of an outbreak? When is average individual risk the highest?
- What individuals are most likely to be infected in an outbreak of communicable disease?
- Why do some pathogens cause epidemics while others do not?
- Why don't all individuals in a population have to be vaccinated to prevent an epidemic?

**Evaluative activities**

***For students with little or no prior knowledge or technical training***

- Have students describe the life cycle of MMF by matching infectious disease terms (such as “latent period” and “transmission event”) to aspects of the exercise and discussing in relation to a real pathogen
- Have students plot and explain data (e.g., the epidemic curve, the cumulative incidence through time, the distribution of infectious contacts, latent periods, and infectious periods)

***For students with some prior knowledge and technical training***

- Have students describe the epidemic curve, explain differences in data collection that might influence aspects of the observed curve (e.g., a case definition that relies on symptoms or reporting)
- Have students estimate parameters (such as those describing the latent and infectious period distributions) on a dataset from another source
- Have students pick a real immunizing infection with available estimates of the latent period, infectious period, and transmissibility (*R_0_*) are available and then reparameterize the stochastic simulations using the code available or their own code to compare the expected dynamics of MMF and their chosen pathogen
- Have students discuss what aspects of the epidemic dynamics and data collection determine the ease with which a classical epidemiology study can detect which individual-level risk factors are associated with a higher probability of infection.
- Have students conduct exercises/analyses as described and present on their projects, including data collection, cleaning, and analysis as well as any unexpected difficulties they encountered

## Conclusion

This educational approach provides a much-needed conceptual integration of risk factor and mathematical approaches in epidemiological training, illuminating their strengths, weaknesses, and how they complement each other. Further, this exercise is of interest and understandable to students in other fields. Much of the exercise is simple enough to be performed by adequately trained high school students and could therefore even serve as an early introduction to infectious disease epidemiology. The exercise is also perfectly suited to undergraduate or graduate courses in epidemiology, infectious diseases, public health, biomathematics, computational biology, statistics, and nonlinear dynamics. Such tools will stimulate training of epidemiologists able to think from both the classical and dynamical perspectives. Particularly in developing countries where training is only slowly becoming more interdisciplinary, exercises such as MMF teach participants how subfields of public health complement each other and produce professionals more able to collaborate across disciplines.

The most recent updates to supplementary material as well as an online webpage for running the epidemic without use of R are available at http://lalashan.mcmaster.ca/theobio/mmed/index.php/MMF.

## Supporting Information

Dataset S1 **Line list of MMF infections from MMED 2010.**
(CSV)

Dataset S2 **Contact tracing data from MMED 2010.**
(CSV)

Dataset S3 **Line list of MMF infections from MMED 2011.**
(CSV)

Dataset S4 **Contact tracing data from MMED 2011.**
(CSV)

Dataset S5 **Risk factor data from MMED 2011, collected using the survey in Text S5.**
(CSV)

Dataset S6 **Risk factor data from MMED 2010.**
(CSV)

Dataset S7 **Infectious period data from the MMF outbreak at MMED 2011.**
(CSV)

Figure S1 **Lecture slide 1: introduction to disease dynamics.**
(PDF)

Figure S2 **Lecture slide 2: introduction to dynamics of vector-borne diseases.**
(PDF)

Figure S3 **Lecture slide 3: demographic stochasticity.**
(PDF)

Figure S4 **Lecture slide 4: study design and analysis in epidemiology.**
(PDF)

Figure S5 **Lecture slide 5: introduction to statistical philosophy.**
(PDF)

Figure S6 **Lecture slide 6: introduction to likelihood.**
(PDF)

Figure S7 **Lecture slide 7: likelihood fitting and dynamic models I.**
(PDF)

Figure S8 **Lecture slide 8: likelihood fitting and dynamic models II.**
(PDF)

Text S1 **This file includes a glossary and detailed information for instructors on implementation of the exercise, including ideas for development of group projects and description of possible variations.**
(DOC)

Text S2 **The infection notification form used as the infectious agent.** Before use, the form should be tailored to the specific course or classroom setting by adjusting the parts of the form highlighted in yellow. Instructors may also want to change the name of the “disease” to fit the context of their course.
(DOC)

Text S3 **Code for producing the figures in the article.**
(TXT)

Text S4 **Code for conducting analyses described in Text S1.**
(TXT)

Text S5 **Survey developed by MMED participants following the 2011 MMF outbreak to gather data on potential risk factors.**
(PDF)

## Abbreviations

MMED: Meaningful Modeling of Epidemiological Data
MMF: Muizenberg Mathematical Fever
