# Supplementary material for: How to Make Epidemiological Training Infectious
Source: PLoS Biol. 2012 Apr 3;10(4):e1001295. doi: 10.1371/journal.pbio.1001295 (PMC3317897; doi:10.1371/journal.pbio.1001295)
Supplement: Figure S1 — Lecture slide 1: introduction to disease dynamics. (PDF) [file pbio.1001295.s008.pdf]

SOME RIGHTS RESERVED

This presentation is made available through a Creative Commons Attribution license.
Details of the license and permitted uses are available at
http://creativecommons.org/licenses/by-nc/3.0/

© 2010 Dr. Juliet Pulliam

Title: Introduction to Infectious Disease Dynamics, Part I
Attribution: Dr. Juliet Pulliam, Topics in Biomedical Sciences
Source URL: http://lalashan.mcmaster.ca/theobio/mmed/index.php/Honours Course

For further information please contact Dr. Juliet Pulliam (juliet.mmed.clinic@gmail.com).

AIMS African Institute for Mathematical Sciences

Introduction to infectious disease dynamics I

Dr. Juliet Pulliam
RAPIDD Program
Division of International Epidemiology
Fogarty International Center
National Institutes of Health (USA)

Introduction to Biomedical Sciences
BSc Honours Course in Biomathematics
African Institute for the Mathematical Sciences
Muizenberg, South Africa
17 May 2010

Infectious diseases

Terminology

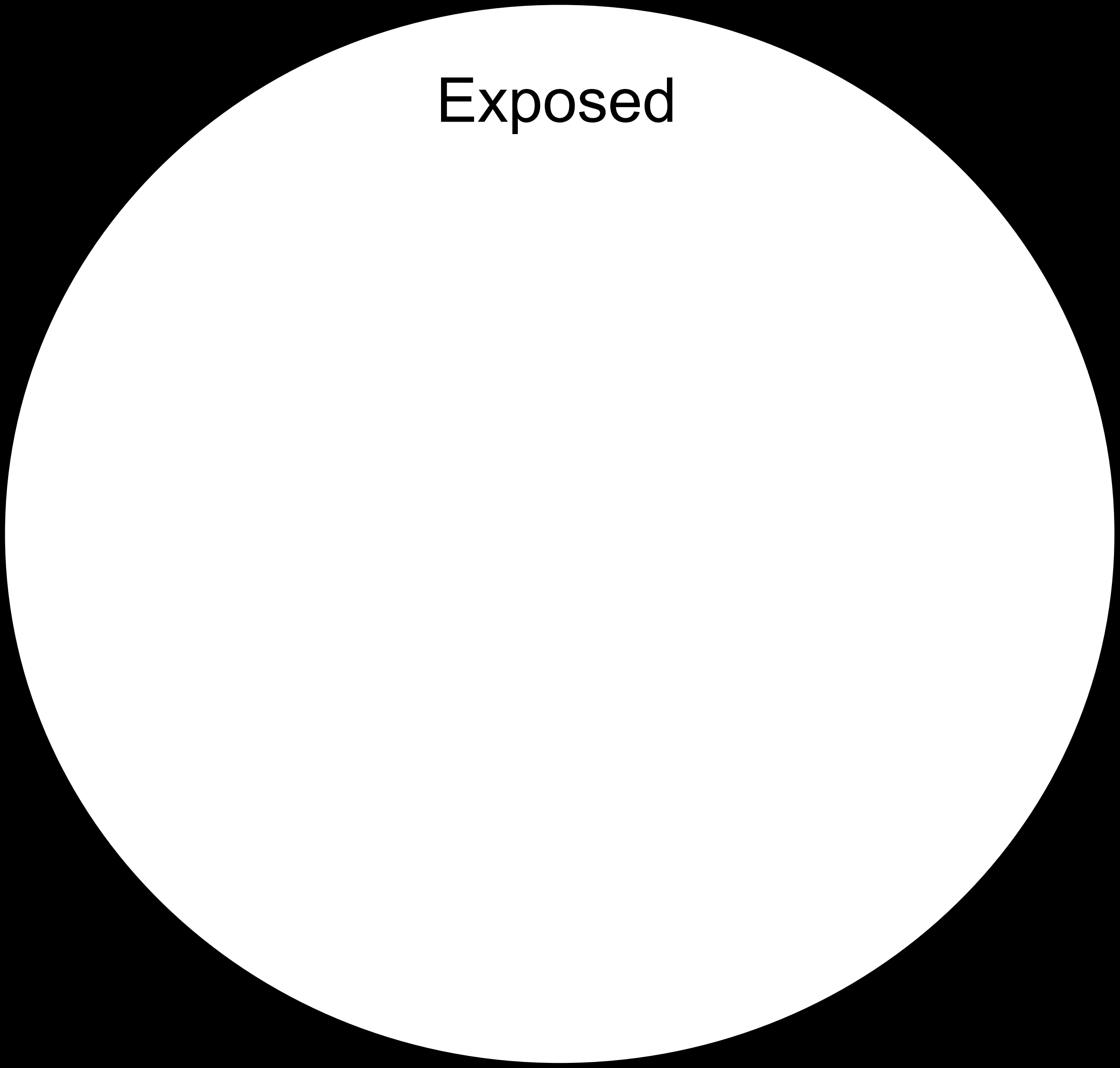

Exposure - potentially infectious contact between a parasitic organism and a potential host

Infectious diseases

Terminology

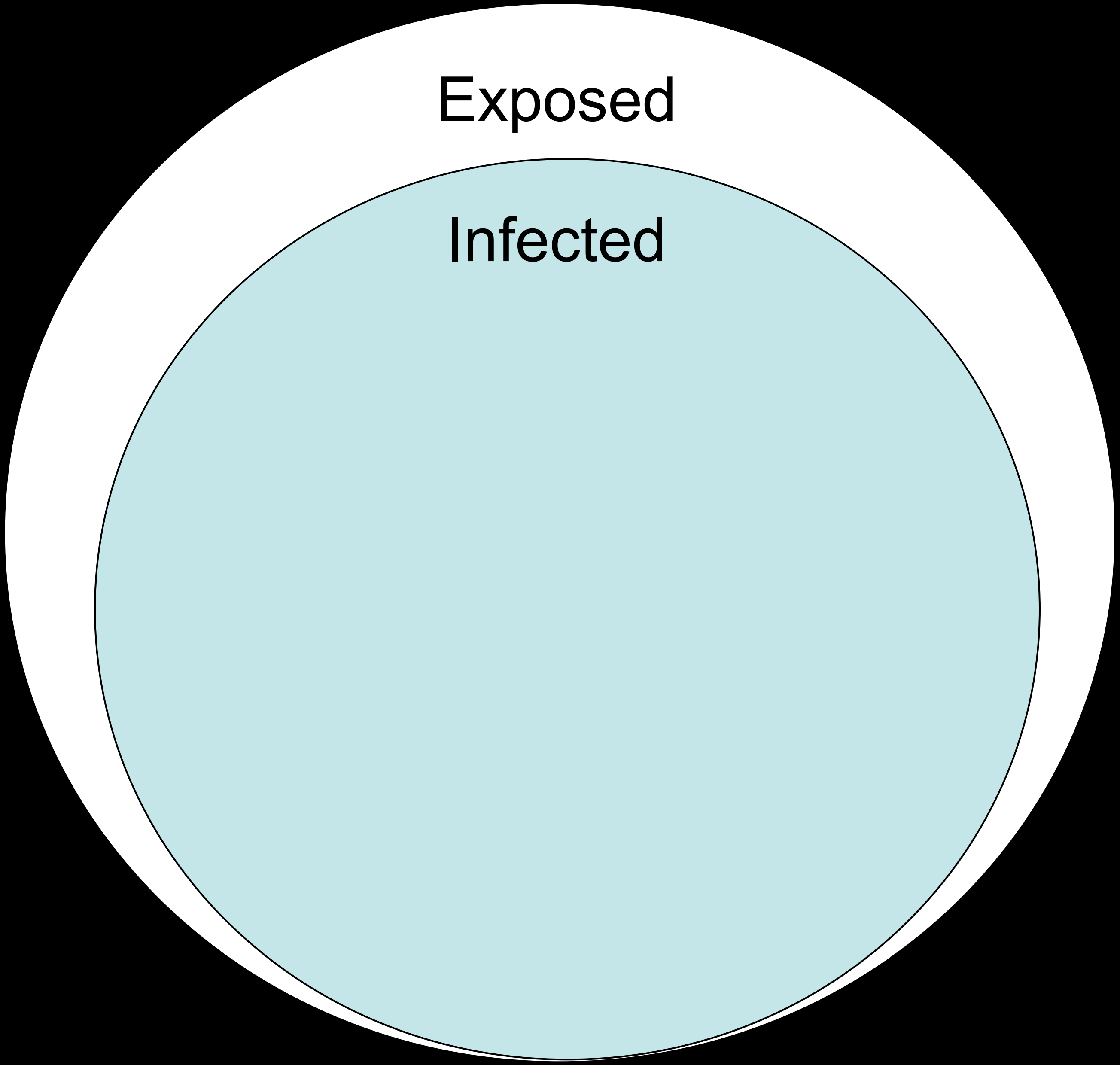

Infection - a parasitic organism enters a host

Infectious diseases

Terminology

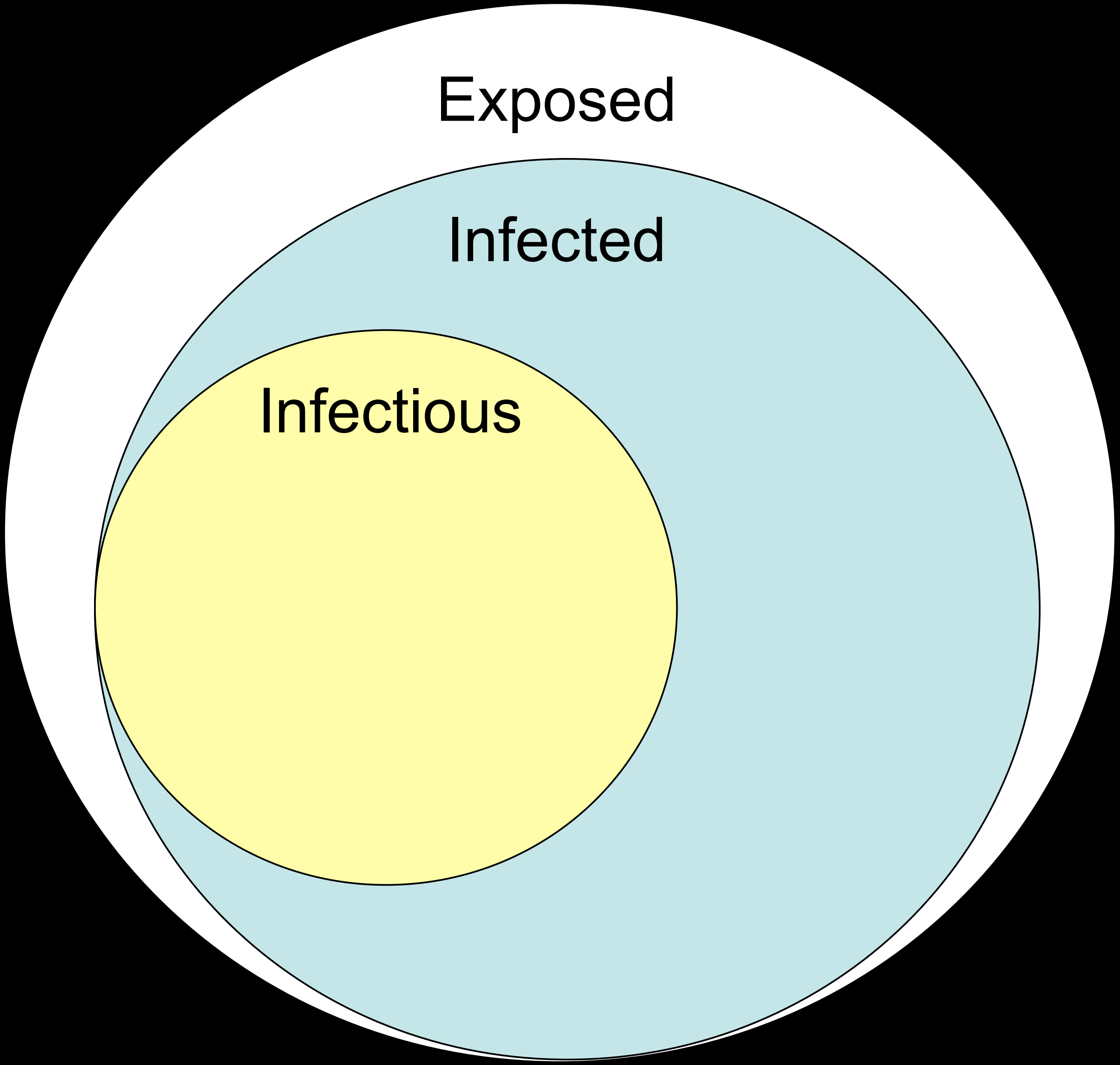

Transmission - a parasitic organism replicates within an infected host, and the infected host sheds infectious individuals (the host is said to be “infectious” or “transmitting”)

Terminology

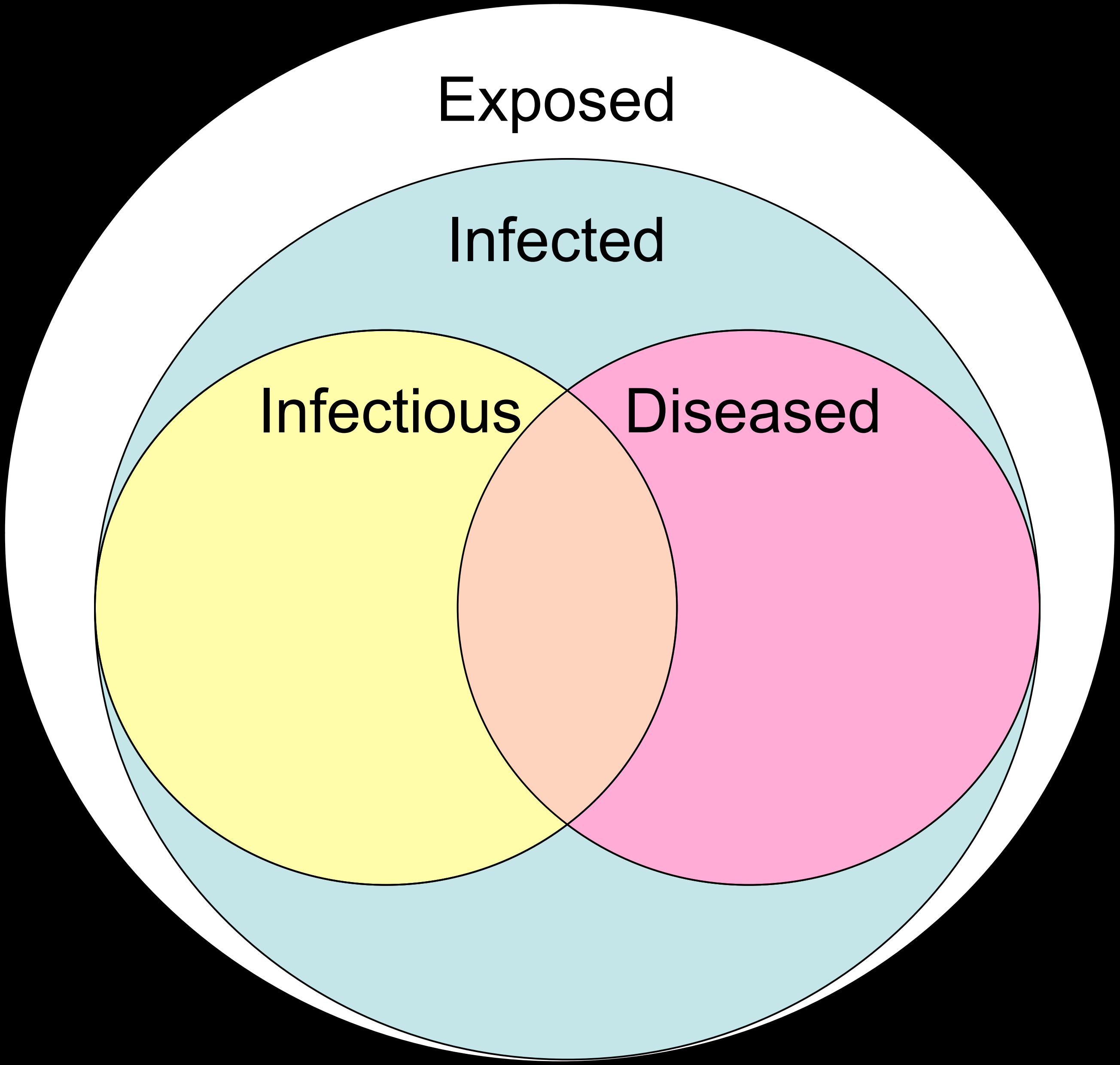

Pathogenesis - a parasitic organism causes changes in the physiology of an infected host, affecting survival and/or reproduction (the host is said to be "diseased")

Infectious diseases

Terminology

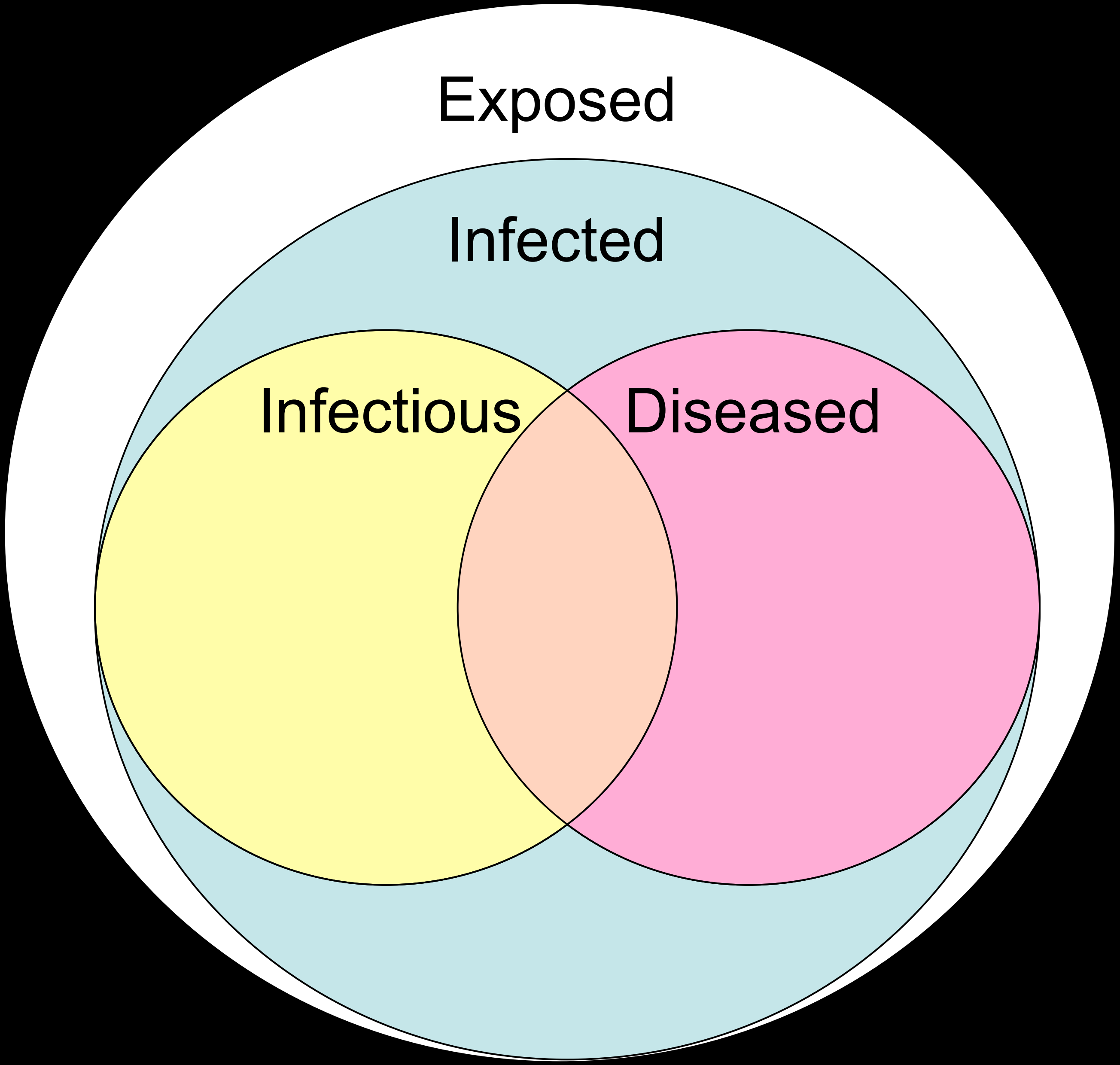

Infectious disease dynamics are determined -- to a large extent -- by the duration of these states, as well as the degree and timing of overlap

Infectious diseases

Terminology

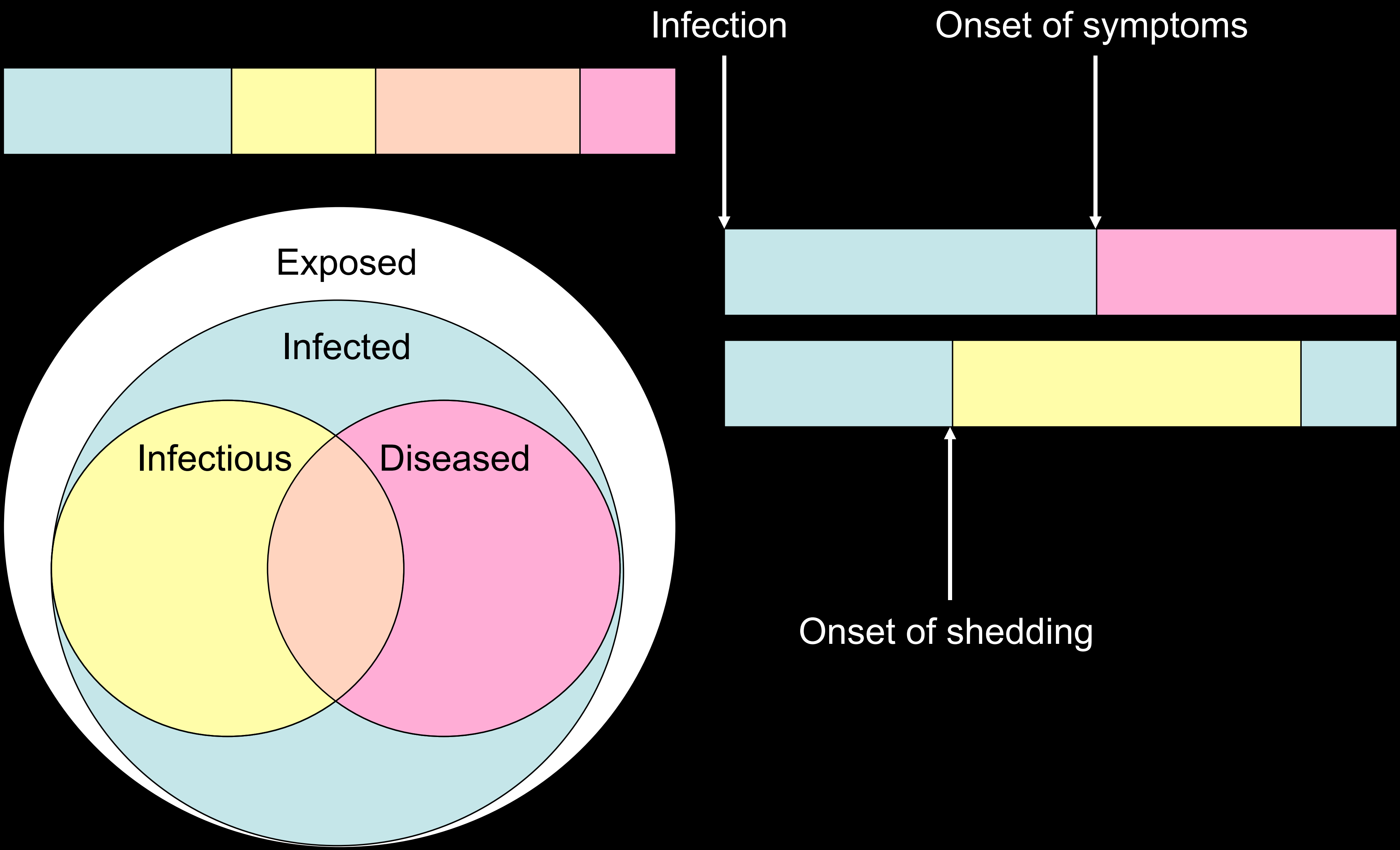

Infectious diseases

Terminology

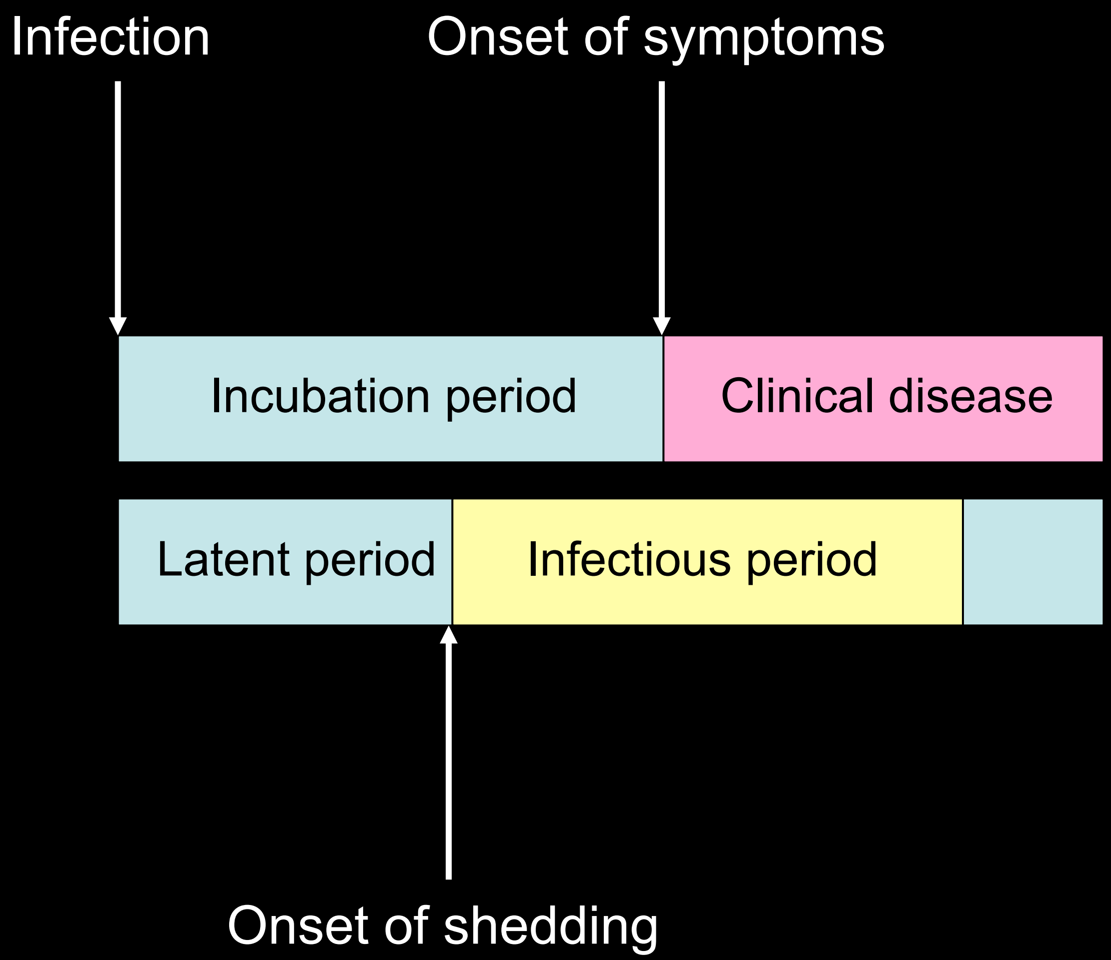

Infectious diseases

Terminology

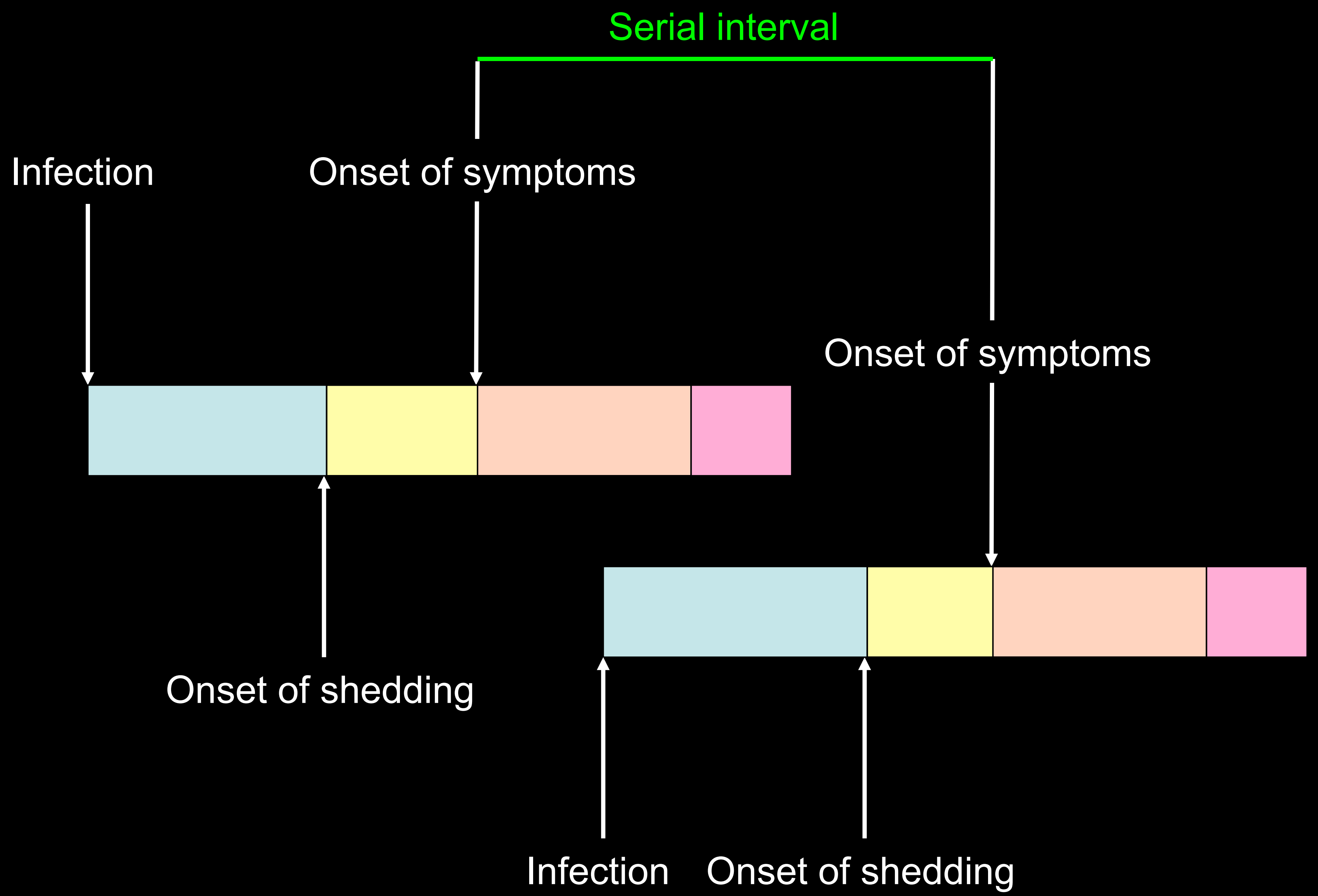

Infectious diseases

Transmission

Mode of transmission

Direct transmission

Direct contact

Droplet spread

Indirect transmission

Airborne

Vehicle-borne (fomites)

Vector-borne (mechanical or biological)

Portal of entry

Portal of exit

Immunity

Active immunity

Requires stimulation of antibody production

Response to many infections

Induced by most vaccines

Long-lasting

Passive immunity

Presence of antibodies without stimulating production

Maternal antibodies

Immunoglobulin injections

Transient

Infectious disease dynamics

Traditional epidemiology

Outbreak investigation

Disease surveillance

On-the-ground management

Mathematical epidemiology

General principles

Explanation of patterns

Prediction of spread and control

Assessment of necessary data

Terminology

Endemic level of disease
(“hyperendemic”)

Epidemic

Disease outbreak

Pandemic

Sporadic cases and clusters

Shoe-leather epidemiology

Epidemic curves

Common source epidemics

point source exposure

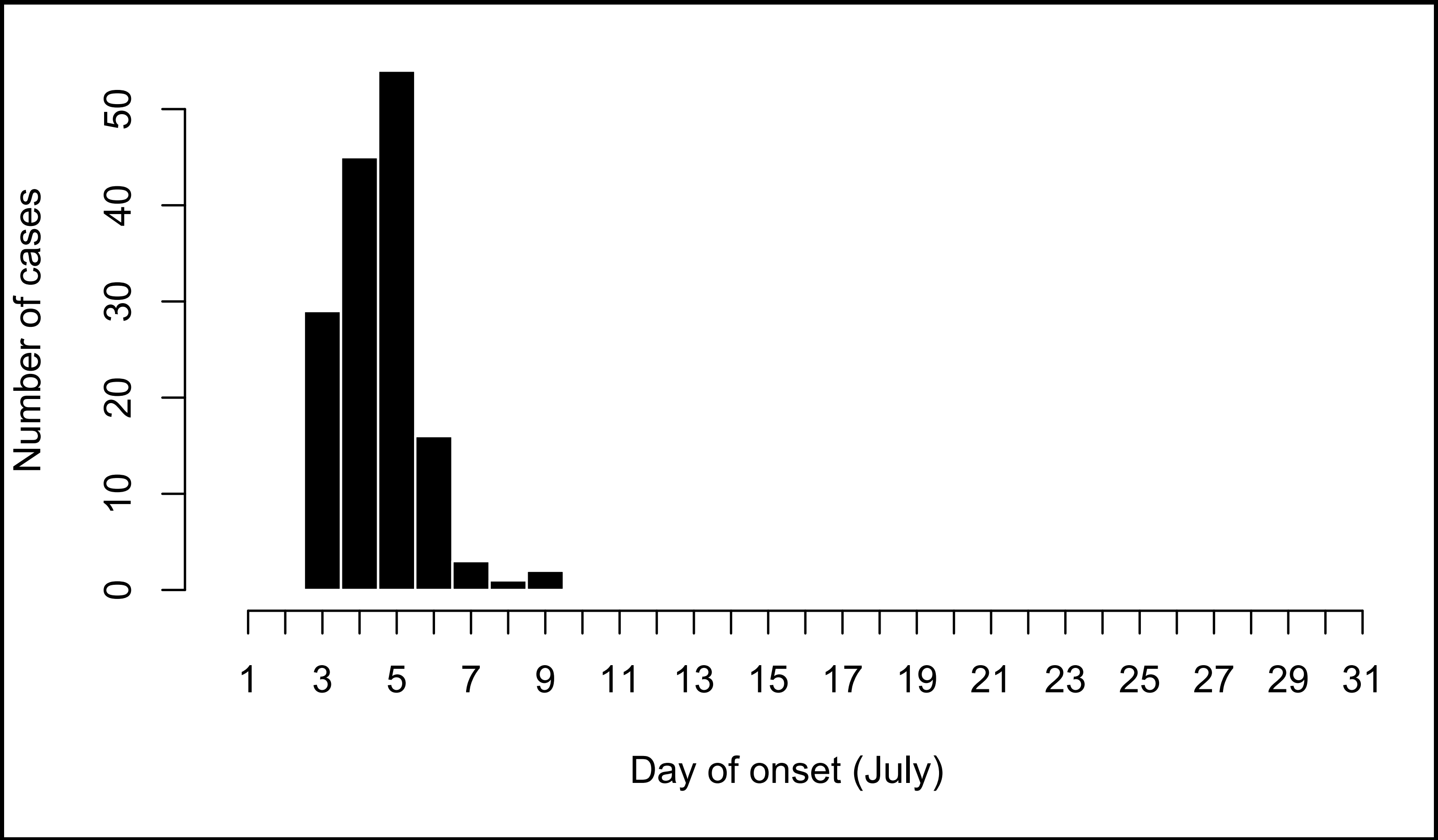

Shoe-leather epidemiology

Epidemic curves

Common source epidemics

continuous exposure

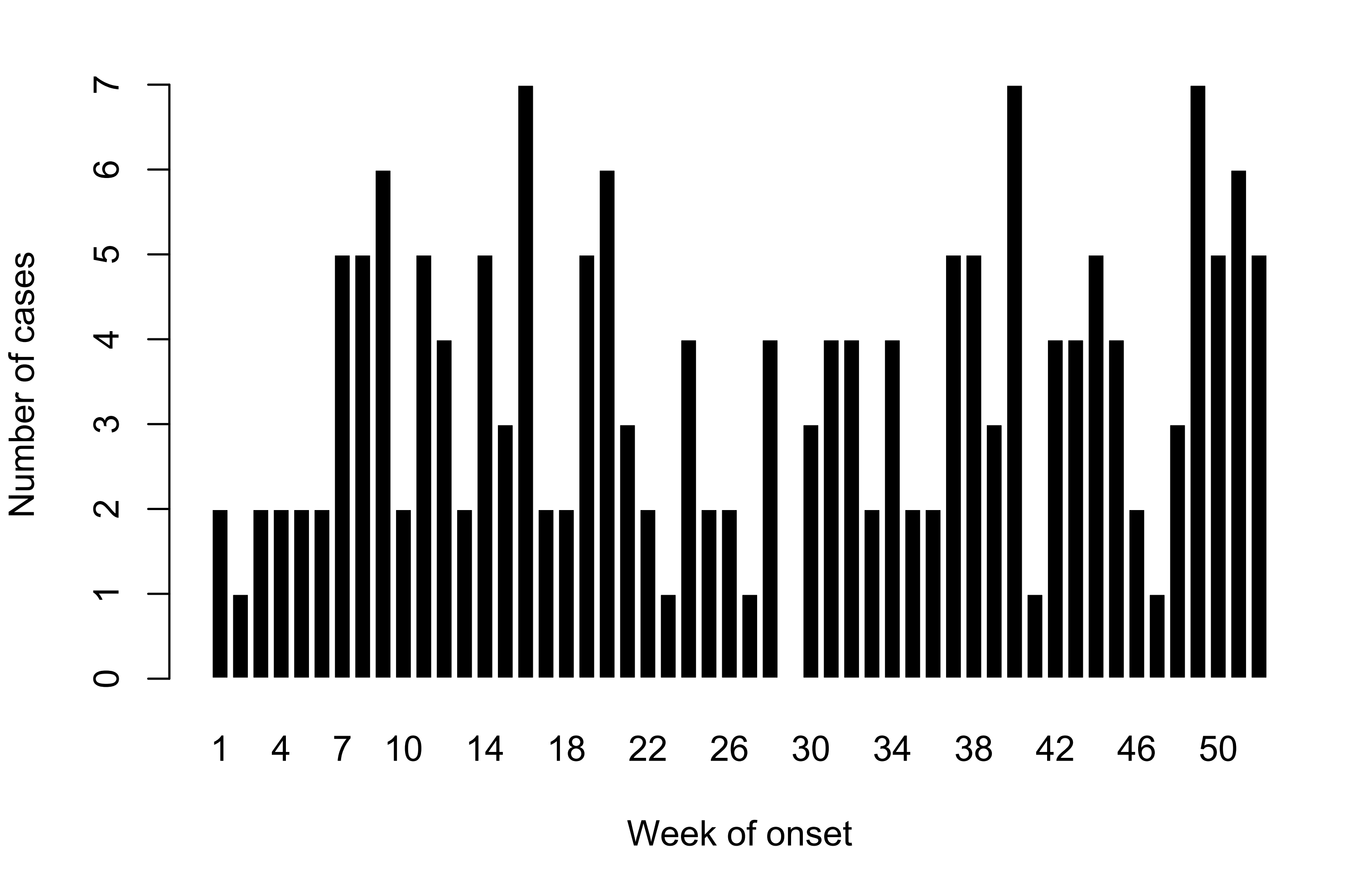

Shoe-leather epidemiology

Epidemic curves

Common source epidemics

intermittent exposure

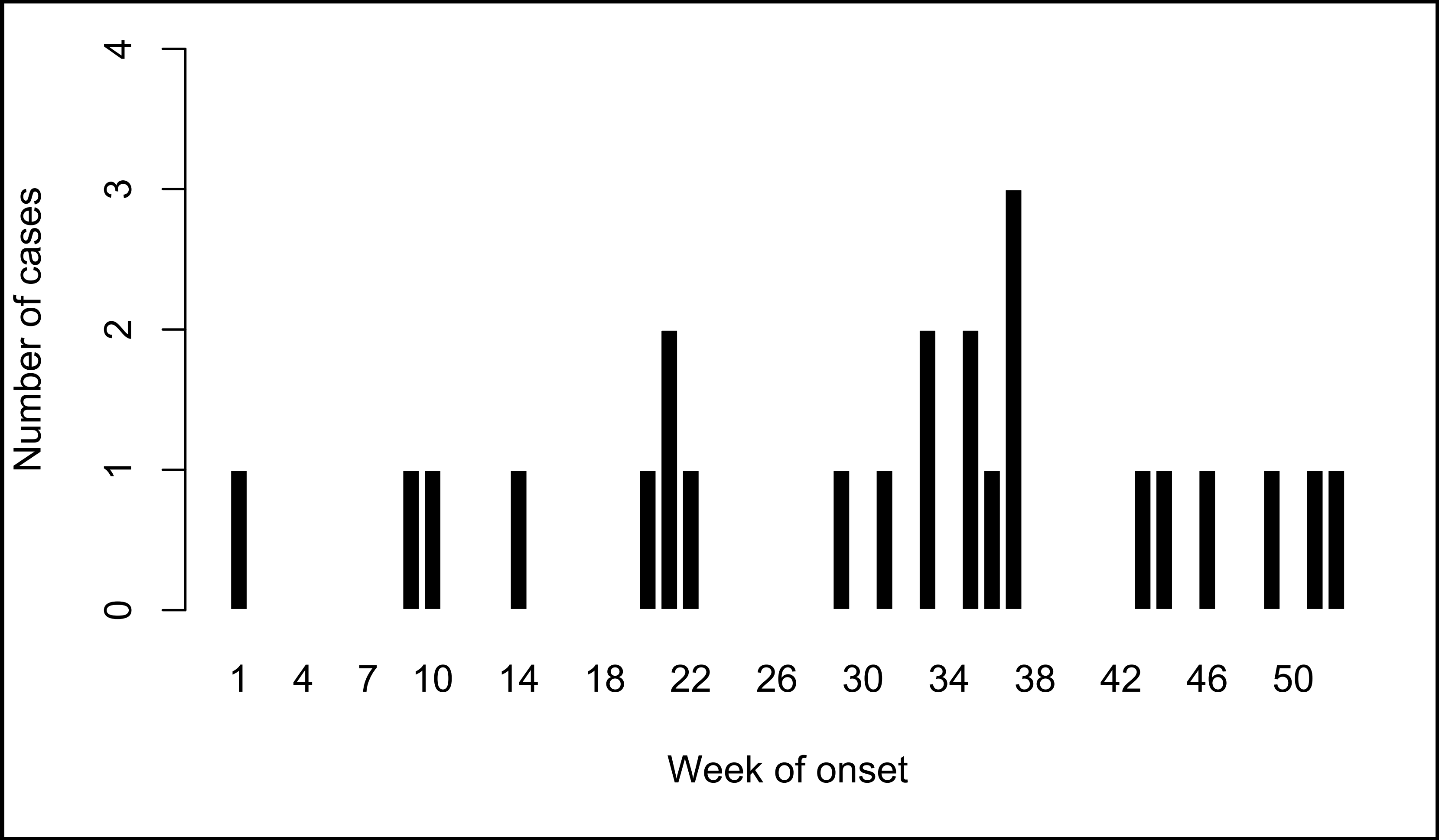

Shoe-leather epidemiology

Epidemic curves

Propagated epidemics

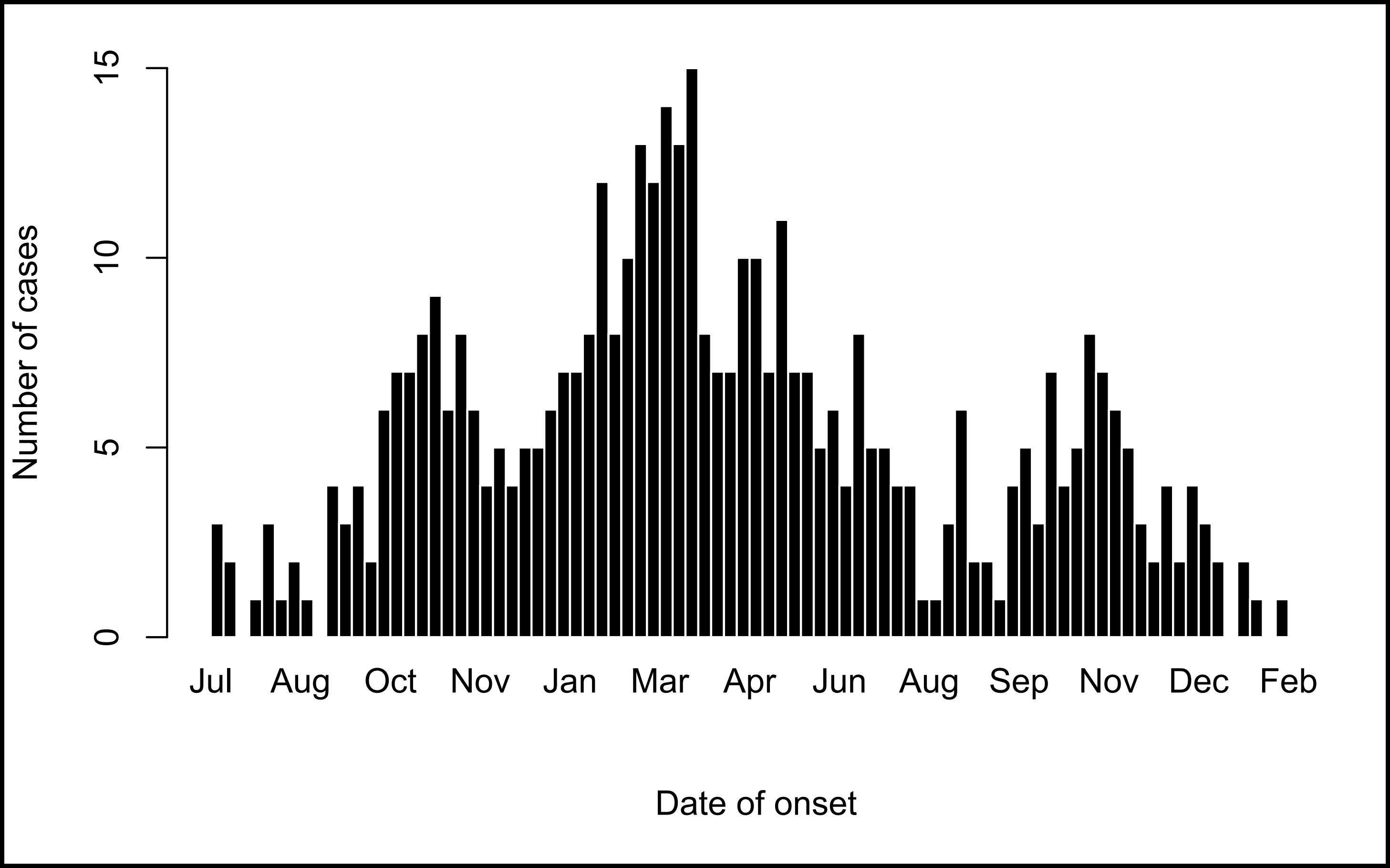

Simulated epidemic with R_0=1.2 in a population of 1500 individuals

SOME RIGHTS RESERVED

This presentation is made available through a Creative Commons Attribution license. Details of the license and permitted uses are available at http://creativecommons.org/licenses/by-nc/3.0/

© 2010 Dr. Juliet Pulliam

Title: Introduction to Infectious Disease Dynamics, Part II
Attribution: Dr. Juliet Pulliam, Topics in Biomedical Sciences
Source URL: http://lalashan.mcmaster.ca/theobio/mmed/index.php/Honours Course

For further information please contact Dr. Juliet Pulliam (juliet.mmed.clinic@gmail.com).

AIMS African Institute for Mathematical Sciences

Introduction to infectious disease dynamics II

Dr. Juliet Pulliam
RAPIDD Program
Division of International Epidemiology
Fogarty International Center
National Institutes of Health (USA)

Introduction to Biomedical Sciences
BSc Honours Course in Biomathematics
African Institute for the Mathematical Sciences
Muizenberg, South Africa
17 May 2010

Infectious disease dynamics

Traditional epidemiology

Outbreak investigation

Disease surveillance

On-the-ground management

Mathematical epidemiology

General principles

Explanation of patterns

Prediction of spread and control

Assessment of necessary data

Infectious diseases

Terminology

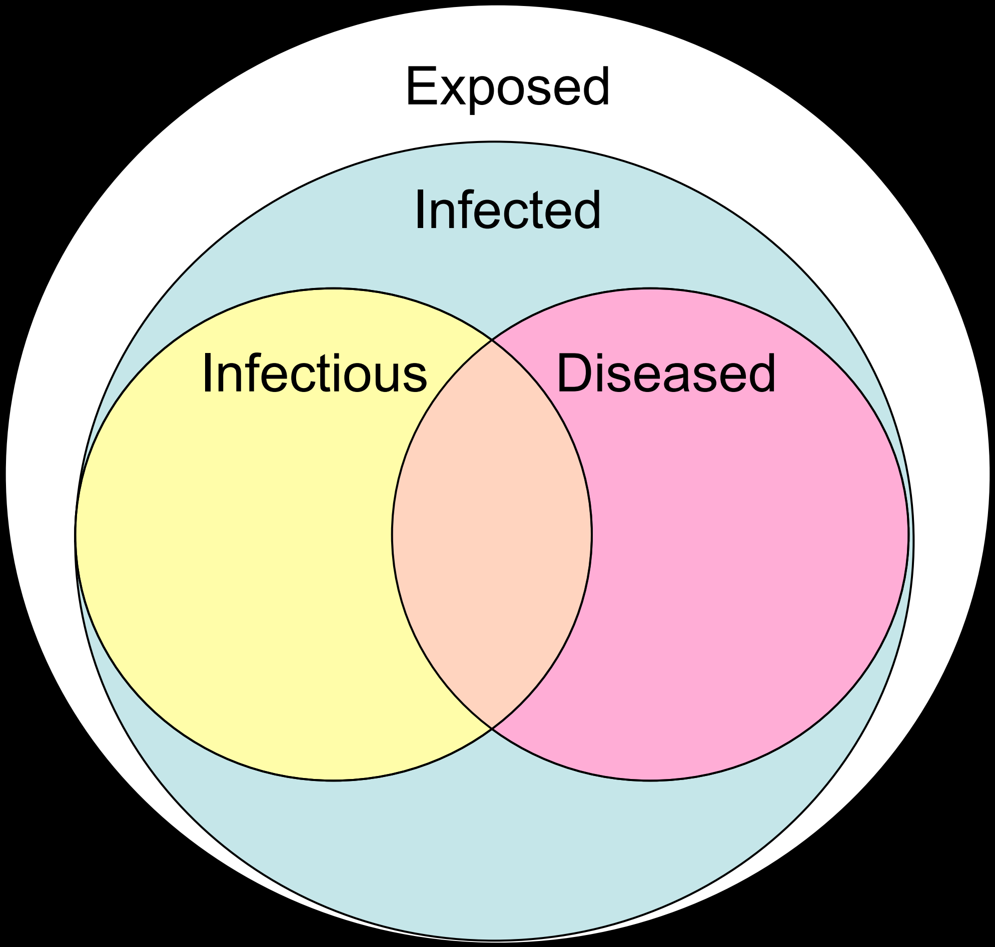

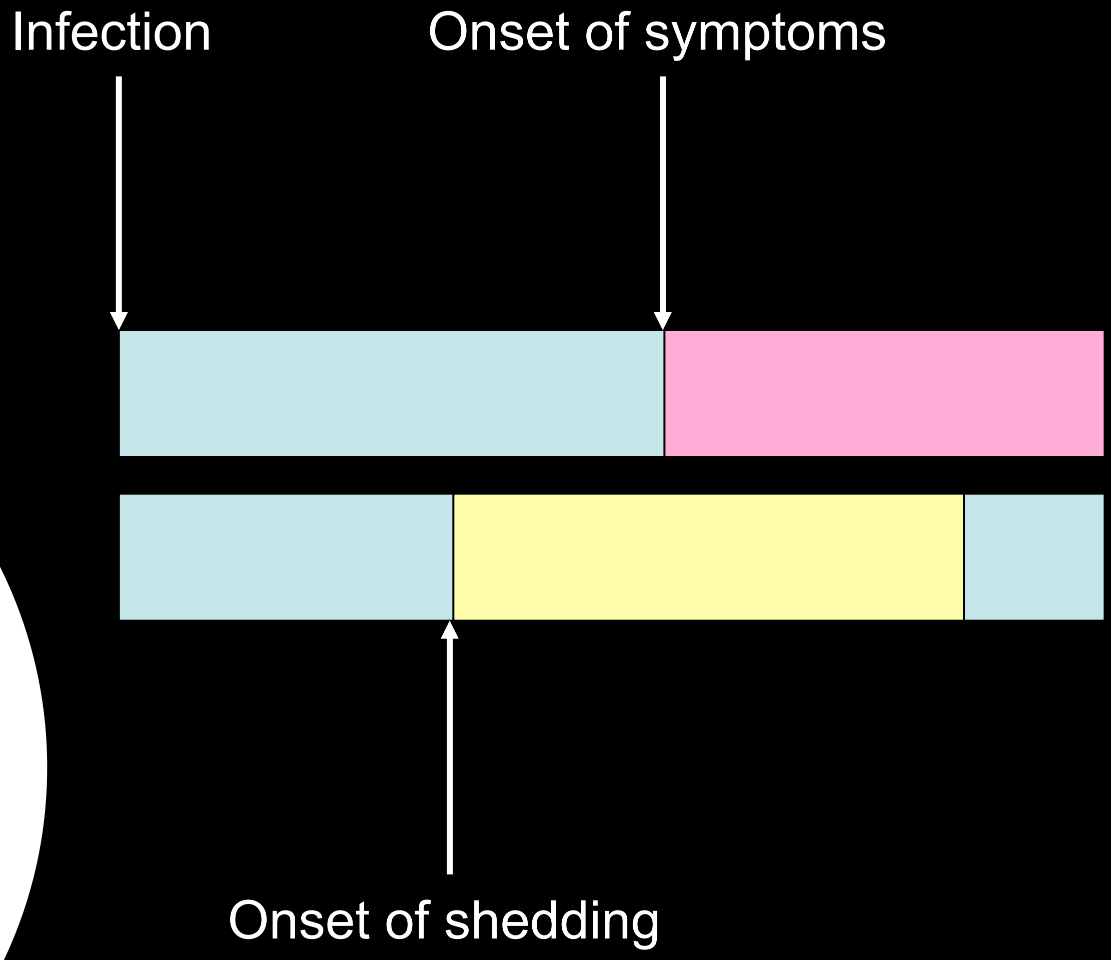

A simpler view of the world

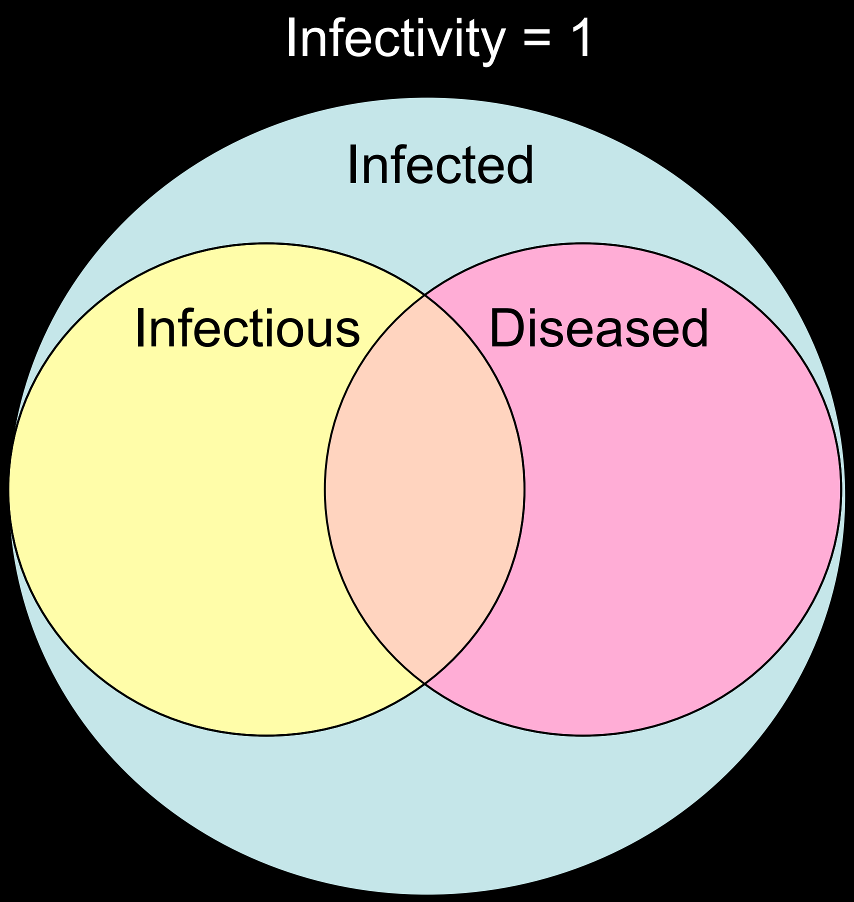

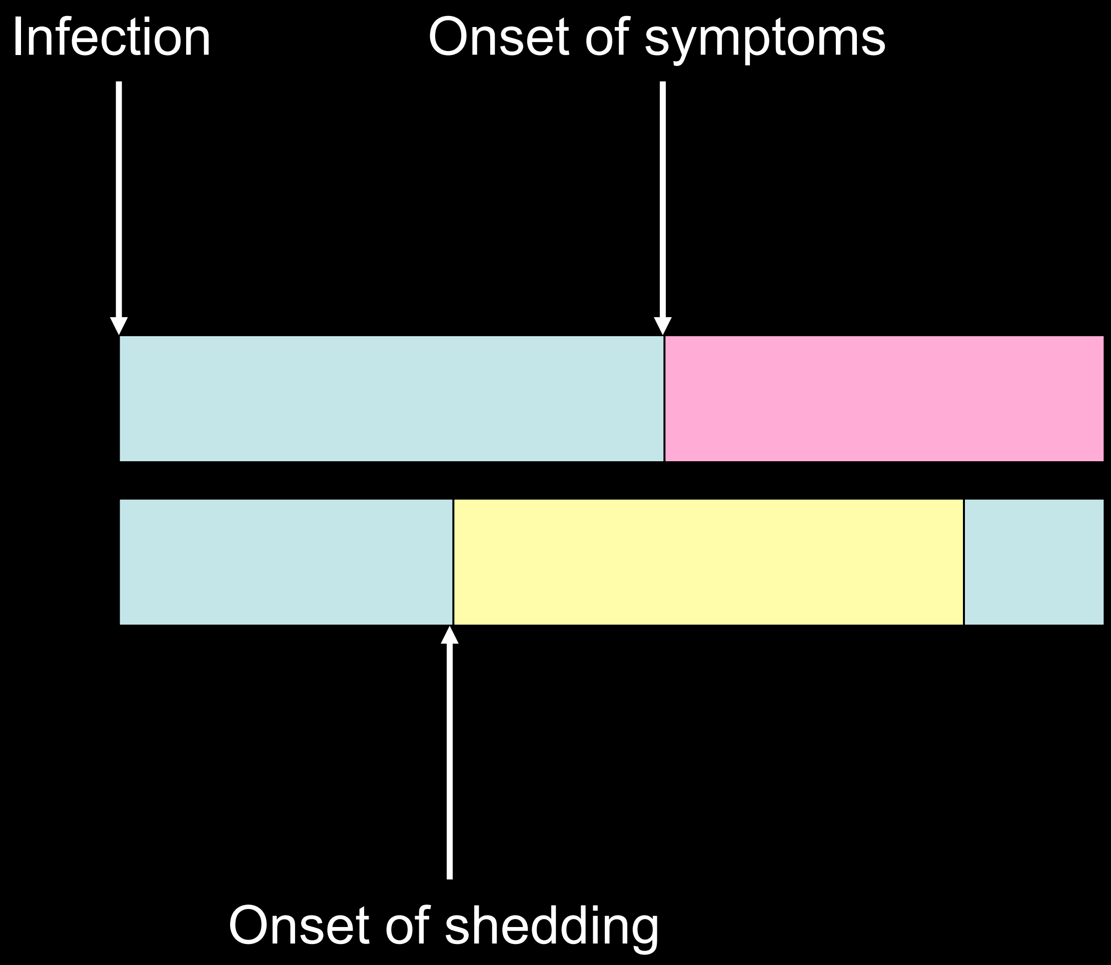

Infectious diseases

A simpler view of the world

Infectivity = 1

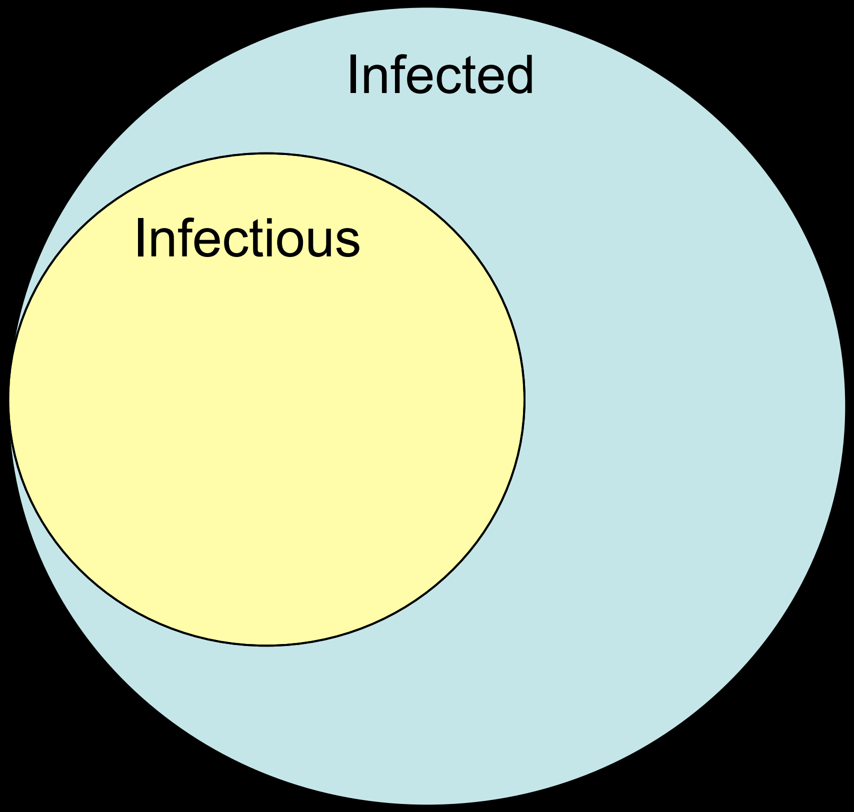

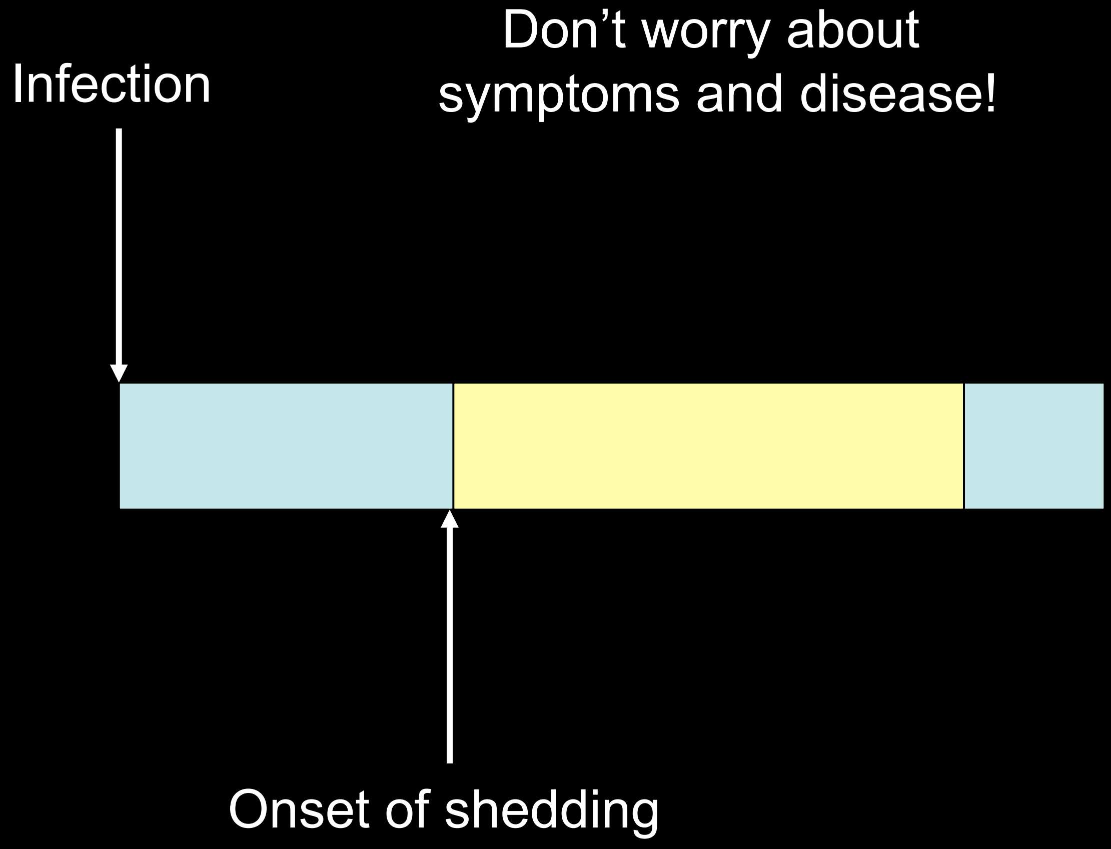

Infectious diseases

A ^much! simpler view of the world

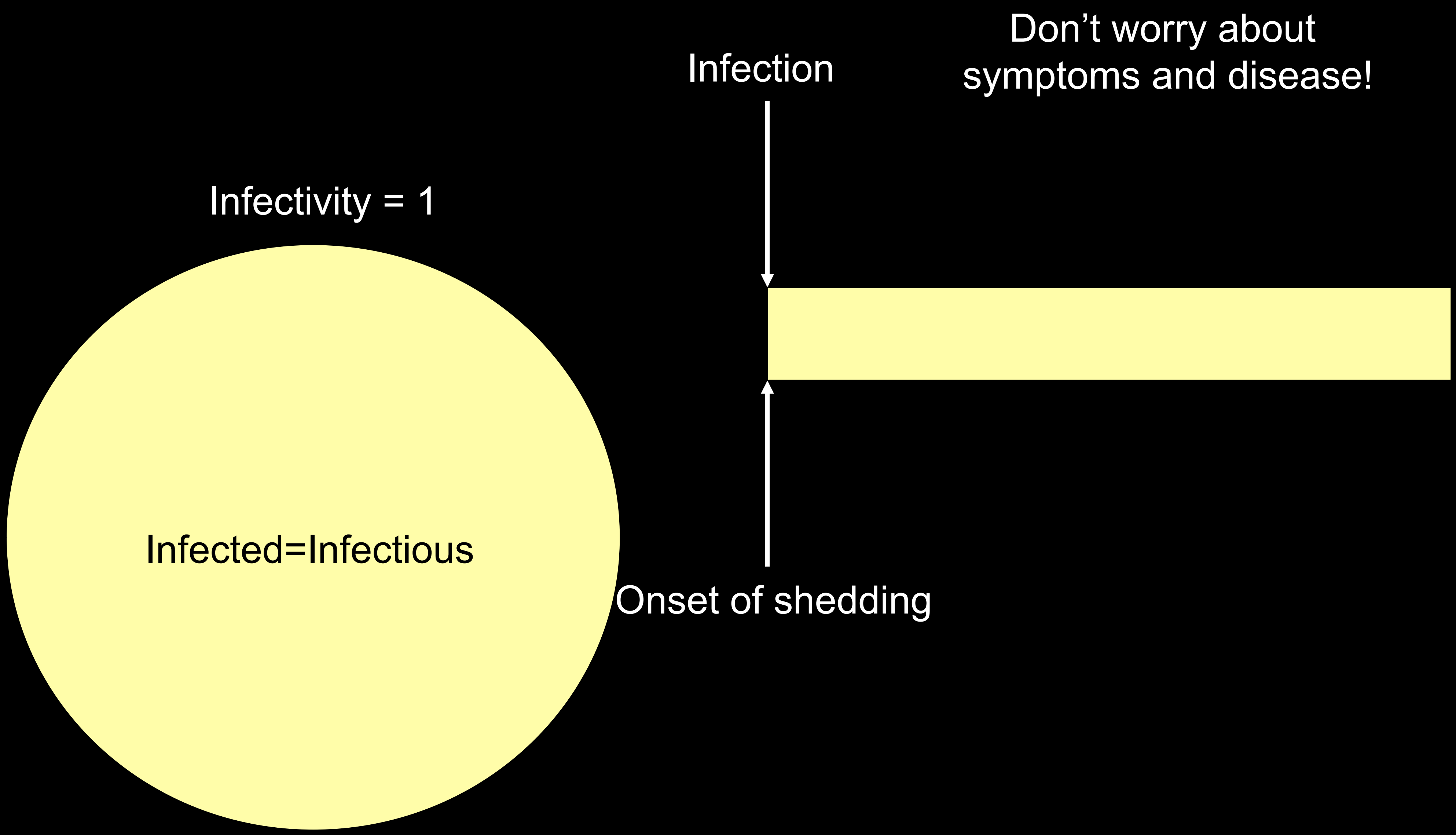

A much! simpler view of the world

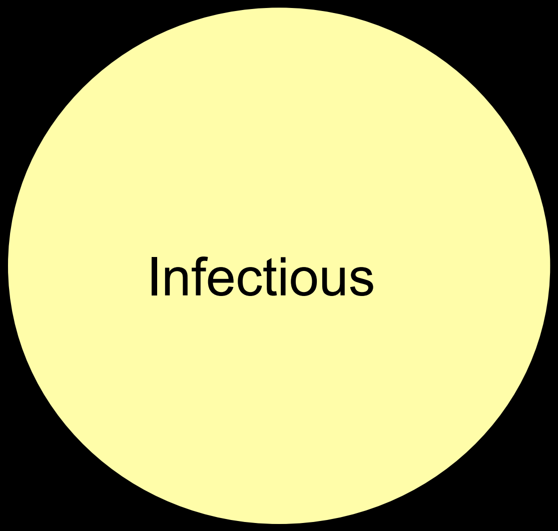

A simple view of the world

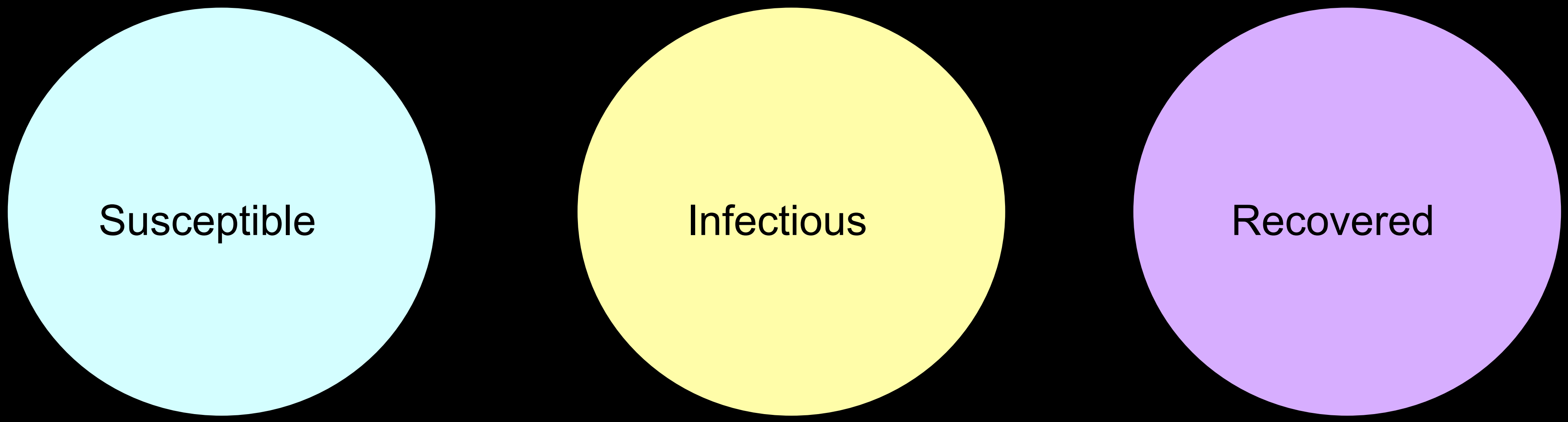

A simple view of the world

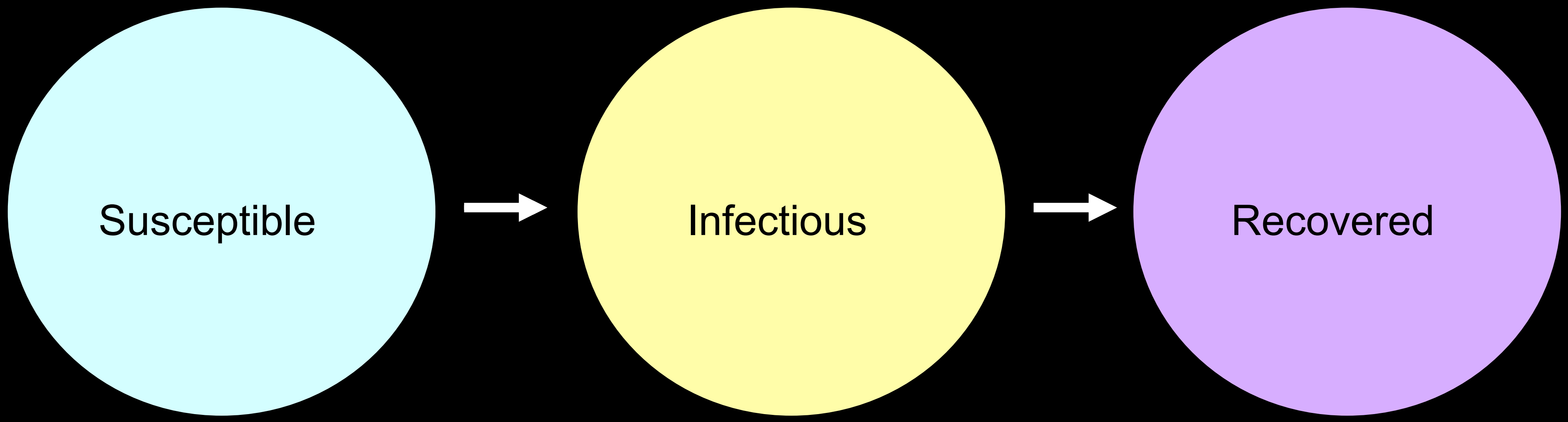

A simple view of the world

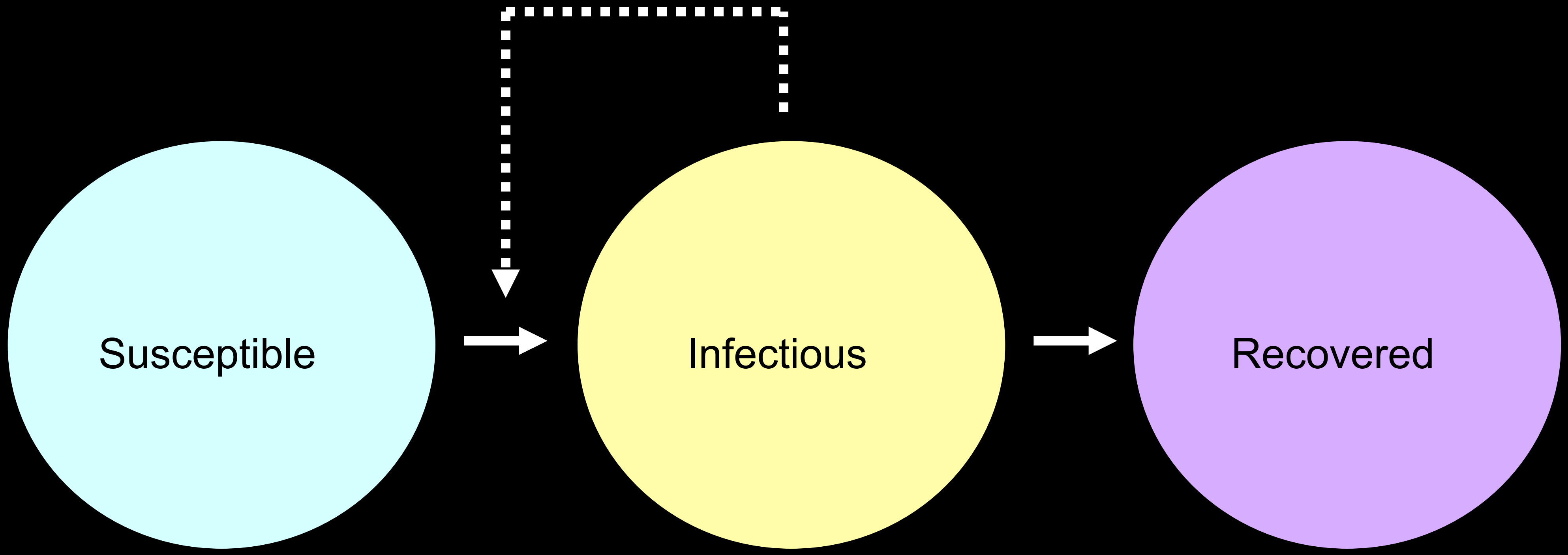

Health-related states

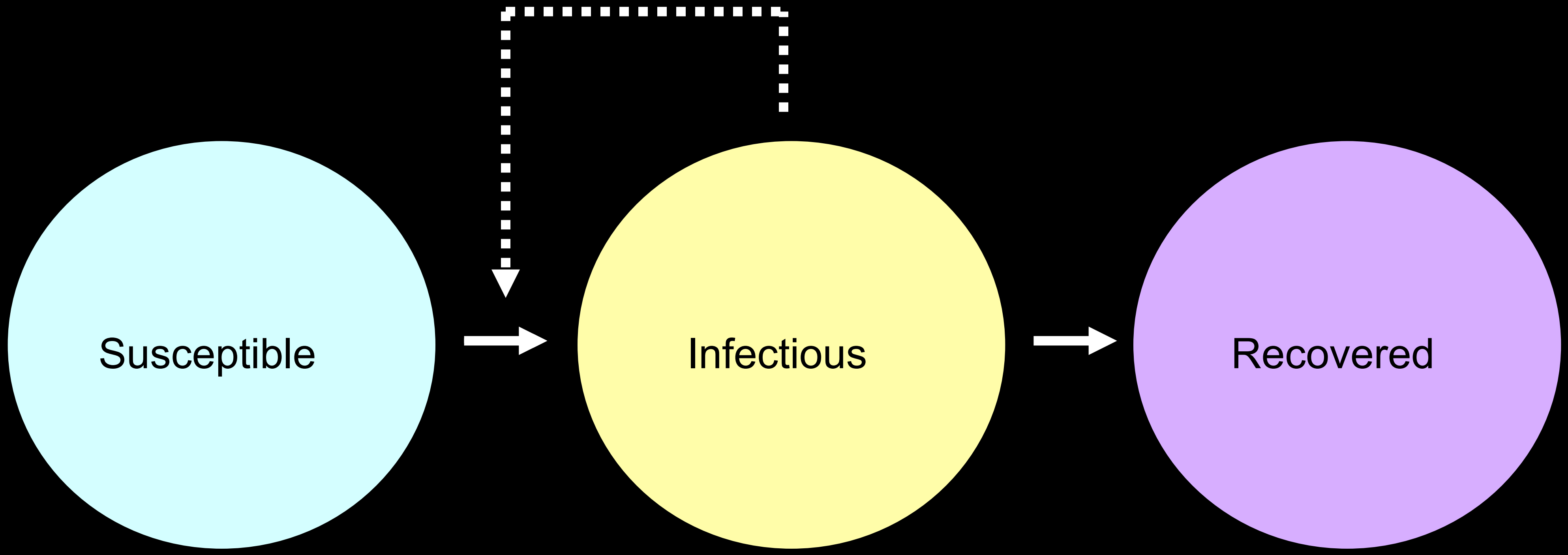

State variables

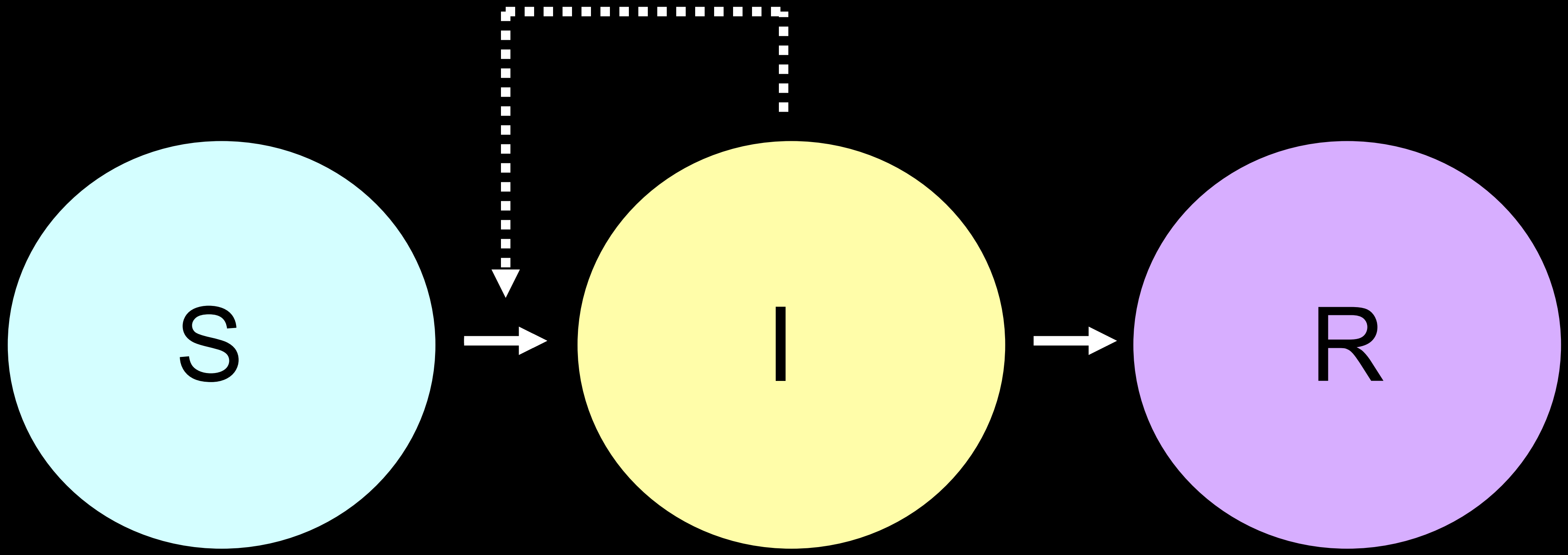

Transition rates

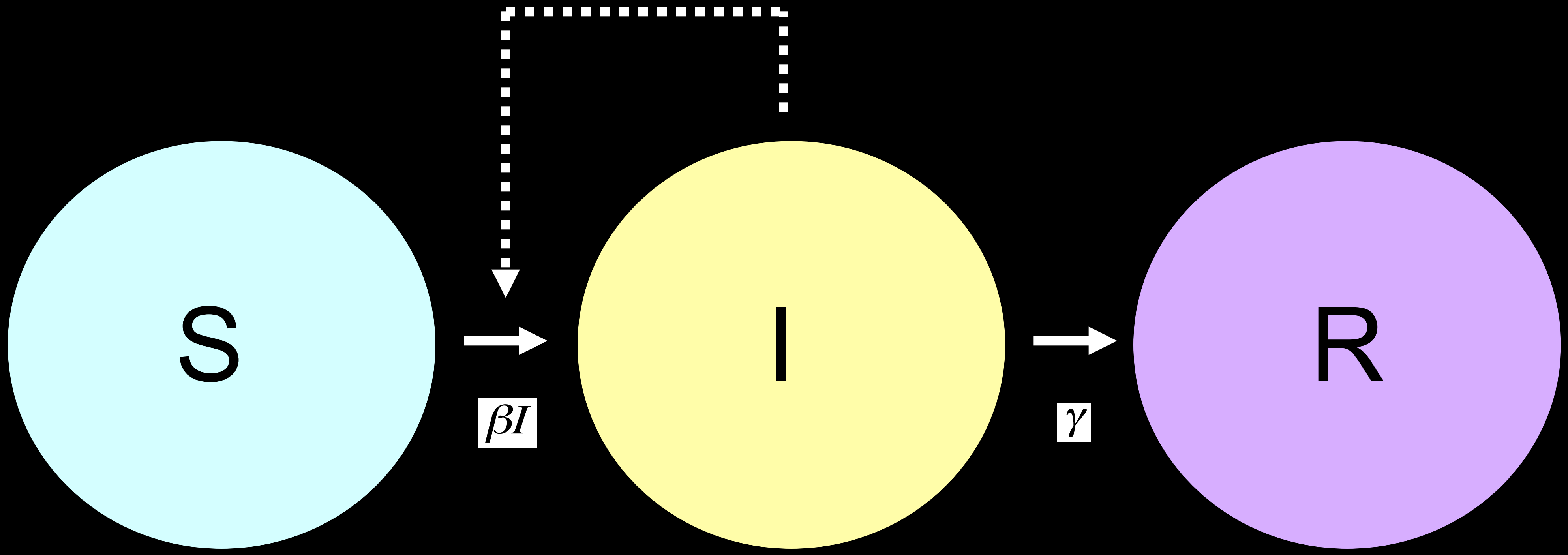

A simple disease model

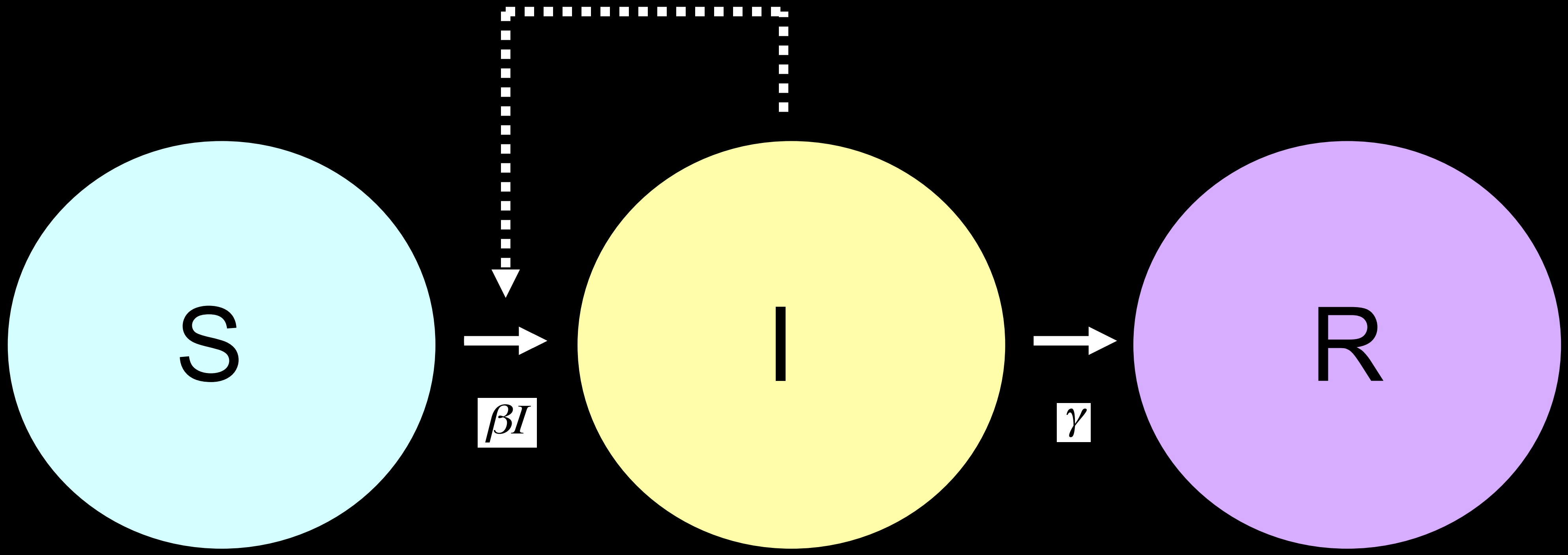

$$\frac{dS}{d\tau} = -\beta SI$$

$$\frac{dI}{d\tau} = \beta SI - \gamma I$$

$$\frac{dR}{d\tau} = \gamma I$$

A simple disease model

$N = S + I + R$

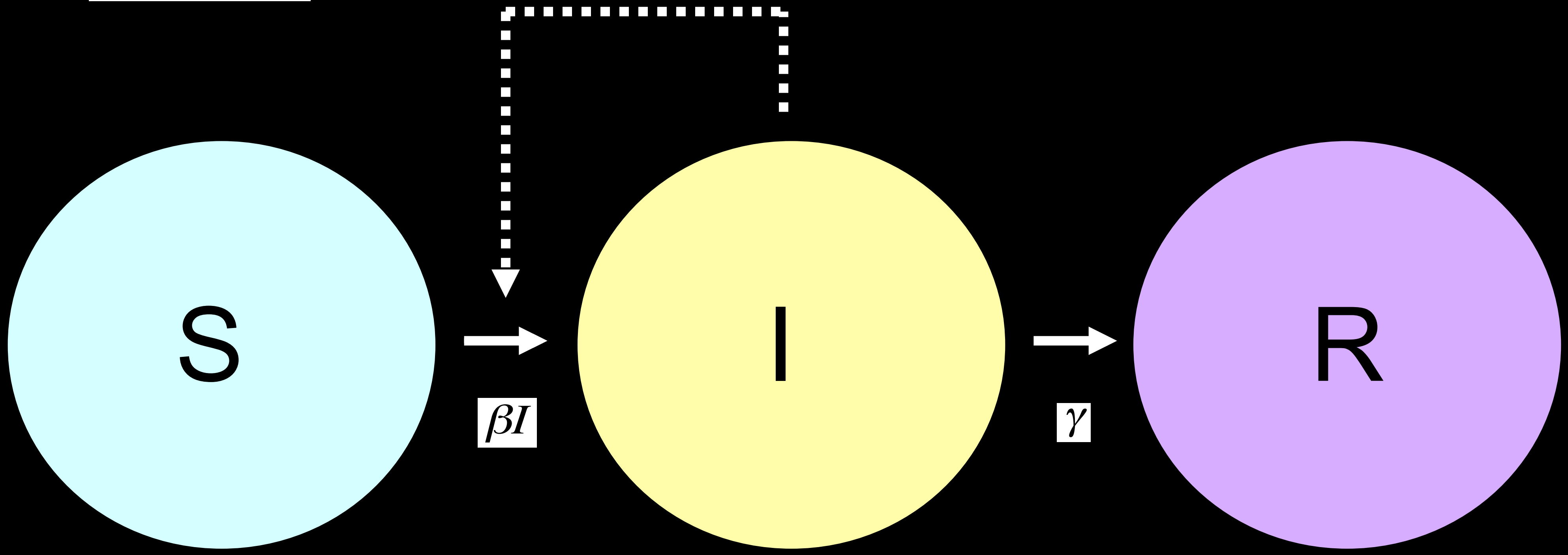

$$\frac{dS}{d\tau} = -\beta SI$$

$$\frac{dI}{d\tau} = \beta SI - \gamma I$$

$$\frac{dR}{d\tau} = \gamma I$$

A simple disease model

$N = S + I + R$

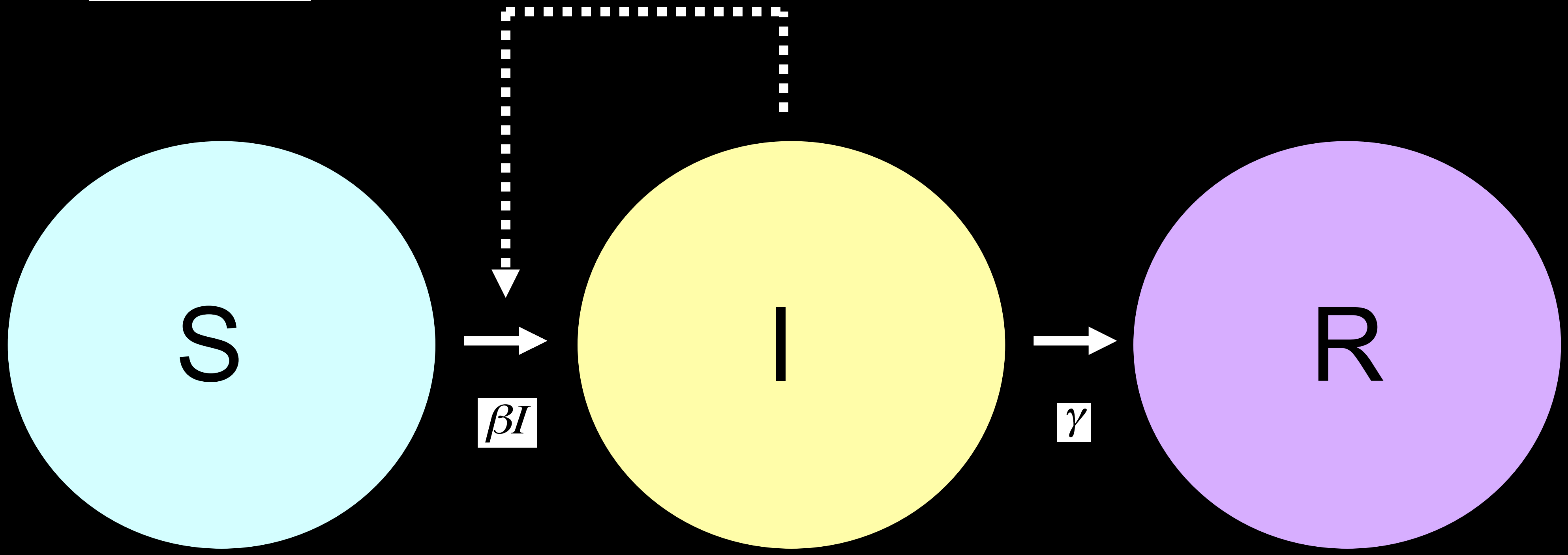

$$\frac{dS}{dt} = -\frac{\beta SI}{\gamma}$$

$$\frac{dI}{dt} = \frac{\beta SI}{\gamma} - I$$

$$\frac{dR}{dt} = I$$

A simple disease model

$N = S + I + R$

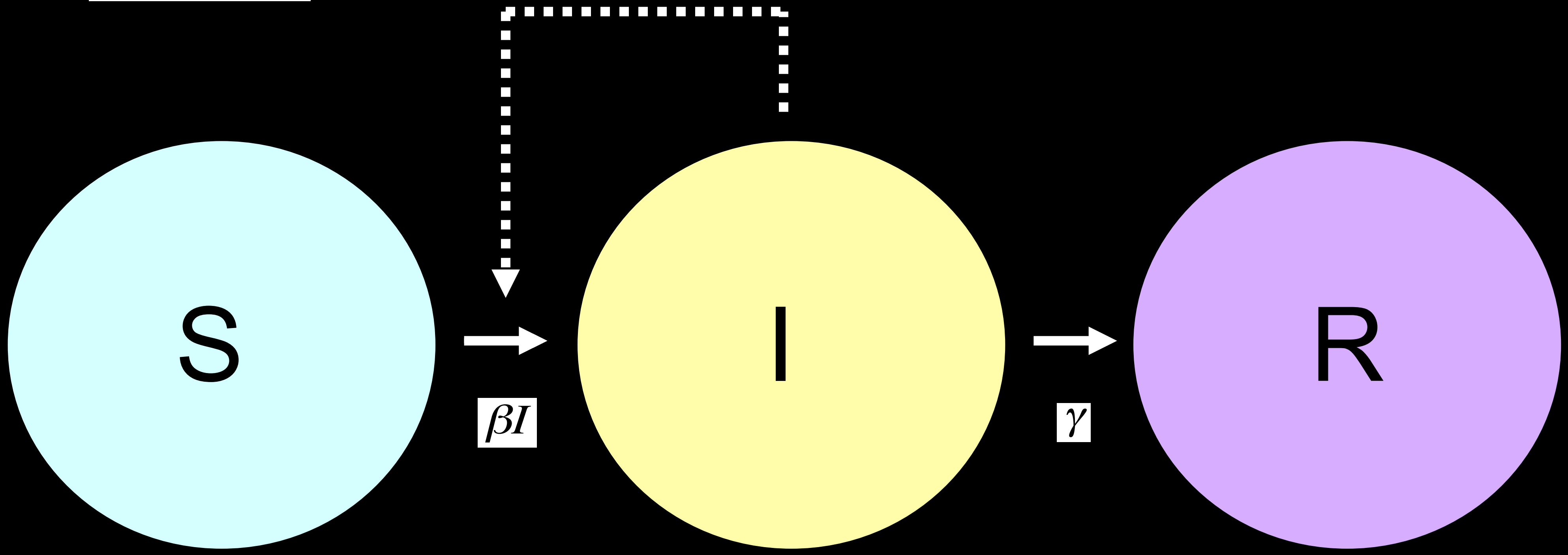

$$\frac{dS}{dt} = -\frac{\beta SI}{\gamma}$$

$$\frac{dI}{dt} = \frac{\beta SI}{\gamma} - I$$

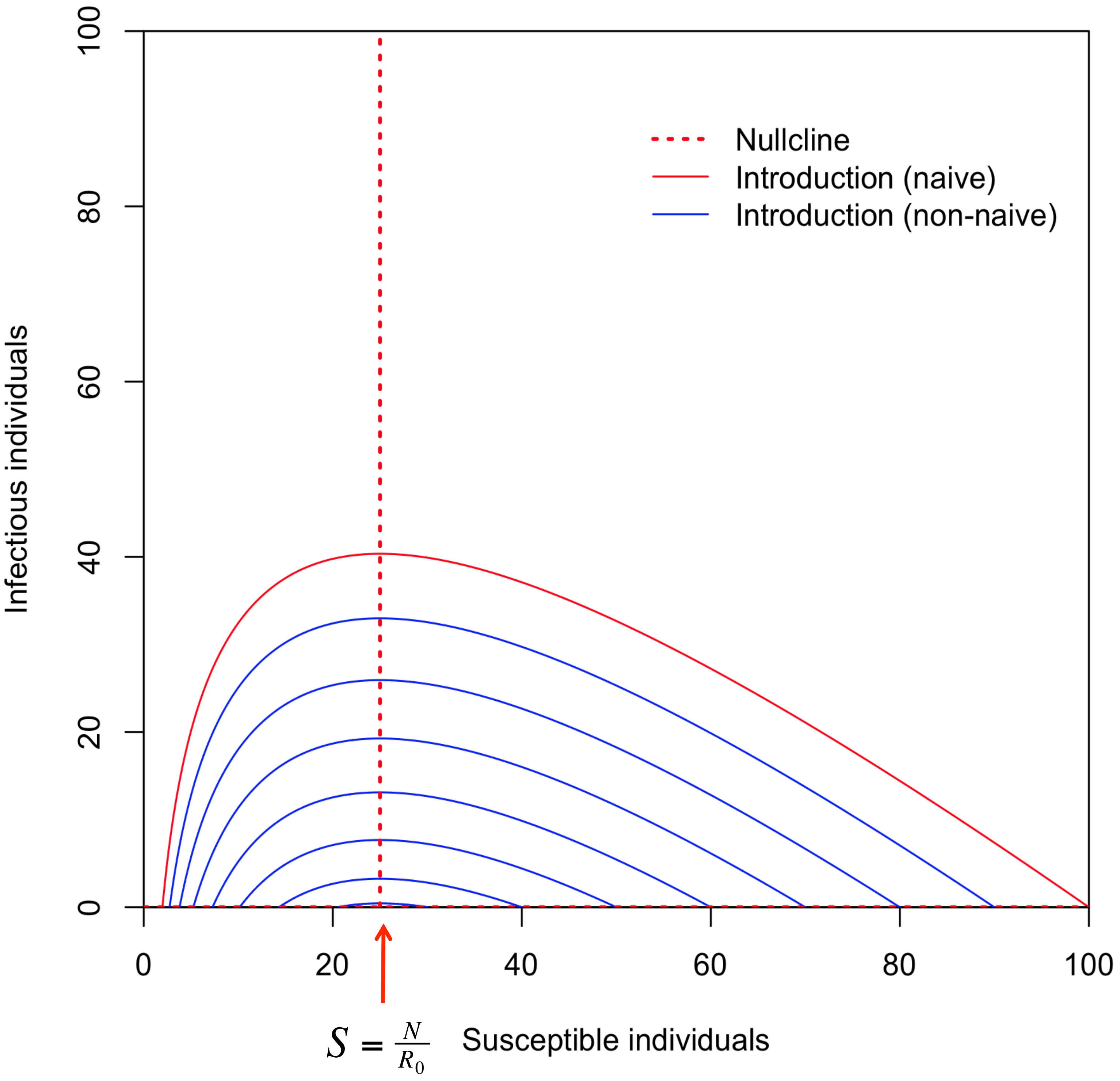

Nullcline
Introduction (naive)
Introduction (non-naive)
Infectious individuals
0
20
40
60
80
100
0
20
40
60
80
100
$S = \frac{N}{R_0}$
Susceptible individuals

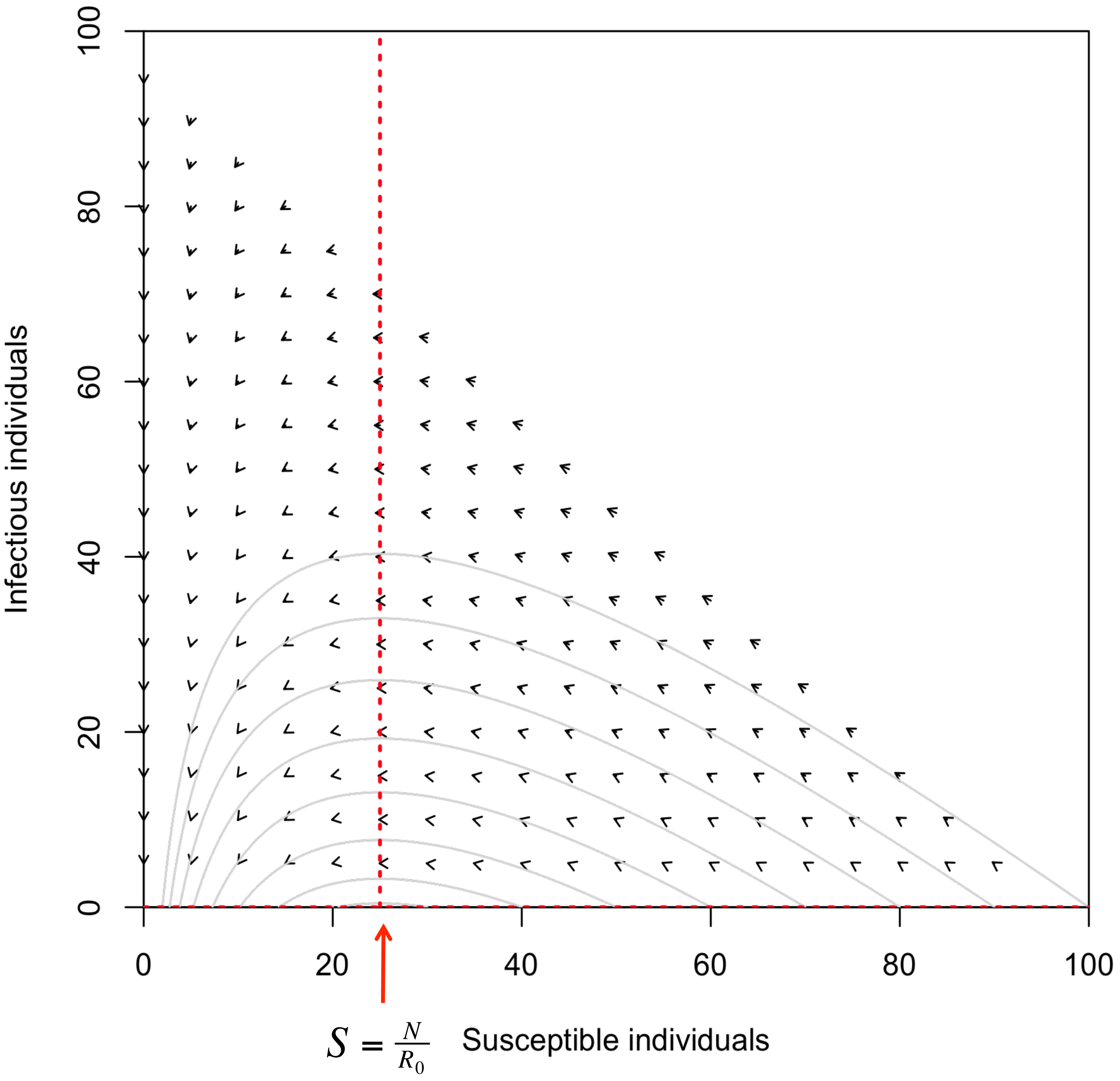

Infectious individuals
0
20
40
60
80
100
$S = \frac{N}{R_0}$
Susceptible individuals
