# Supplementary material for: How to Make Epidemiological Training Infectious
Source: PLoS Biol. 2012 Apr 3;10(4):e1001295. doi: 10.1371/journal.pbio.1001295 (PMC3317897; doi:10.1371/journal.pbio.1001295)
Supplement: Figure S2 — Lecture slide 2: introduction to dynamics of vector-borne diseases. (PDF) [file pbio.1001295.s009.pdf]

Dynamics of vector-borne pathogens

Dr. Juliet Pulliam
RAPIDD Program
Division of International Epidemiology
Fogarty International Center
National Institutes of Health (USA)

Topics in Biomedical Sciences
BSc Honours Course in Biomathematics
African Institute for the Mathematical Sciences
Muizenberg, South Africa
20 May 2010

Infectious diseases

Transmission

Mode of transmission

<u>Direct transmission</u>

Direct contact

Droplet spread

<u>Indirect transmission</u>

Airborne

Vehicle-borne (fomites)

Vector-borne (mechanical or biological)

Portal of entry

Portal of exit

Infectious diseases

Transmission

Mode of transmission

Direct transmission

Direct contact

Droplet spread

Indirect transmission

Airborne

Vehicle-borne (fomites)

Vector-borne (mechanical or biological) →

- Mosquitoes
- Ticks
- Sandflies
- Tsetse flies
- Reduviid bugs

Portal of entry

Portal of exit

Vector-borne pathogens

“ ”

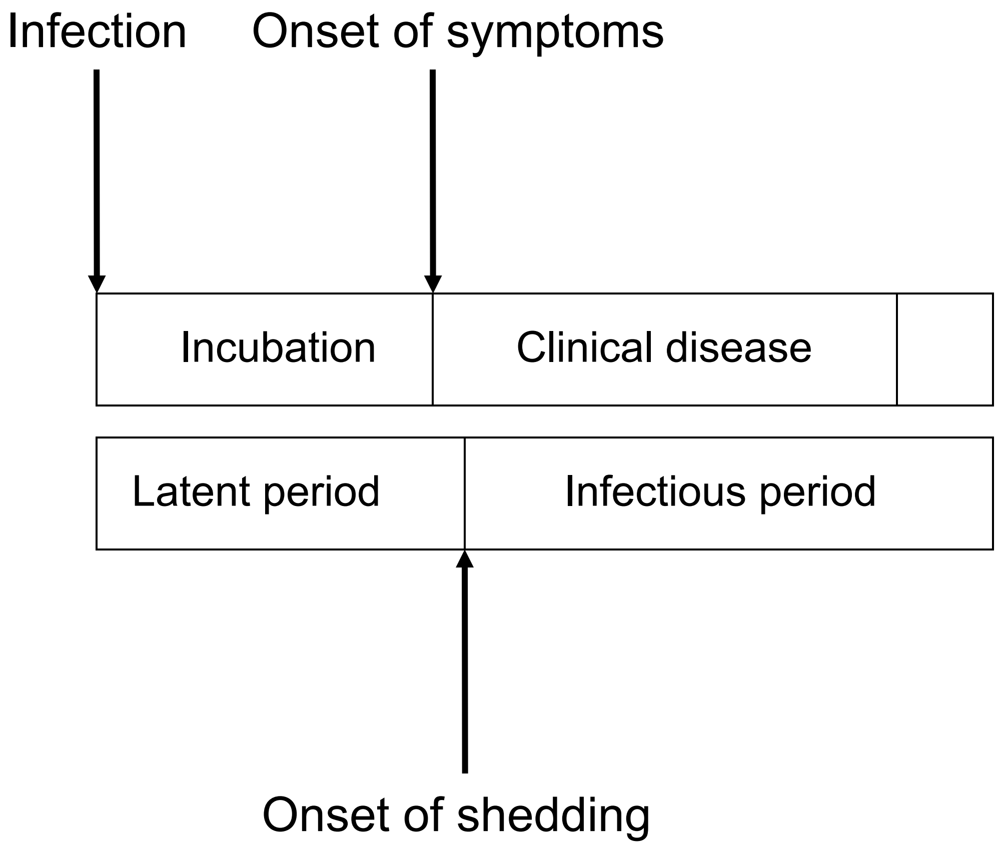

Vector-borne pathogens

“ ”

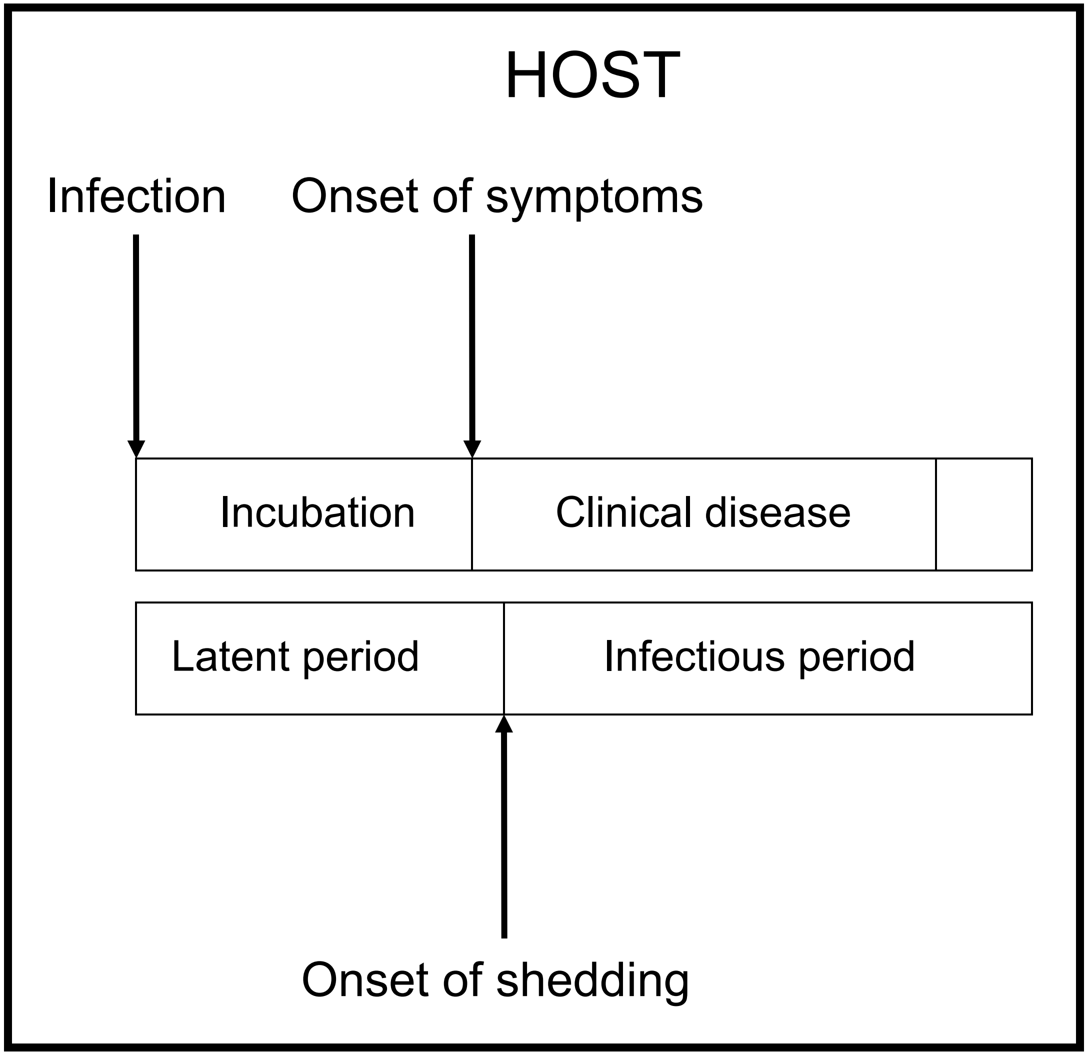

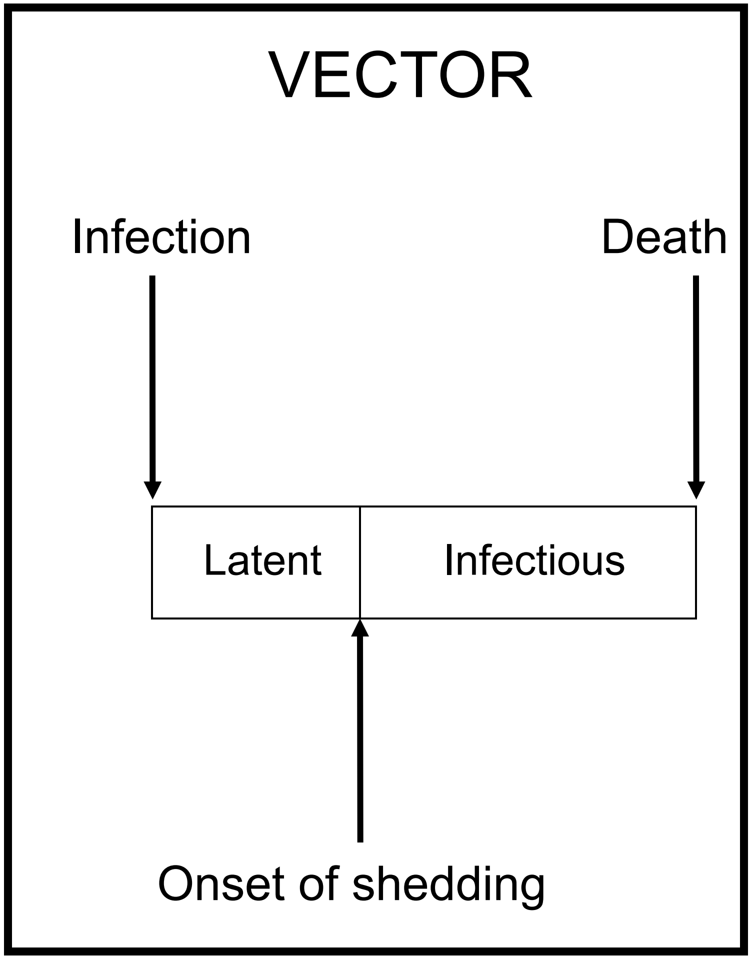

Vector-borne pathogens

“ ”

Often acute:

timecourse of infection <<
normal lifespan of host

BUT

timecourse of infection ~
normal lifespan of vector

Sometimes immunizing:

infection may stimulate antibody production, preventing future infection…

or may not…

or somewhere in between

Vector-borne pathogens

Examples

Mosquitoes

Anopheles spp., malaria vectors

Culex spp., West Nile vectors

Other biting flies

*Phlebotomus papatas*i, *Leishmania* vector

Glossina spp., African trypanosomiasis vectors

True bugs

Triatoma infestans, Chagas vector

Ticks

Amblyomma spp., heartwater vectors

Vector-borne pathogens

A not so simple view of the world

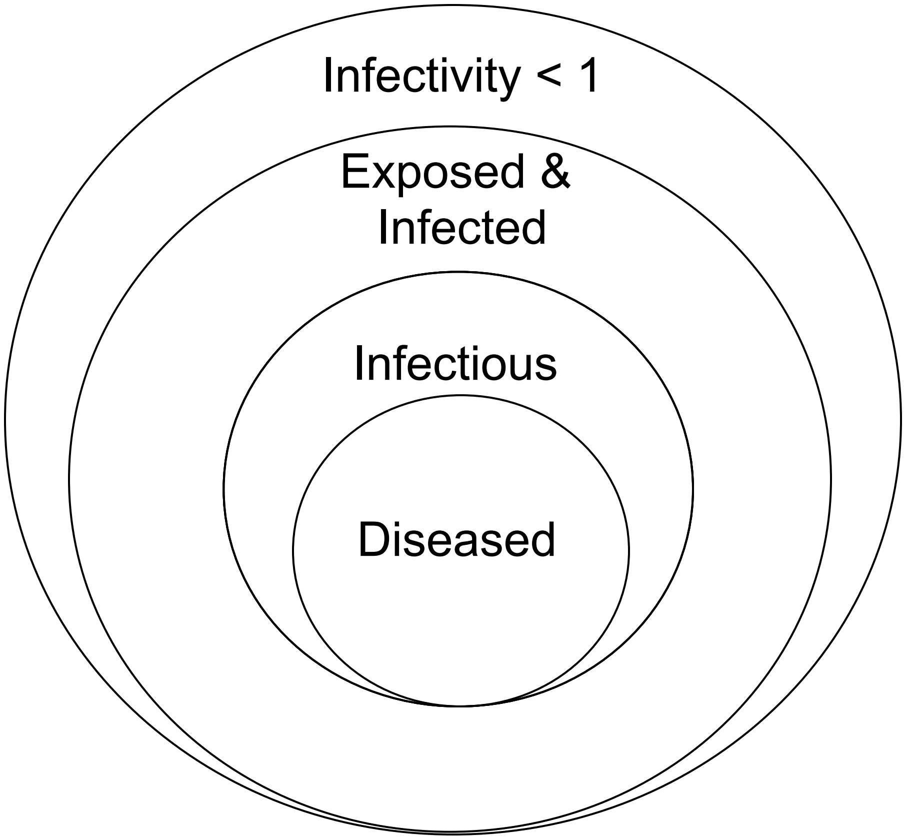

HOST

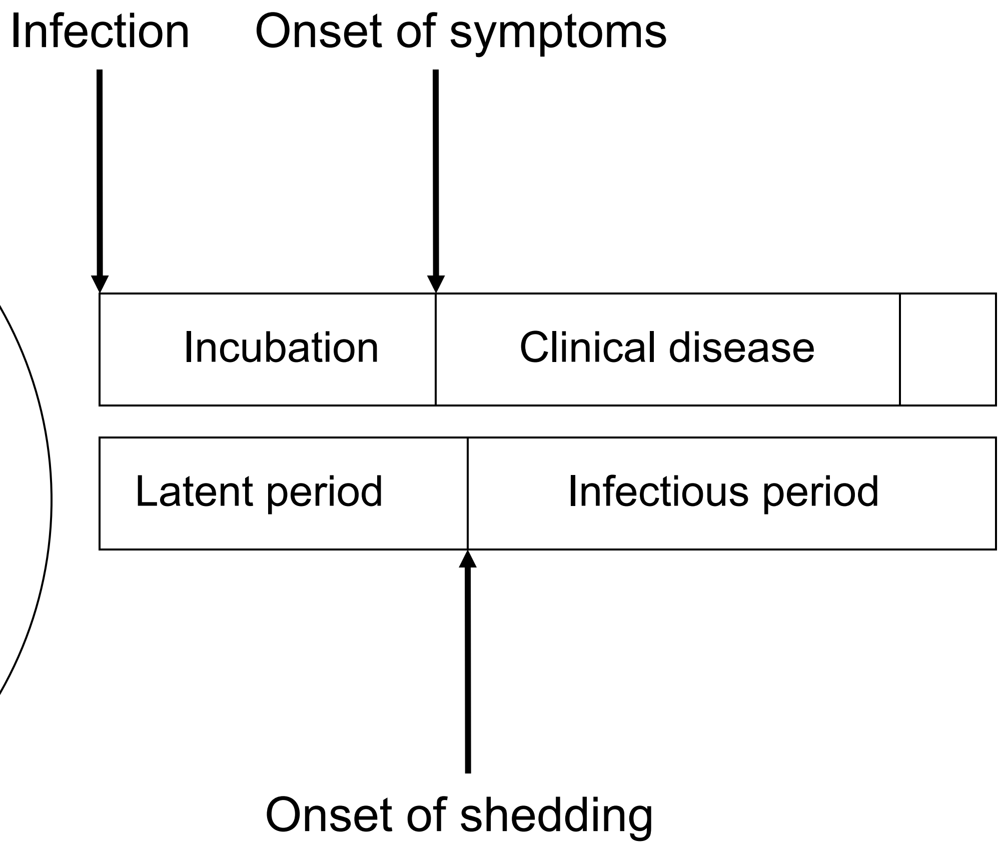

Vector-borne pathogens

not so

A‸simple view of the world

Don t worry about
symptoms and disease!

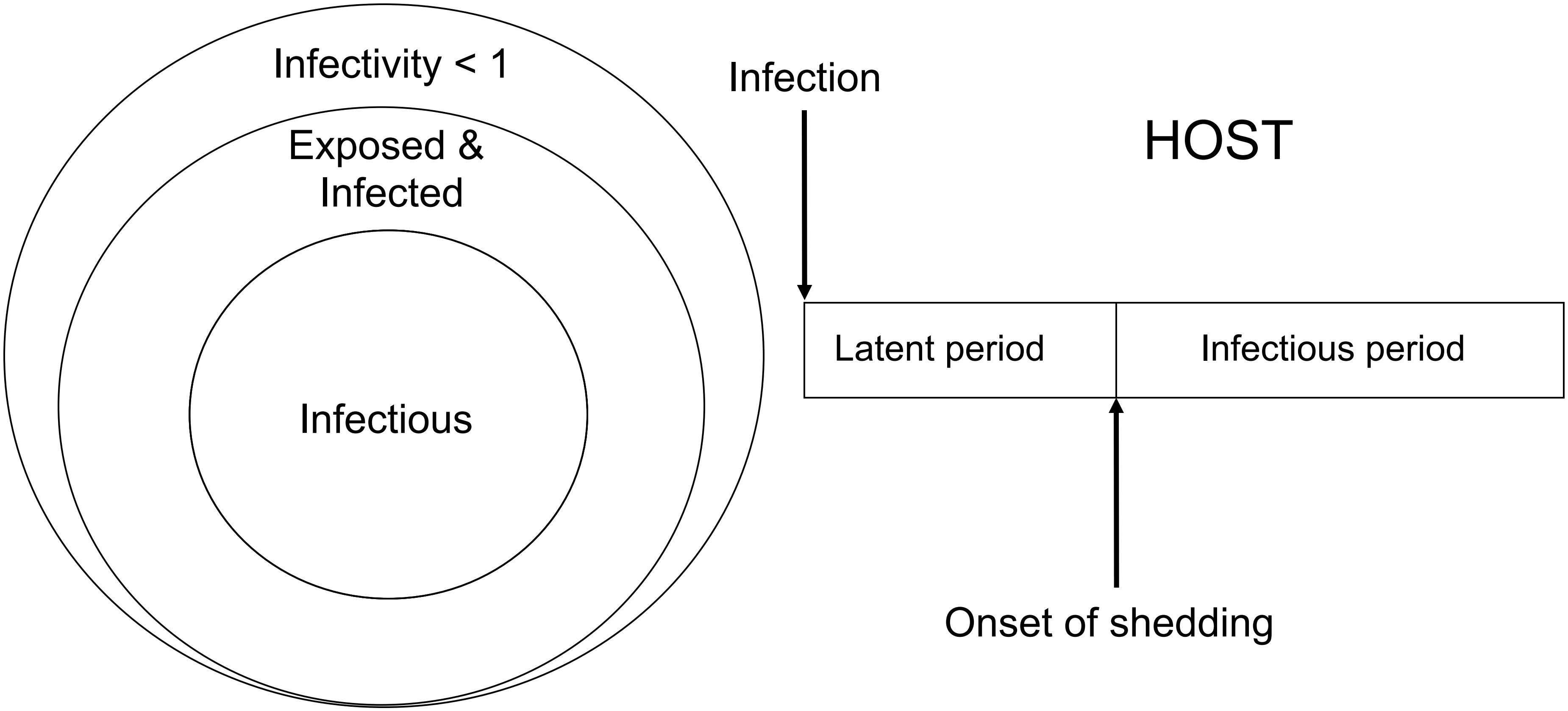

Vector-borne pathogens

A not so simple view of the world

β_H = infectivity to humans x per capita (vector) biting rate

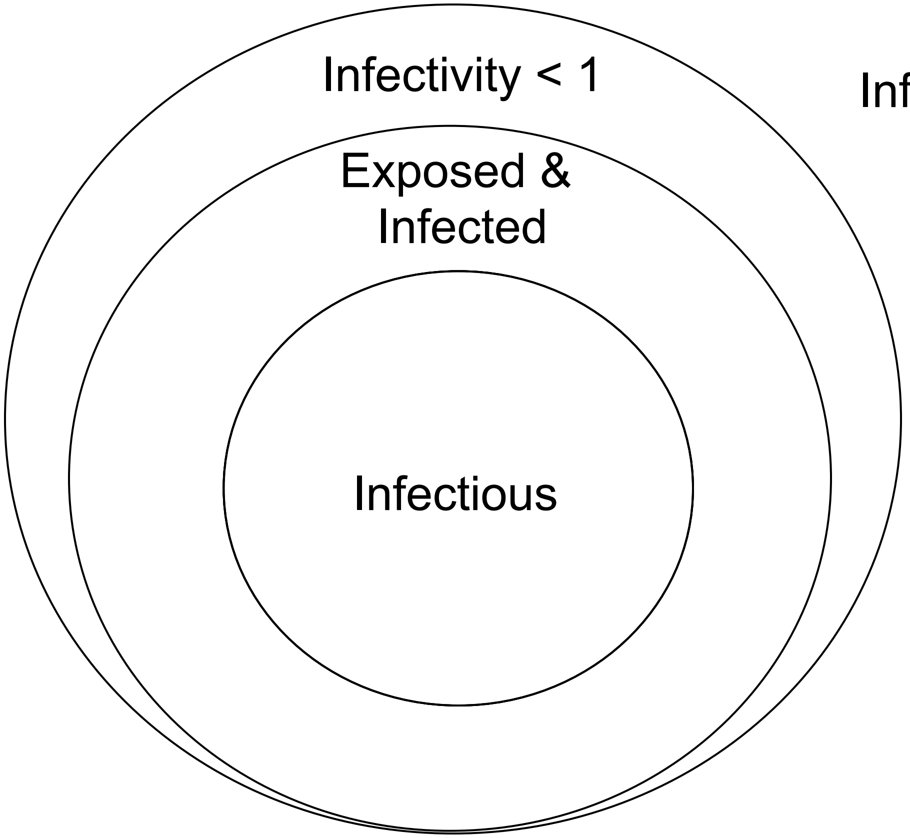

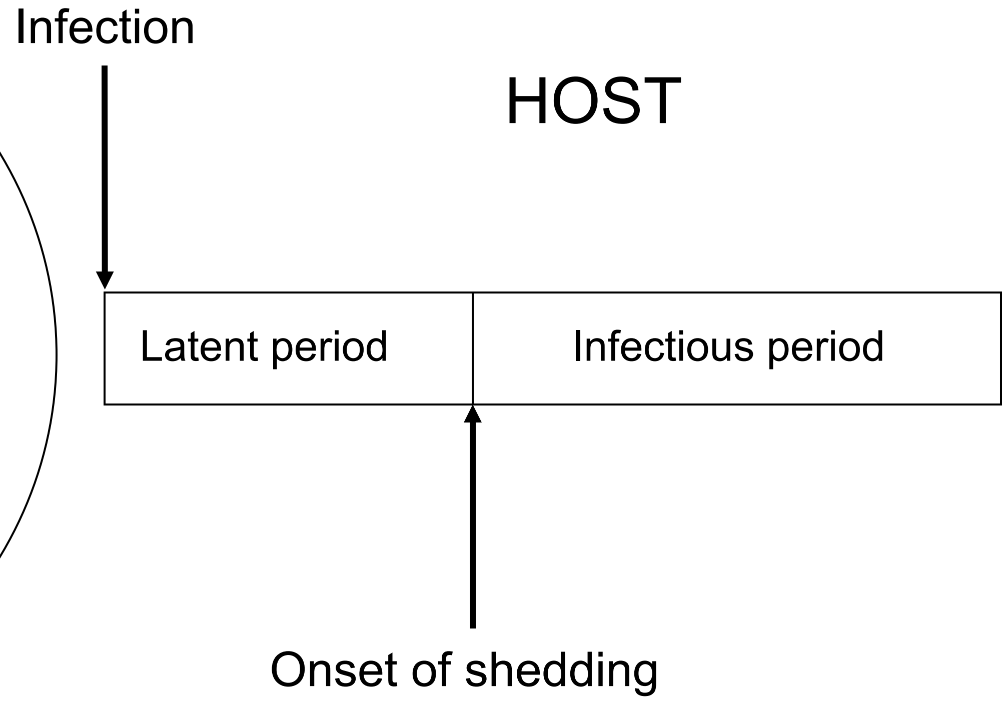

Vector-borne pathogens

A not so simple view of the world

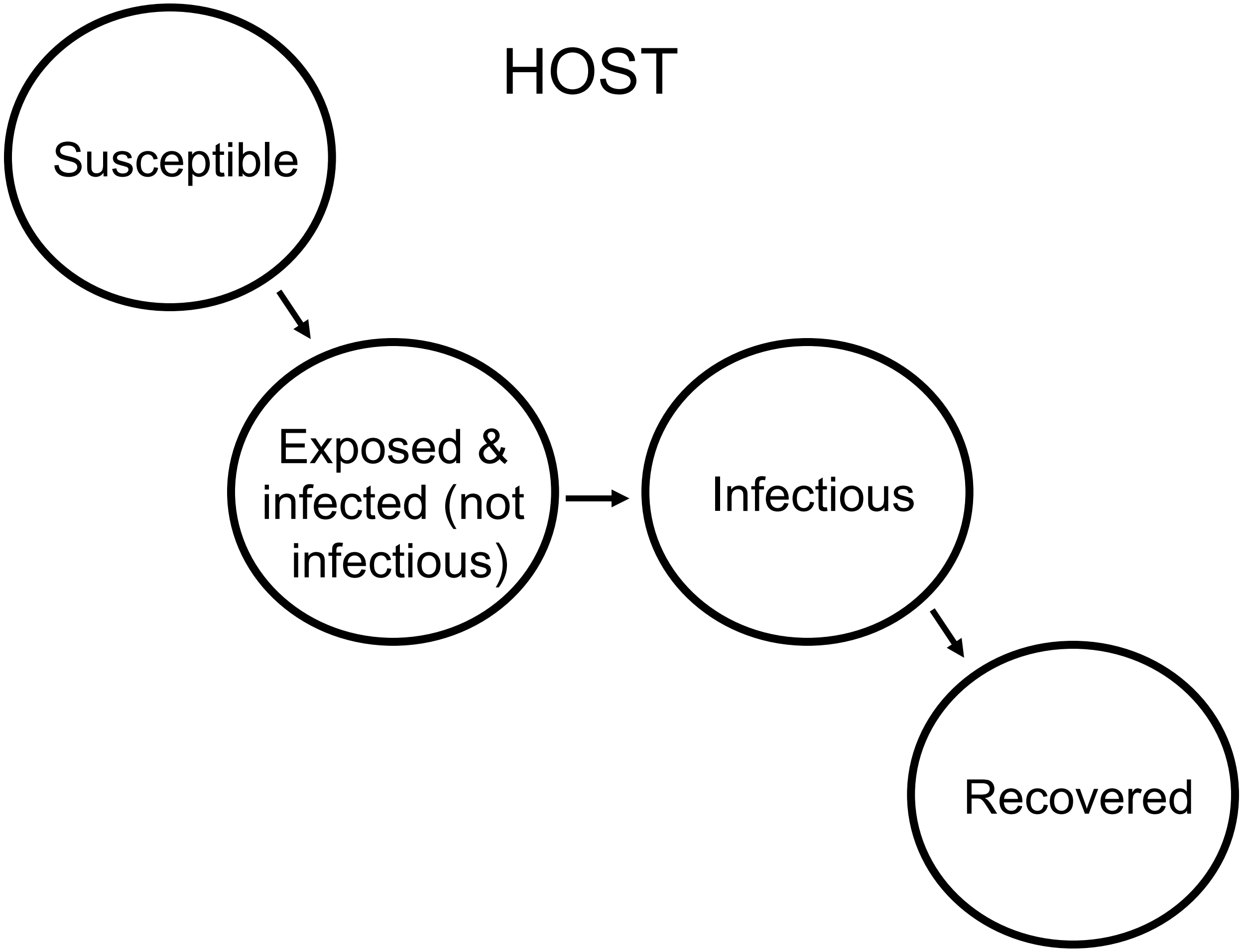

Vector-borne pathogens

not so

A‸simple view of the world

β_V = infectivity to vectors x per capita (vector) biting rate

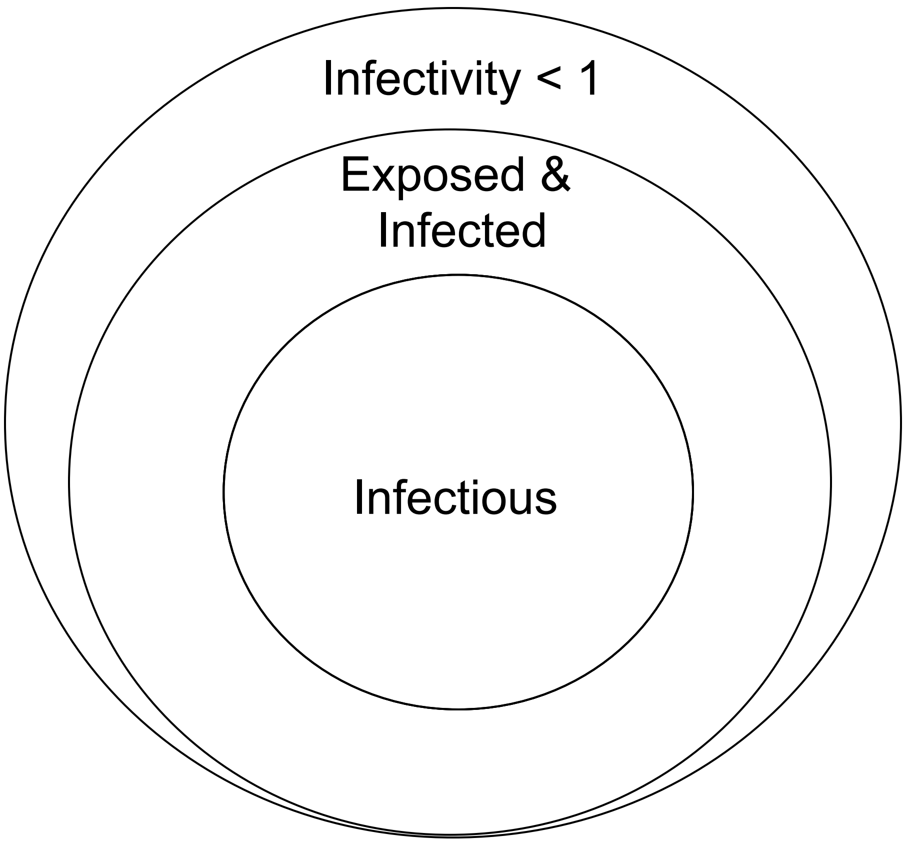

VECTOR

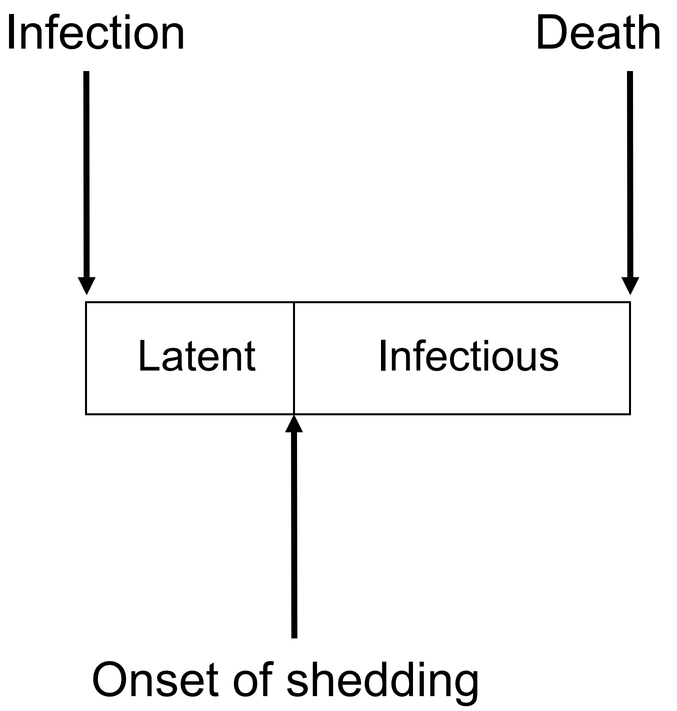

Vector-borne pathogens

A simple view of the world

not so (inserted with ^ between "A" and "simple")

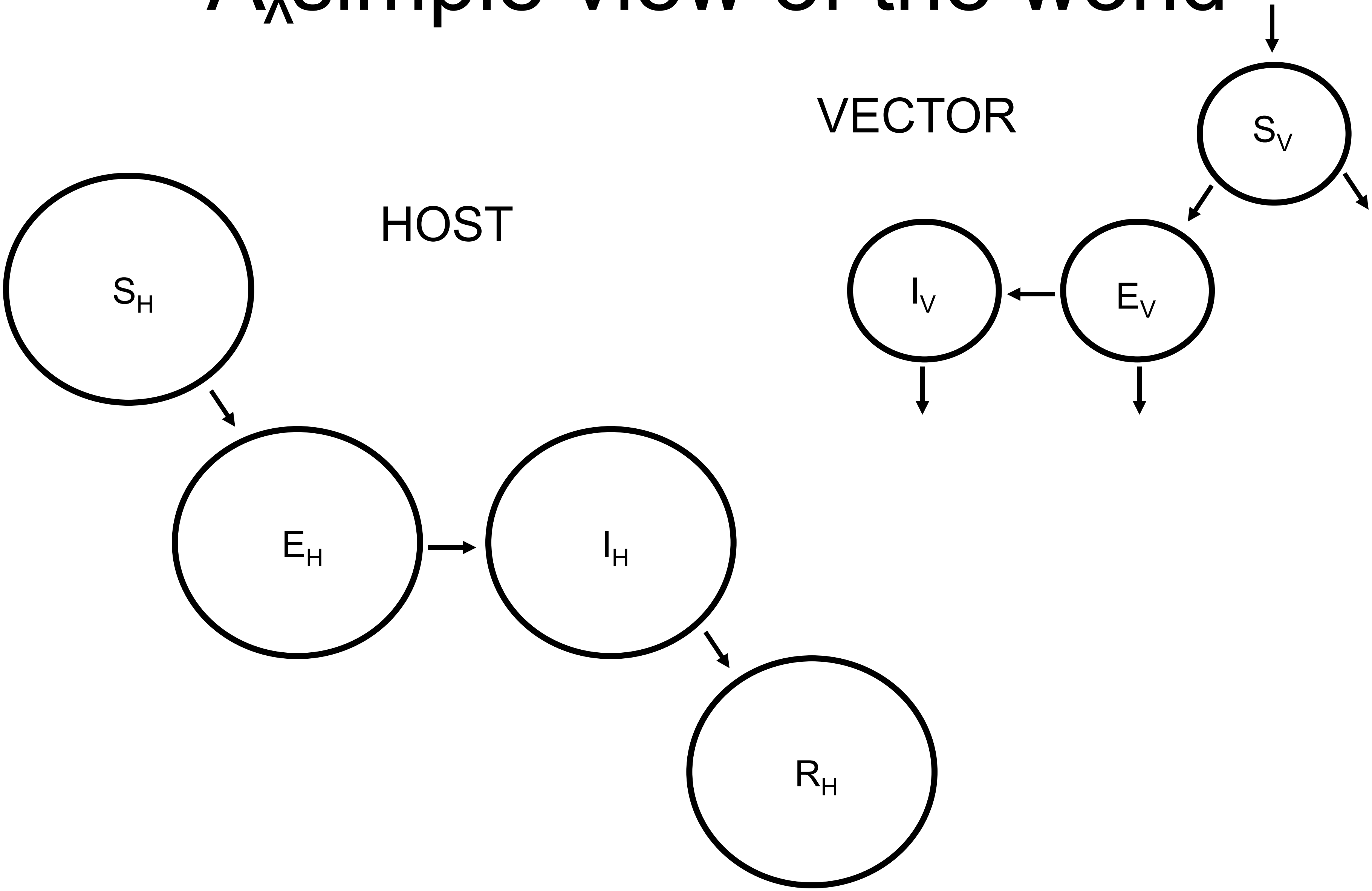

Vector-borne pathogens

A [not so] simple view of the world

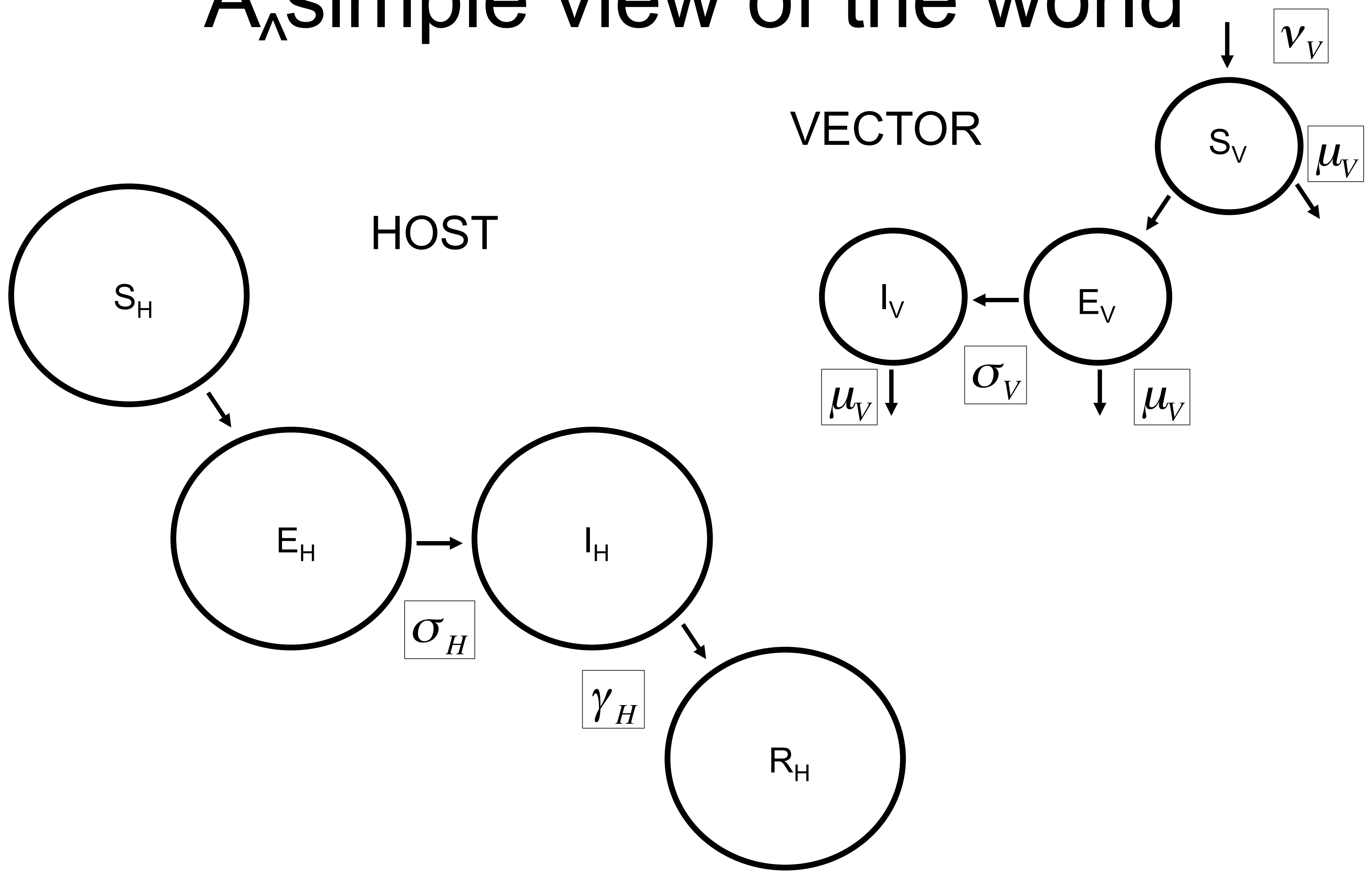

Vector-borne pathogens

A not so simple view of the world

ν_V birth rate

μ_V per capita mortality rate

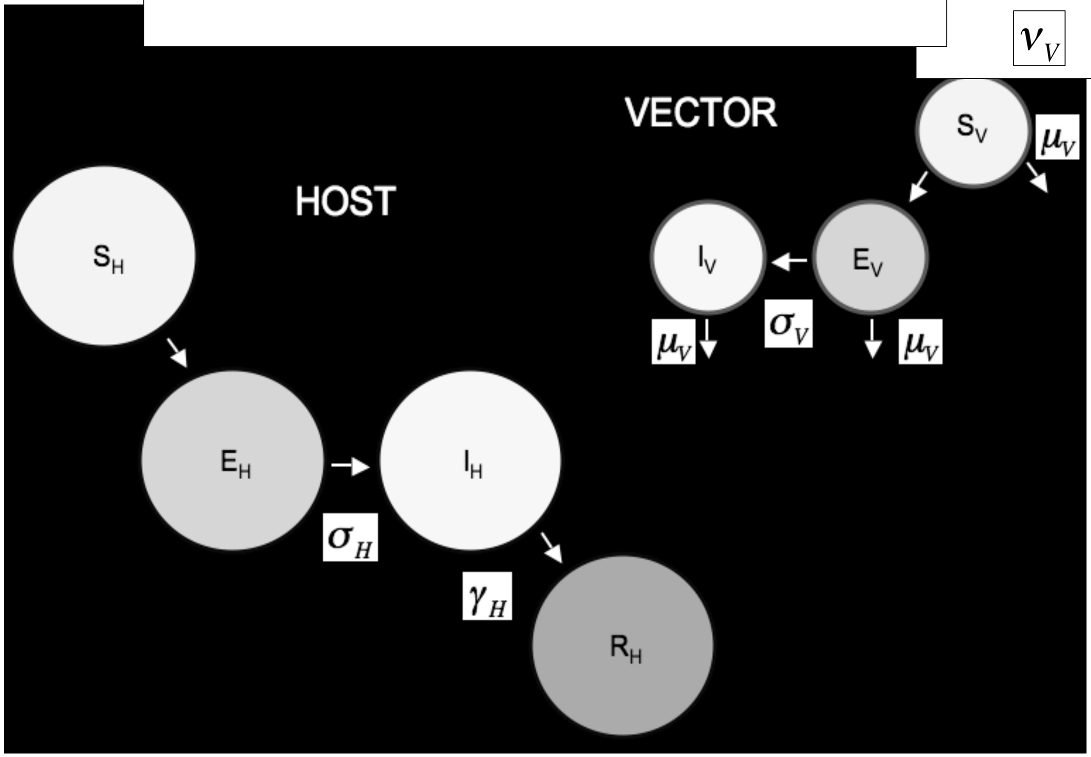

ν_V per capita birth rate

μ_V per capita mortality rate

σ_H, σ_V 1/latent period

γ_H 1/infectious period

Vector-borne pathogens

A not so simple view of the world

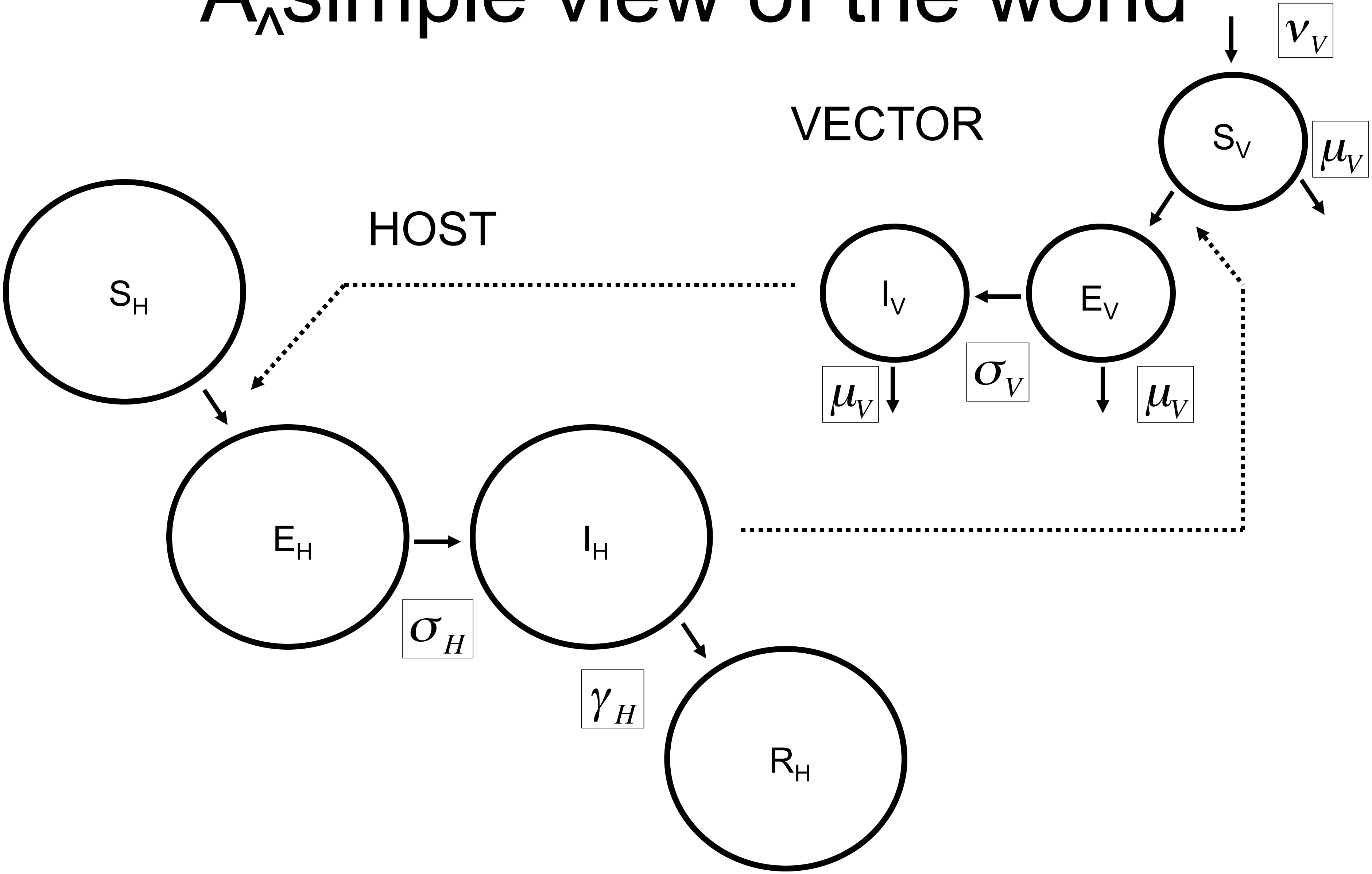

Vector-borne pathogens

A not so simple view of the world

β = infectivity x per capita contact rate

β = infectivity x per capita (vector) biting rate

infectivity = proportion of susceptible individuals that become infected, given exposure

per capita (vector) biting rate = bites by one individual vector per time unit

HOST

exposure = bite by I_V

VECTOR

exposure = bite on I_H

Vector-borne pathogens

A not so simple view of the world

HOST

β = infectivity x per capita contact rate

β = infectivity x per capita biting rate

infectivity = proportion of susceptible individuals that become infected, given exposure

per capita (vector) biting rate = bites by one individual vector per unit time

exposure = bite by I_V

infectivity to host = host infections produced per bite by I_V on S_H

β_H = bites (potentially infectious to host) by one individual vector per unit time

$\beta_H I_V$ = bites (potentially infectious to host) per unit time

$\beta_H I_V/N_H$ = bites (potentially infectious to host) per host per unit time

$\beta_H S_H I_V/N_H$ = infectious bites per unit time

Vector-borne pathogens

A not so simple view of the world

VECTOR

β = infectivity x per capita contact rate

β = infectivity x per capita biting rate

infectivity = proportion of susceptible individuals that become infected, given exposure

per capita (vector) biting rate = bites by one individual vector per unit time

exposure = bites on I_H

infectivity to vector = vector infections produced per bite by S_V on I_H

β_V = bites (potentially infectious to vector) by one individual vector per unit time

$\beta_V S_V$ = bites (potentially infectious to vector) per unit time

$\beta_V S_V/N_H$ = bites (potentially infectious to vector) per host per unit time

$\beta_V S_V I_H/N_H$ = infectious bites per unit time

Vector-borne pathogens

A not so simple view of the world

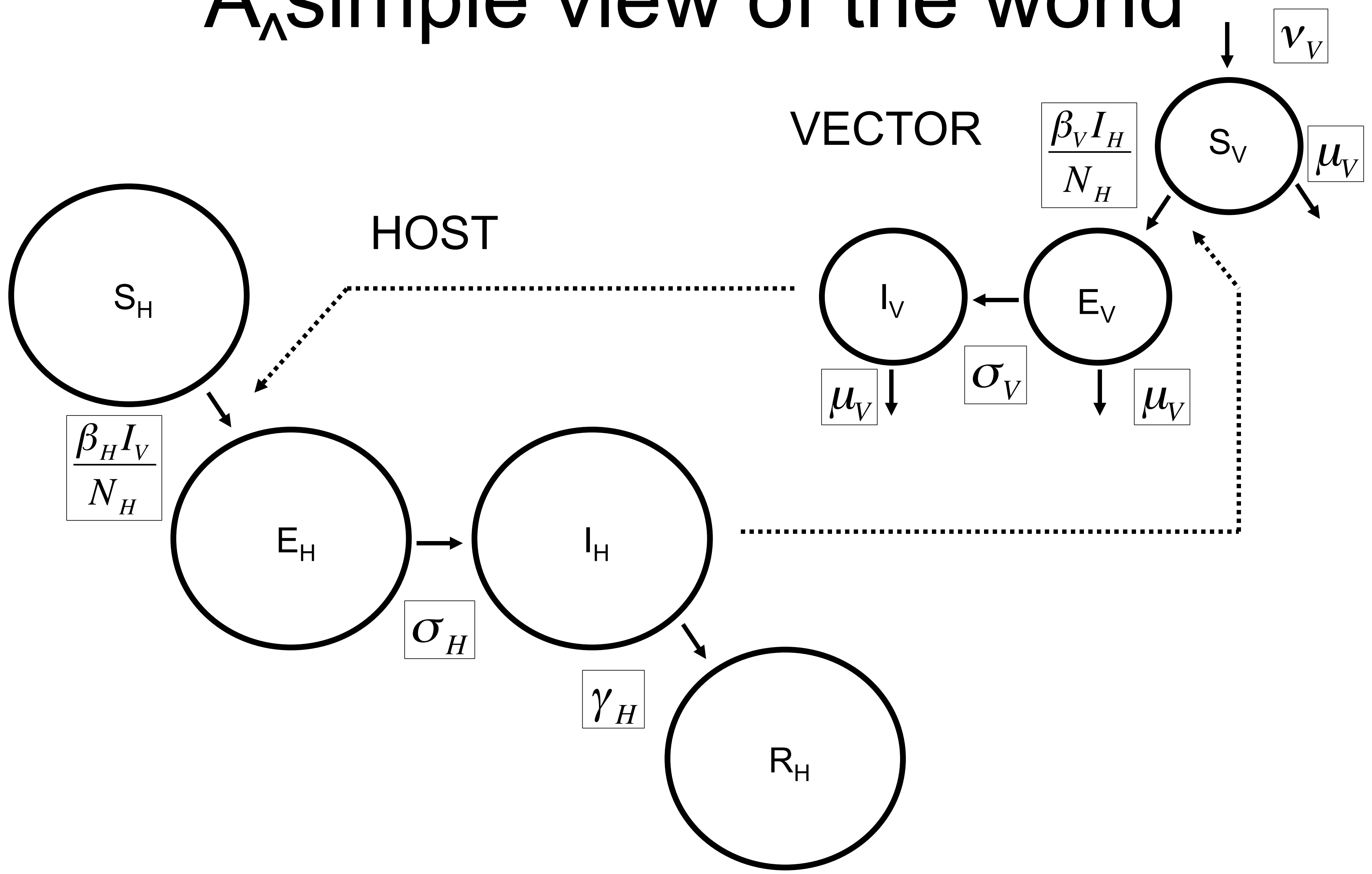

Vector-borne pathogens

A not so simple view of the world

HOST

$$\frac{dS_H}{dt} = \frac{-\beta S_H I_V}{N_H}$$

$$\frac{dE_H}{dt} = \frac{\beta S_H I_V}{N_H} - \sigma E_H$$

$$\frac{dI_H}{dt} = \sigma E_H - \gamma I_H$$

$$\frac{dR_H}{dt} = \gamma I_H$$

VECTOR

$$\frac{dS_V}{dt} = \nu_V - \mu_V S_V - \frac{\beta_V S_V I_H}{N_H}$$

$$\frac{dE_V}{dt} = \frac{\beta_V S_V I_H}{N_H} - (\mu_V + \sigma_V) E_V$$

$$\frac{dI_V}{dt} = \sigma_V E_V - (\mu_V + \gamma_V) I_V$$

Vector-borne pathogens

A not so simple view of the world

$$R_0 = ?$$

HOST

$$\frac{dS_H}{dt} = \frac{-\beta S_H I_V}{N_H}$$

$$\frac{dE_H}{dt} = \frac{\beta S_H I_V}{N_H} - \sigma E_H$$

$$\frac{dI_H}{dt} = \sigma E_H - \gamma I_H$$

$$\frac{dR_H}{dt} = \gamma I_H$$

VECTOR

$$\frac{dS_V}{dt} = \nu_V - \mu_V S_V - \frac{\beta_V S_V I_H}{N_H}$$

$$\frac{dE_V}{dt} = \frac{\beta_V S_V I_H}{N_H} - (\mu_V + \sigma_V) E_V$$

$$\frac{dI_V}{dt} = \sigma_V E_V - (\mu_V + \gamma_V) I_V$$

Vector-borne pathogens

A simple method for complex models

$$R_0 = \rho\left(FV^{-1}\right)$$

FV^{-1} = is the next generation matrix

For all compartments x_i containing infected individuals (ie, E_H, I_H, E_V, I_V), the time derivative can be rewritten as

$$\frac{dx_i}{dt} = f_i(x) = \mathcal{F}_i(x) - \mathcal{V}_i^-(x) + \mathcal{V}_i^+(x)$$

where

$\mathcal{F}_i(x)$ = the rate of appearance of new infections in compartment x_i

$\mathcal{V}_i^-(x)$ = the rate of transfer out of compartment x_i

$\mathcal{V}_i^+(x)$ = the rate of transfer of individuals into compartment x_i, other than new infections

Vector-borne pathogens

A simple method for complex models

$$R_0 = \rho\left(FV^{-1}\right)$$

FV^{-1} = is the next generation matrix

F and V are then the square matrices defined by

$$F = \left[\frac{\partial \mathcal{F}_i(x_0)}{\partial x_j}\right] \quad \text{and} \quad V = \left[\frac{\partial \mathcal{V}_i(x_0)}{\partial x_j}\right]$$

where

$$\mathcal{V}_i = \mathcal{V}_i^- - \mathcal{V}_i^+(x)$$

Vector-borne pathogens

A not so simple view of the world

$$\frac{dS_H}{dt} = \frac{-\beta S_H I_V}{N_H}$$

$$\frac{dE_H}{dt} = \frac{\beta S_H I_V}{N_H} - \sigma E_H$$

$$\frac{dI_H}{dt} = \sigma E_H - \gamma I_H$$

$$\frac{dR_H}{dt} = \gamma I_H$$

For our system, we have

$$x_1 = E_H, x_2 = I_H, x_3 = E_V, x_4 = I_V$$

$$\frac{dS_V}{dt} = \nu_V - \mu_V S_V - \frac{\beta_V S_V I_H}{N_H}$$

$$\frac{dE_V}{dt} = \frac{\beta_V S_V I_H}{N_H} - (\mu_V + \sigma_V) E_V$$

$$\frac{dI_V}{dt} = \sigma_V E_V - (\mu_V + \gamma_V) I_V$$

Vector-borne pathogens

A not so simple view of the world

$$\frac{dS_H}{dt} = \frac{-\beta S_H I_V}{N_H}$$

$$\frac{dE_H}{dt} = \frac{\beta S_H I_V}{N_H} - \sigma E_H$$

$$\frac{dI_H}{dt} = \sigma E_H - \gamma I_H$$

$$\frac{dR_H}{dt} = \gamma I_H$$

$$\frac{dS_V}{dt} = \nu_V - \mu_V S_V - \frac{\beta_V S_V I_H}{N_H}$$

$$\frac{dE_V}{dt} = \frac{\beta_V S_V I_H}{N_H} - (\mu_V + \sigma_V) E_V$$

$$\frac{dI_V}{dt} = \sigma_V E_V - (\mu_V + \gamma_V) I_V$$

For our system, we have

$$x_1 = E_H, x_2 = I_H, x_3 = E_V, x_4 = I_V$$

and we find

$$F = \begin{bmatrix} 0 & 0 & 0 & \beta_H \\ 0 & 0 & 0 & 0 \\ 0 & \beta_V & 0 & 0 \\ 0 & 0 & 0 & 0 \end{bmatrix}$$

$$V = \begin{bmatrix} \sigma_H & 0 & 0 & 0 \\ -\sigma_H & \gamma_H & 0 & 0 \\ 0 & 0 & \mu_V + \sigma_V & 0 \\ 0 & 0 & -\sigma_V & \mu_V + \sigma_V \end{bmatrix}$$

Vector-borne pathogens

A not so simple view of the world

$$\frac{dS_H}{dt} = \frac{-\beta S_H I_V}{N_H}$$

$$\frac{dE_H}{dt} = \frac{\beta S_H I_V}{N_H} - \sigma E_H$$

$$\frac{dI_H}{dt} = \sigma E_H - \gamma I_H$$

$$\frac{dR_H}{dt} = \gamma I_H$$

$$\frac{dS_V}{dt} = \nu_V - \mu_V S_V - \frac{\beta_V S_V I_H}{N_H}$$

$$\frac{dE_V}{dt} = \frac{\beta_V S_V I_H}{N_H} - (\mu_V + \sigma_V) E_V$$

$$\frac{dI_V}{dt} = \sigma_V E_V - (\mu_V + \gamma_V) I_V$$

For our system, we have

$$x_1 = E_H, x_2 = I_H, x_3 = E_V, x_4 = I_V$$

which gives

$$FV^{-1} = \begin{bmatrix} 0 & 0 & \frac{\beta_H \sigma_V}{(\mu_V + \sigma_V)^2} & \frac{\beta_H}{\mu_V + \sigma_V} \\ 0 & 0 & 0 & 0 \\ \frac{\beta_V}{\gamma_H} & \frac{\beta_V}{\gamma_H} & 0 & 0 \\ 0 & 0 & 0 & 0 \end{bmatrix}$$

Vector-borne pathogens

A not so simple view of the world

$$\frac{dS_H}{dt} = \frac{-\beta S_H I_V}{N_H}$$

$$\frac{dE_H}{dt} = \frac{\beta S_H I_V}{N_H} - \sigma E_H$$

$$\frac{dI_H}{dt} = \sigma E_H - \gamma I_H$$

$$\frac{dR_H}{dt} = \gamma I_H$$

$$\frac{dS_V}{dt} = \nu_V - \mu_V S_V - \frac{\beta_V S_V I_H}{N_H}$$

$$\frac{dE_V}{dt} = \frac{\beta_V S_V I_H}{N_H} - (\mu_V + \sigma_V) E_V$$

$$\frac{dI_V}{dt} = \sigma_V E_V - (\mu_V + \gamma_V) I_V$$

For our system, we have

$$x_1 = E_H, x_2 = I_H, x_3 = E_V, x_4 = I_V$$

"next generation matrix"

$$FV^{-1} = \begin{bmatrix} 0 & 0 & R_0^{(E_V \to E_H)} & R_0^{(I_V \to E_H)} \\ 0 & 0 & 0 & 0 \\ R_0^{(E_H \to E_V)} & R_0^{(I_H \to E_V)} & 0 & 0 \\ 0 & 0 & 0 & 0 \end{bmatrix}$$

Vector-borne pathogens

not so

A‸simple view of the world

$$\frac{dS_H}{dt} = \frac{-\beta S_H I_V}{N_H}$$

$$\frac{dE_H}{dt} = \frac{\beta S_H I_V}{N_H} - \sigma E_H$$

$$\frac{dI_H}{dt} = \sigma E_H - \gamma I_H$$

$$\frac{dR_H}{dt} = \gamma I_H$$

$$\frac{dS_V}{dt} = \nu_V - \mu_V S_V - \frac{\beta_V S_V I_H}{N_H}$$

$$\frac{dE_V}{dt} = \frac{\beta_V S_V I_H}{N_H} - (\mu_V + \sigma_V) E_V$$

$$\frac{dI_V}{dt} = \sigma_V E_V - (\mu_V + \gamma_V) I_V$$

For our system, we have

$$x_1 = E_H, x_2 = I_H, x_3 = E_V, x_4 = I_V$$

and

$$R_0 = \rho\left(FV^{-1}\right) = \sqrt{\frac{\beta_H \beta_V \sigma_H}{(\mu_V + \sigma_V)^2 \gamma_H}}$$

$$R_0^{\ 2} = \frac{\beta_H \beta_V \sigma_H}{(\mu_V + \sigma_V)^2 \gamma_H}$$
