# Supplementary material for: How to Make Epidemiological Training Infectious
Source: PLoS Biol. 2012 Apr 3;10(4):e1001295. doi: 10.1371/journal.pbio.1001295 (PMC3317897; doi:10.1371/journal.pbio.1001295)
Supplement: Figure S4 — Lecture slide 4: study design and analysis in epidemiology. (PDF) [file pbio.1001295.s011.pdf]

Study Design and Analysis in Epidemiology:

Where does modeling fit?

Meaningful Modeling of Epidemiologic Data, 2011
AIMS, Muizenberg, South Africa

Steve Bellan
MPH Epidemiology
PhD Candidate
Department of Environmental Science, Policy & Management
University of California at Berkeley

Defining Epidemiology

"The study of the distribution and determinants of health related states and events in populations, and the application of this study to control health problems."

John M Last
Dictionary of Epidemiology

Varieties of Infectious Disease Epidemiology

- Risk Factors & Intervention Epidemiology

    Risk Factor: A characteristic that is correlated with a measure of disease.

    - Often used synonymously with *covariate*.

    - Protective factors: Risk factors that are negatively associated with disease

Varieties of Infectious Disease Epidemiology

- Risk Factors & Intervention
- Outbreak
- Clinical
- Molecular & Genetic
- Surveillance

How does mathematical modeling fit?

- A subfield of epidemiology:

  Linking pattern with process across scales

BUT ALSO

- A set of methodologies to be used in any field of epidemiology

Importance of knowledge breadth

What do *Introductory Epidemiology* courses teach?

- Measures of Disease
- Measures of Effect (of a risk factor)
- Study Designs for Measuring Effects
  - Dealing with random error
  - Dealing with confounding
  - Dealing with bias
- Biostatistical analyses for analyzing data

Measures of Disease

- Incidence
  - Cumulative Incidence
  - Incidence Density

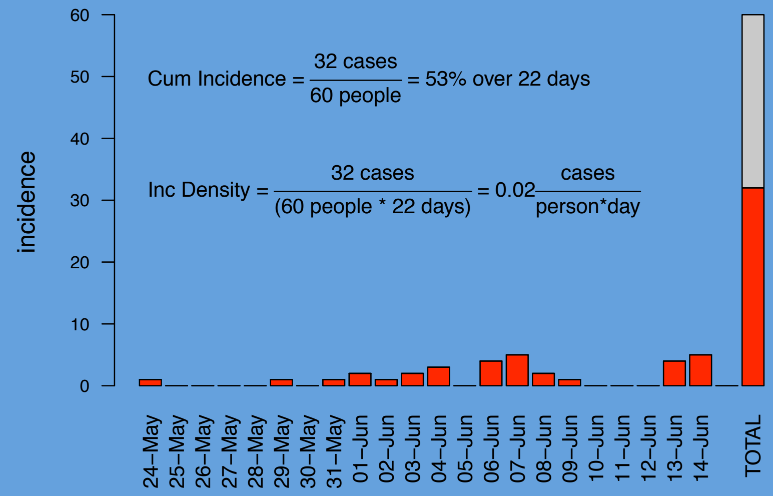

- Prevalence
  - Point Prevalence
  - Period Prevalence

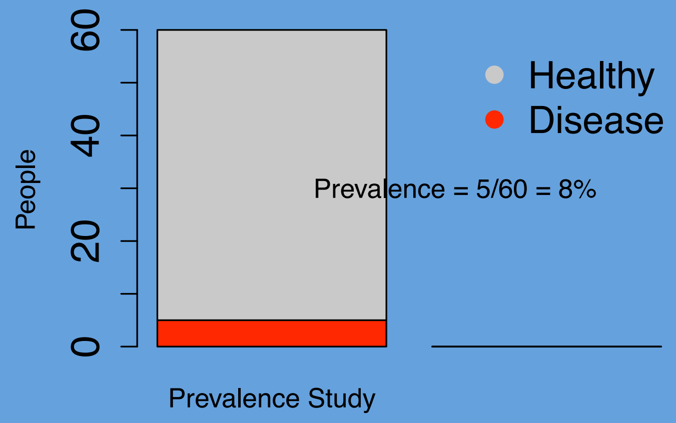

- Survivorship
  (time to event, such as mortality)

Measures of Covariates (risk factors)

- Binary: gender, smoker, circumcised
- Nominal/Categorical: geographic region
- Continuous: birth weight, T-cell count
- Ordinal: education, socioeconomic status (SES)

Measures of Effect

- How do you measure the effect of a risk factor on a disease?

Example

How could you measure whether circumcision reduces the risk of HIV infection?

Measures of Effect

- Compare measure of disease across levels/values of risk factors

- Relative Risk
  Ratio of rates or proportions
    - Prevalence Ratio
    - Cum. Incidence Ratio
    - Incidence Density Ratio
    - Odds Ratio

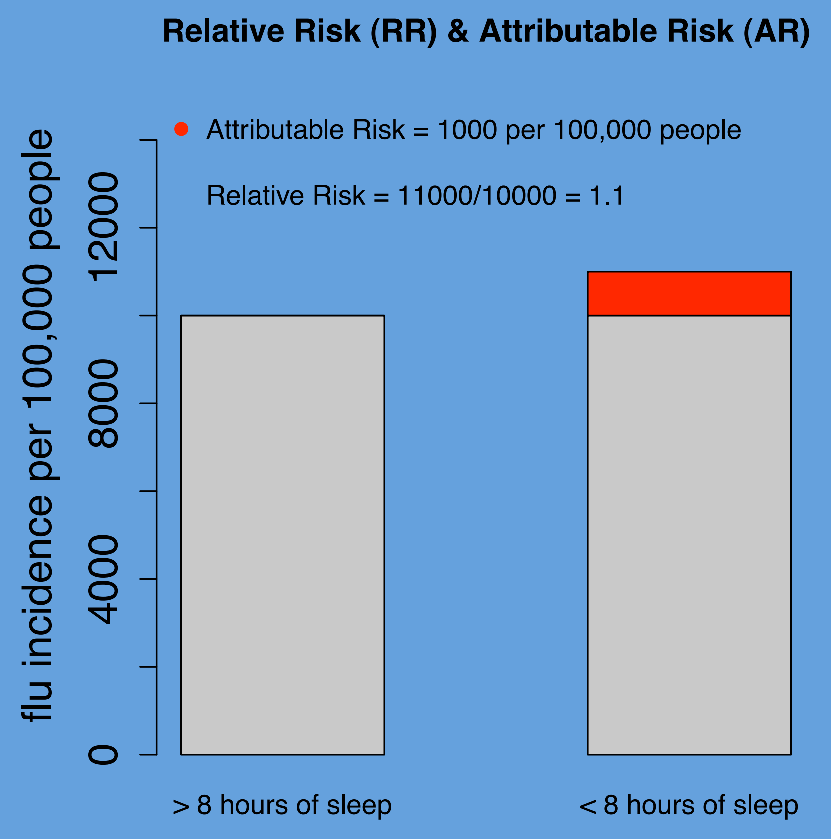

- Attributable Risk
  Subtract rates or proportions

Contingency Tables: Relative Risk (RR)

	Disease	No Disease	Total (Margins)
Exposed	a	b	a+b
Not exposed	c	d	c+d
Total (Margins)	a+c	b+d	a+b+c+d

Cumulative Incidence Ratio is the ratio of cumulative incidence in the exposed population divided by the cumulative incidence in the unexposed population.

$$CIR = \frac{\frac{a}{a+b}}{\frac{c}{c+d}}$$

CIR < 1 means exposure correlates with reduced risk of disease
CIR > 1 means exposure correlates with increased risk of disease

Epidemiologic Studies

- Descriptive Epidemiology
  - Baseline data on distribution of disease
  - Surveillance

- Analytic Epidemiology – Measure Effect
  - Prospective Cohort Studies
  - Cross-sectional Studies
  - Retrospective Case-Control Studies
  - Ecologic Studies
  - Randomized Controlled Trials

Observational (Prospective Cohort, Cross-sectional, Retrospective Case-Control, Ecologic Studies)

Experimental (Randomized Controlled Trials)

Cohort Studies

- Follow a selected population through time
  - Establishes temporal relationships
  - Can measure incidence
- Takes lots of resources, money, & time!
- Poor design for rare diseases.

Relative Risk: Incidence Density Ratios

	Disease	No Disease	Total (Margins)
Exposed	a	-	PY_e
Not exposed	c	-	PY_0
Total (Margins)	a+c	-	$PY_e + PY_0$

Incidence Density Ratio is the ratio of incidence density of the exposed population to that of the unexposed population.

$$IDR = \frac{\frac{a}{PY_e}}{\frac{c}{PY_0}}$$

IDR < 1 means exposure correlates with reduced risk of disease
IDR > 1 means exposure correlates with increased risk of disease

Cross-Sectional Studies

- Snapshot of diseases & risk factors.
- Cannot establish temporal relationship.
- Relatively cheap & easy.
- Population must be large to study rare disease
- Not great for diseases of short duration. Why?

Case-Control Studies

- Compare diseased individuals to chosen controls.
  - Quality of study depends entirely on how controls are chosen.
- Good for rare diseases.
- Relatively cheap & quick.

$$PR = \frac{\frac{a}{a+b}}{\frac{c}{c+d}}$$

Case Control Studies: Odds Ratios

Controls: Number chosen by researcher.

	Disease	No Disease	Total (Margins)
Exposed	a	b	a+b
Not exposed	c	d	c+d
Total (Margins)	a+c	b+d	a+b+c+d

Odds ratio is the ratio of odds in the diseased population divided by the odds in the non-diseased population.

$$OR = \frac{a/c}{b/d} = \frac{ad}{bc}$$

OR < 1 means exposure correlates with reduced risk of disease
OR > 1 means exposure correlates with increased risk of disease

Randomized Controlled Trials

- Experimental or Intervention Studies
- Establishes temporal relationships
- Addresses confounding (more to come)

Ecologic Studies

- Measurements made at population rather than individual level.

- Weaker inference, but easier to gather data.

Measures of Covariates (risk factors)

- Binary: gender, smoker, circumcised
- Nominal/Categorical: geographic region
- Continuous: birth weight, T-cell count
- Ordinal: education, socioeconomic status (SES)

What do *Introductory Epidemiology* courses teach?

- Measures of Disease
- Measures of Effect (of a risk factor)
- Study Designs for Measuring Effects
  - Dealing with random error
  - Dealing with confounding
  - Dealing with bias
- Biostatistical analyses for analyzing data

Random Error

- How many people must be in a study for the measure of effect to believable?

- Statistical Approach:
  Assign probabilities to our findings being a product of random error rather than a real phenomenon.

Bias

Difference between observed value and true value due to all causes other than random error.

Bias does not go away with greater sample size!

Bias must be dealt with during study design!

Selection Bias

Error due to systematic differences between those who take part in the study and those who do not.

John Last, Dictionary of Epidemiology

Information Bias

A flaw in measuring exposure or outcome data that results in different quality (accuracy) of information between comparison groups.

John Last, Dictionary of Epidemiology

Confounding

Literacy → HIV Status

	HIV+	HIV-
Literate	660	340
Illiterate	180	820

$$PR = \frac{660/1000}{180/1000} = 3.67$$

What if some of the study population were much younger than others?

Confounding

Pooled	HIV+	HIV-
Literate	660	340
Illiterate	180	820

$$PR_{all} = \frac{660/1000}{180/1000} = 3.67$$

6-15 years old	HIV+	HIV-
Literate	30	270
Illiterate	90	810

$$PR_{6-15\,yrs} = \frac{30/300}{90/900} = 1$$

16-24 years old	HIV+	HIV-
Literate	630	70
Illiterate	90	10

$$PR_{16-24\,yrs} = \frac{630/700}{90/100} = 1$$

6-15 year olds: Literacy = 300/1200 = 25%

16-24 year olds: Literacy = 700/800 = 87.5%

Confounding

	HIV+	HIV-
Literate	660	340
Illiterate	180	820

$$PR = \frac{660/1000}{180/1000} = 3\text{[illegible]}7$$

$$PR = \frac{30/300}{90/900} = 1$$

$$PR = \frac{630/700}{90/100} = 1$$

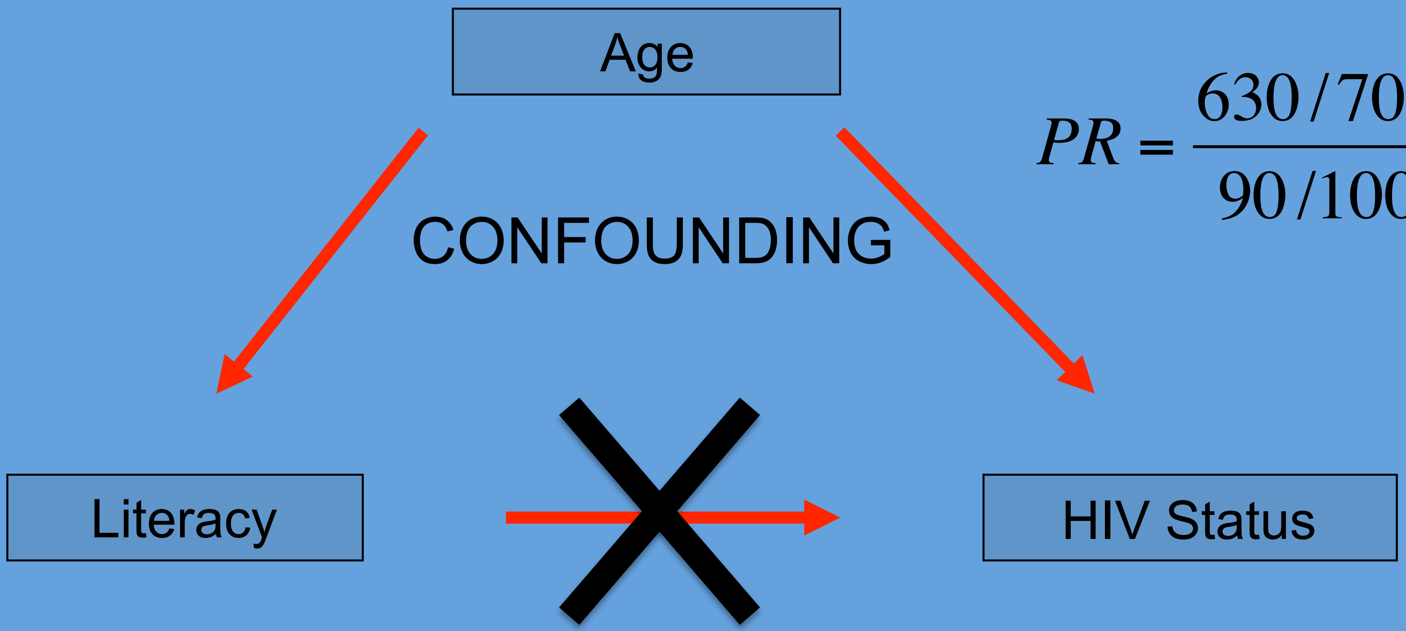

Biostatistical Analyses

- Permutation Tests
- Chi Squared Test
- Generalized Linear (Mixed) Models
  - Normal Regression
  - Logistic Regression
  - Poisson Regression
  - Negative Binomial Regression
- Survival Analysis

Statistical Models	Dynamic Models
Account for bias and random error to find correlations that may imply causality.	Systems Approach: Explicitly model multiple mechanisms to understand their interactions.
Often the first step to assessing relationships.	Links observed relationships at different scales.
Assume independence of individuals (at some scale, *i.e.* clusters).	Explicitly focuses on dependence of individuals

By developing dynamic models in a probabilistic framework we can account for dependence, random error, and bias while linking patterns at multiple scales.

Questions in Epidemiology

Statistical Models

- Is HIV status positively associated with the risk of TB infection?

Dynamic Models

- Based on increased TB risk due to HIV, how much should we expect TB notification rate to increase for a given HIV prevalence?

Questions in Epidemiology

Statistical Models

- Are Insecticide Treated Bednets (ITNs) or Indoor Residual Spraying (IRS) more effective for controlling malaria?

Dynamic Models

- How do we expect the age-distribution of malaria incidence to change after implementing ITNs or IRS?
