# Supplementary material for: How to Make Epidemiological Training Infectious
Source: PLoS Biol. 2012 Apr 3;10(4):e1001295. doi: 10.1371/journal.pbio.1001295 (PMC3317897; doi:10.1371/journal.pbio.1001295)
Supplement: Figure S5 — Lecture slide 5: introduction to statistical philosophy. (PDF) [file pbio.1001295.s012.pdf]

Introduction to statistical philosophy

Statistics

- We use statistics to confirm effects, estimate parameters, and predict outcomes
- The last 2 times I came to Cape Town, it rained, but only on Sunday
  - *Confirmation*: In Cape Town, it rains more on Sundays than other days
  - *Estimation*: I think it has rained on $> 30\%$ of Sundays in Cape Town
  - *Prediction*: I don't think it will rain on Tuesday
- The last *20* times I came to Cape Town, it rained, but only on Sunday

Confirmation

- We want to know why prevalence increased in this village from 25% to 67%.
- First ask: what are the data?
  - 1/4 to 4/6
  - 5/20 to 12/18
  - 75/300 to 180/270

Confirmation example

- We measure the average heights of children raised with and without vitamin A supplements
- What sort of test could we do to confirm whether we believe this difference is due to chance?

Frequentist approach

- Make a null model
- Test whether the effect you see could be due to chance
  - What is the probability of seeing exactly a 1.52 cm difference in average heights?
- Test whether the effect you see *or a larger effect* could be due to chance

Frequentist conclusions

- If your effect size is unlikely to be caused by chance, you can believe the effect
- If your effect could easily be caused by chance, don't believe the effect
  - *But don't conclude that there is no effect*

Why don't we accept the null hypothesis?

- Why *do* we reject the null hypothesis?

Low P values

- What causes low P values?
  - Large differences
  - Lots of data
  - Less noise
  - A well-specified model
  - Ability to disentangle covariates

High P values

- What causes high P values?
  - Small differences
  - Less data
  - More noise
  - An inappropriate model
  - Ability to disentangle covariates

A high P value is not evidence for anything!

- Ever
- What kind of evidence should we use instead?
  - Confidence intervals
  - "Non-inferiority", "non-superiority", or both
    * A low P value rejecting the idea that the difference is large

Confidence intervals

- How do we estimate the *size* of an effect
- Frequentists estimate confidence intervals by asking which values could be falsified (if they were considered as null hypotheses)
- Example:
  - 12/30 women observed are HIV positive. What is my estimate for the prevalence?
  - What if it's 120/300?
    * What else do we need to know?

Frequentist philosophy

- I am absolutely convinced that any two populations I can describe are different.
- I will never accept the null hypothesis! At least in biology
  - Why then does it make sense for me to test the null hypothesis?
- One village has 12/25 positive tests, another has 10/27. What should I conclude?

Example: flipping a coin

- I flip a coin 8 times, and get heads 8 times. Is the coin fair?
  - A frequentist would do the same calculation if they were just handed the coin by a magician, or if they stole it while touring the mint
  - A Bayesian needs a starting point to model from
- My *a priori* assumption is that there is a 1 in 800 probability that the coin has two heads (otherwise, it's fair)
  - What do I think after flipping 8 heads?

The Bayesian approach

- A Bayesian approach to statistics requires modeling what you think is happening, not just a null model
- Much "bolder" than a frequentist approach
  - We assume more, and we can conclude more, including predictions of the future

Bayesian inference

- We want to go from a *statistical model* of how our data are generated, to a probability model of parameter values
  - Requires *prior* distributions describing the assumed likelihood of parameters before these observations are made
  - Use Bayes theorem to calculate posterior distribution – likelihood after taking data into account

Bayesian Advantages

- Assumptions more explicit
- Probability statements more straightforward
- Very flexible
- Can combine information from different sources
- Can make rigorous predictions about the future

Bayesian Disadvantages

- More assumptions required
  - Lacks elegance of permutation approaches
- More difficult to calculate answers

Prior distributions

- You should usually start with a prior distribution that has little "information"
  - Let the data do the work
- The "posterior" from one analysis can be the prior for the next analysis

P values

- Bayesian P values have a more direct interpretation than frequentist P values:
  - We calculate the posterior probability that our effect size is positive
  - If we are willing to rely on our assumptions, this gives the actual probability that our hypothesis is true
- We can also reject our hypothesis directly if the probability that it's true is smaller than a pre-specified value (although people usually don't do this)

Credible intervals

- Credible intervals are the Bayesian analogue of confidence intervals
- Since a Bayesian model is a complete probability model, the credible interval is simply an interval that we believe contains the correct answer with probability 95% (half of that probability is on each side of our median estimate).

A concrete example

- I observe 3 shooting stars in one hour of observing the sky.
- What is my credible interval for the rate of shooting stars?

Shooting stars

- For each rate, our likelihood of observing N events in time T if the true rate is r is a Poission distribution with mean rT:
  - $\frac{(rT)^N \exp(-rT)}{N!}$
- We choose an improper, uniform prior over $\log r$, equivalent to $\pi(r) = 1/r$.
- The posterior distribution is then proportional to:
  - $(rT)^{N-1} \exp(-rT)$, which gives a gamma distribution with mean N/T (the observed rate), and CV $1/\sqrt{N}$.

MCMC sampling

- Bayesian methods are very flexible: We can write down reasonable priors, and likelihoods, to cover a wide variety of assumptions and situations
- Unfortunately, we usually can't solve exactly
- Instead we use Markov chain Monte Carlo methods to sample randomly from the posterior distribution
  - Simple in theory, but may be difficult in practice
  - You may not even know whether you have calculated for long enough

©2010 by Jonathan Dushoff. May be reproduced and distributed, with this notice, for non-commercial purposes only.
