# Supplementary material for: How to Make Epidemiological Training Infectious
Source: PLoS Biol. 2012 Apr 3;10(4):e1001295. doi: 10.1371/journal.pbio.1001295 (PMC3317897; doi:10.1371/journal.pbio.1001295)
Supplement: Figure S6 — Lecture slide 6: introduction to likelihood. (PDF) [file pbio.1001295.s013.pdf]

Introduction to Likelihood

Meaningful Modeling of Epidemiologic Data, 2011
AIMS, Muizenberg, South Africa

Steve Bellan
MPH Epidemiology
PhD Candidate
Department of Environmental Science, Policy & Management
University of California at Berkeley

In a population of 1,000,000 people with a true prevalence of 30%, the probability distribution of number of positive individuals if 100 are sampled:

$$f(x) = \binom{100}{x}(0.3)^x(0.7)^{100-x}$$

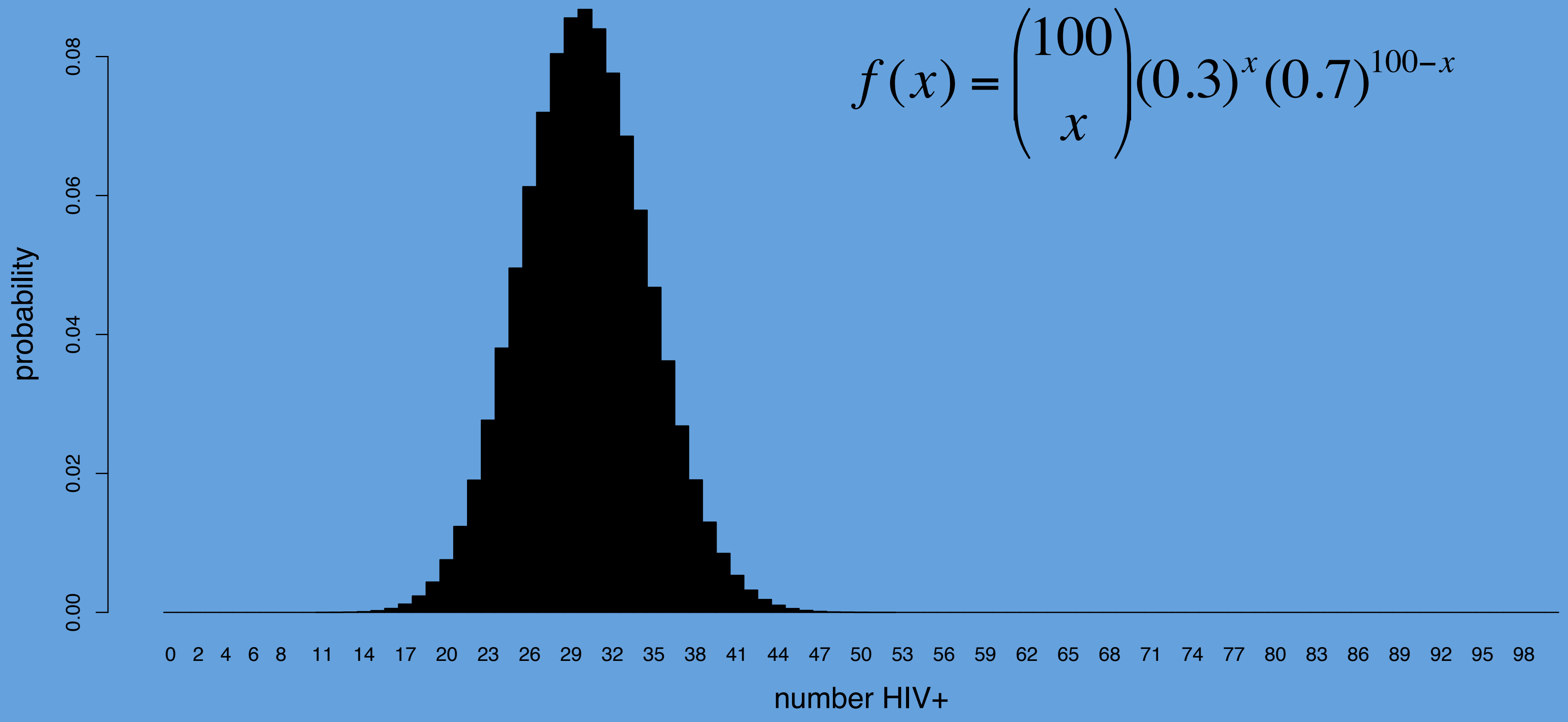

barplot(dbinom(x = 0:100, size = 100, prob = .3), names.arg = 0:size)

In a population of 1,000,000 people with a true prevalence of 30%, the probability distribution of number of positive individuals if 100 are sampled:

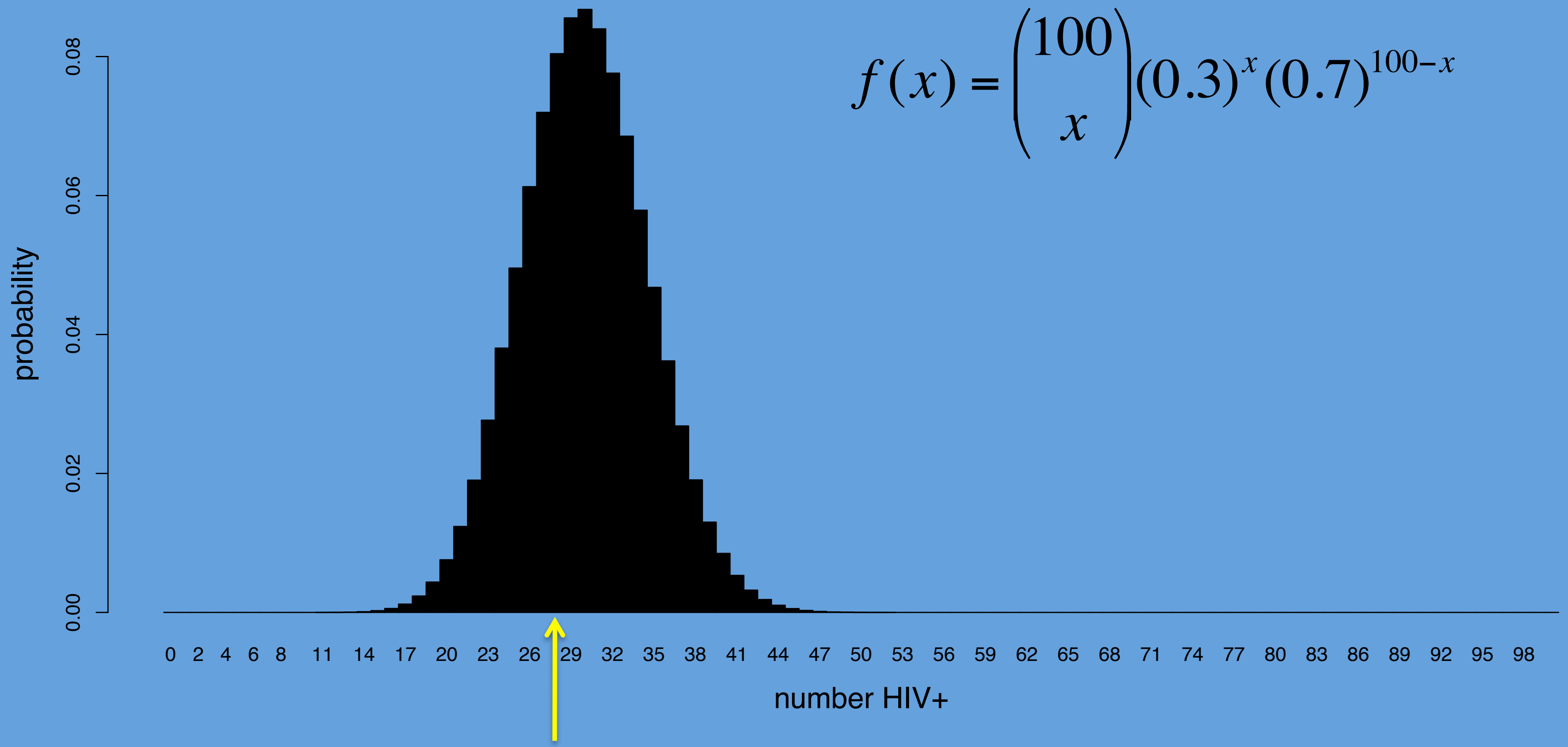

$$f(x) = \binom{100}{x}(0.3)^x(0.7)^{100-x}$$

We sample 100 people once and 28 are positive:

```
> rbinom(n = 1, size = 100, prob = .3)
[1] 28
```

We don’t know the true prevalence!

But we can calculate the probability of 28 or a more extreme value occurring for a given prevalence.

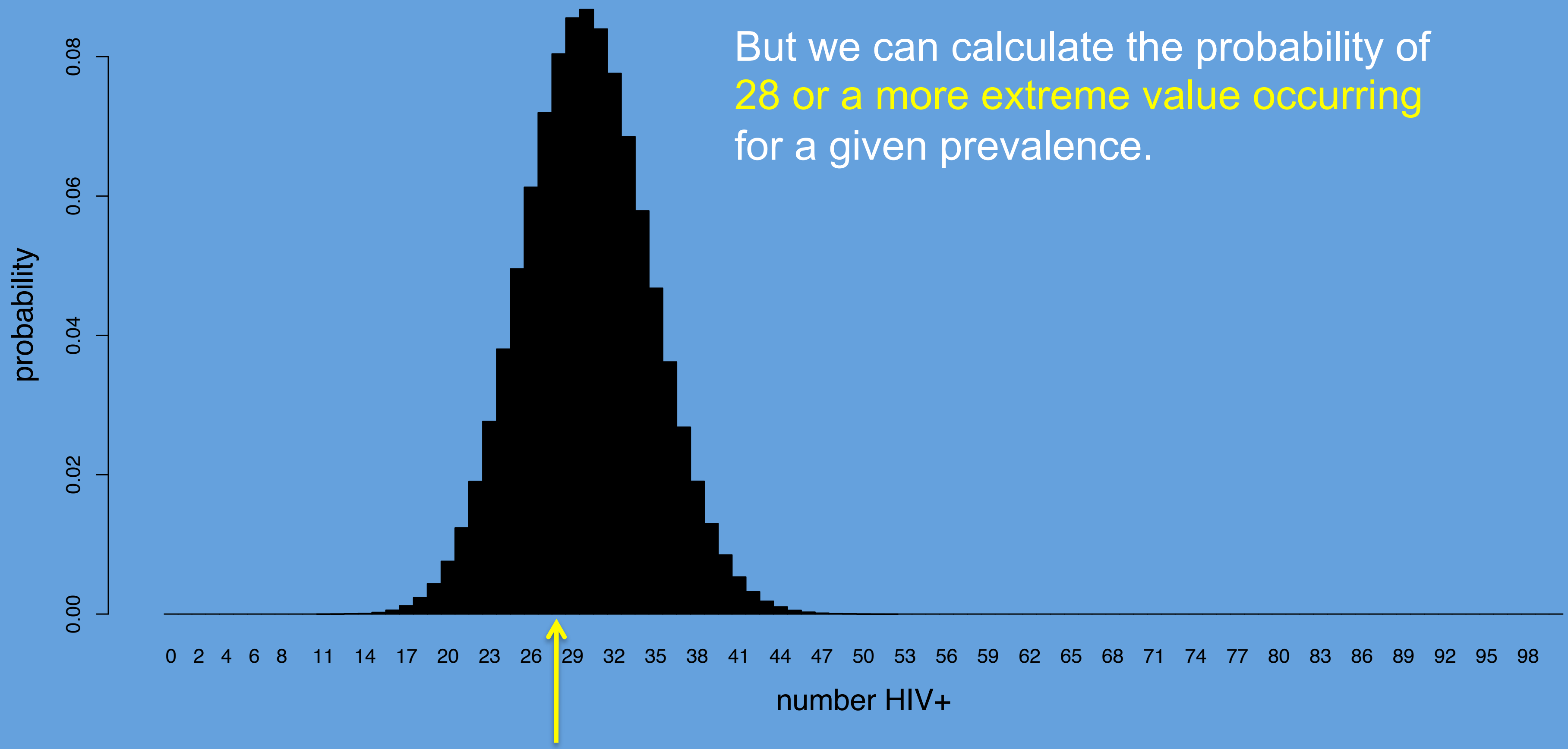

We sample 100 people once and 28 are positive:

```
> rbinom(n = 1, size = 100, prob = .3)
[1] 28
```

Cumulative Probability & P Values

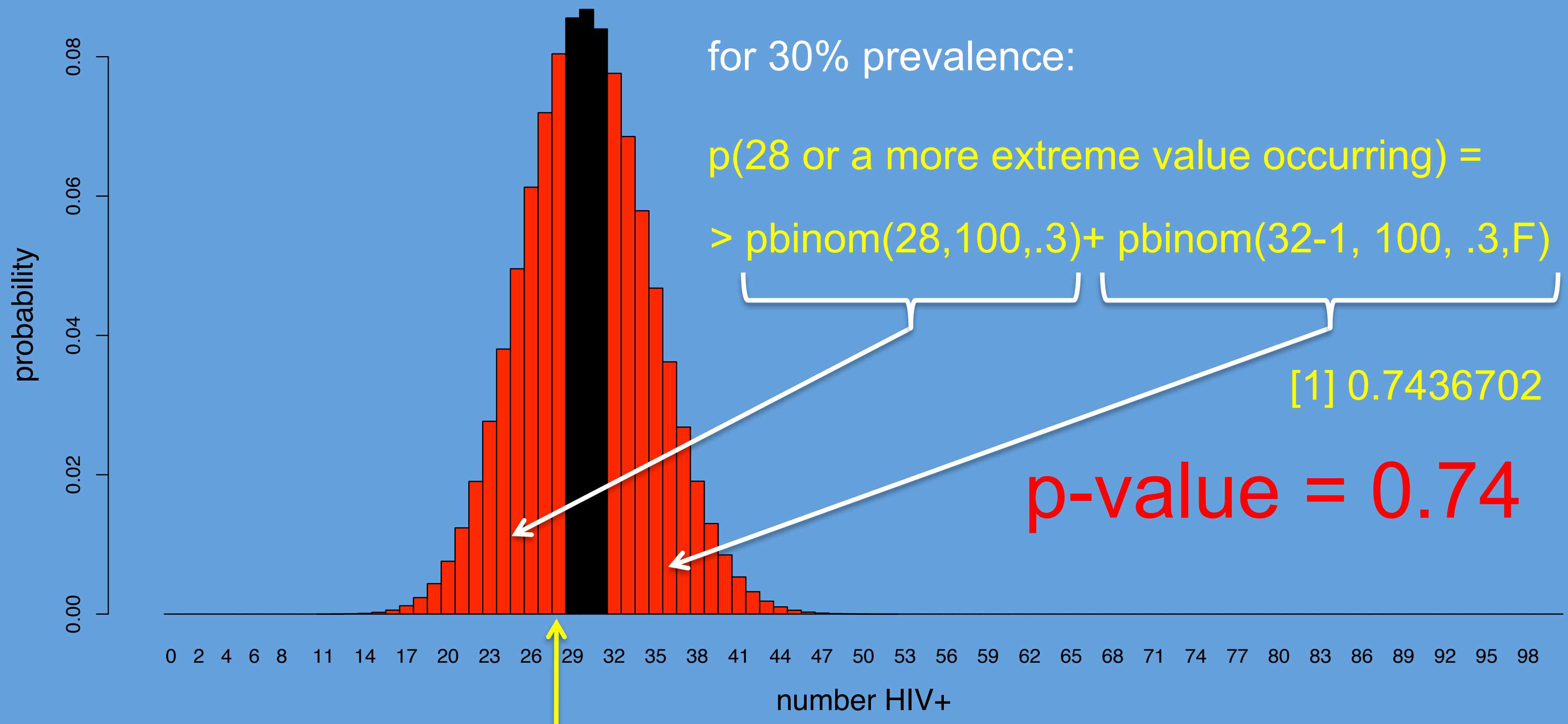

for 30% prevalence:

p(28 or a more extreme value occurring) =

> pbinom(28,100,.3)+ pbinom(32-1, 100, .3,F)

[1] 0.7436702

p-value = 0.74

We sample 100 people once and 28 are positive.

If true prevalence were 15%, then p(28 or more extreme) is

pbinom(2, 100, 0.15, lower.tail = T) + pbinom(28−1, 100, 0.15, lower.tail = F)

p = 0.000628

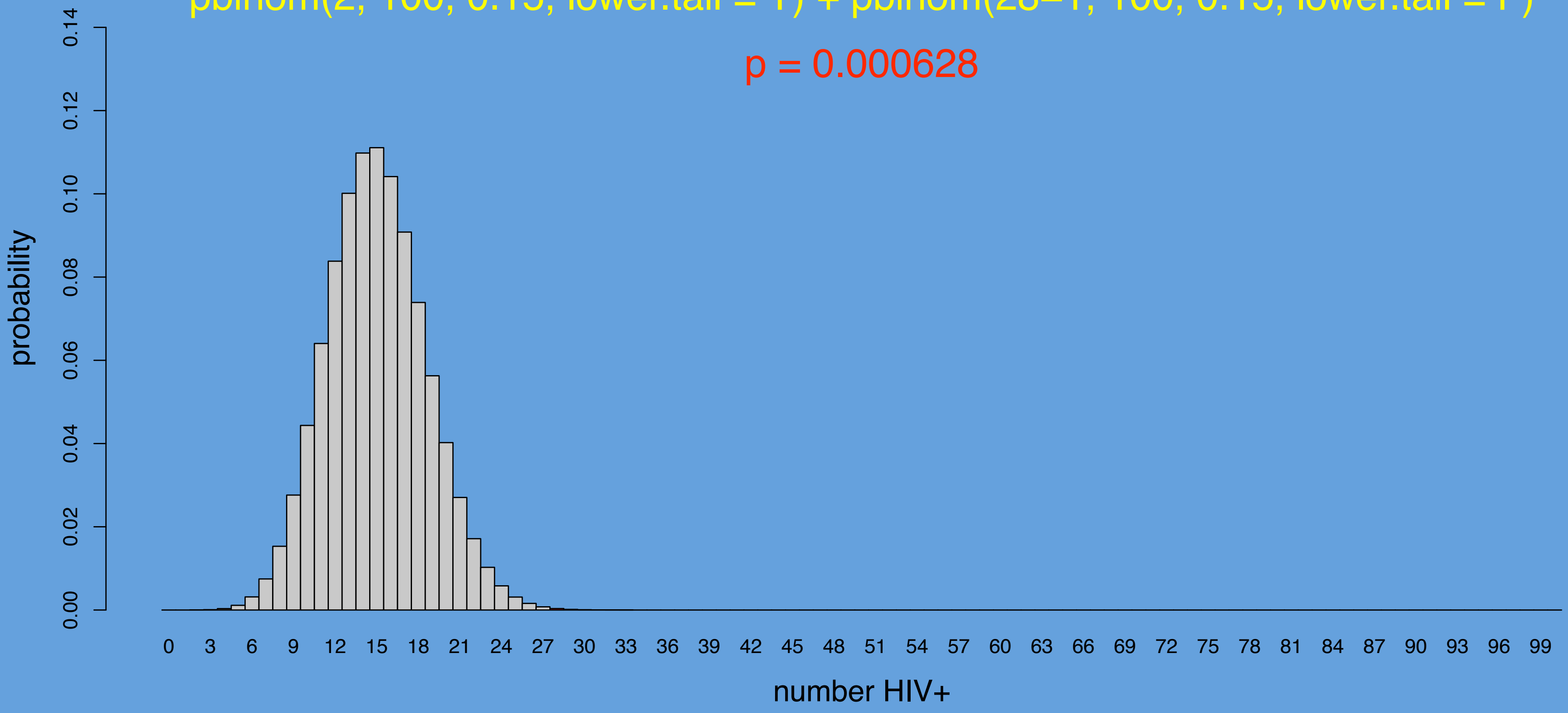

If true prevalence were 20%, then p(28 or more extreme) is

pbinom(12, 100, 0.2, lower.tail = T) + pbinom(28–1, 100, 0.2, lower.tail = F)

p = 0.0595

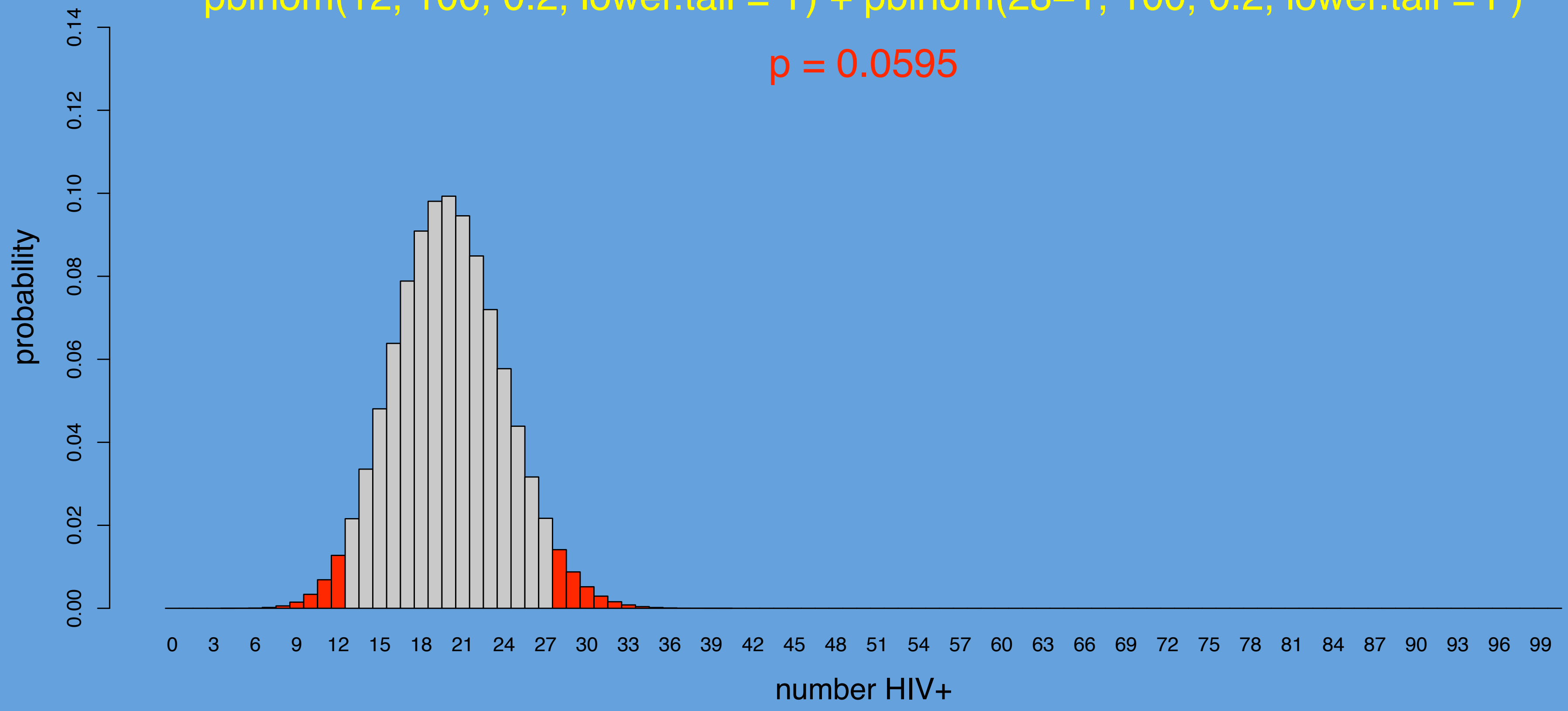

If true prevalence were 25%, then p(28 or more extreme) is

pbinom(22, 100, 0.25, lower.tail = T) + pbinom(28–1, 100, 0.25, lower.tail = F)

p = 0.564

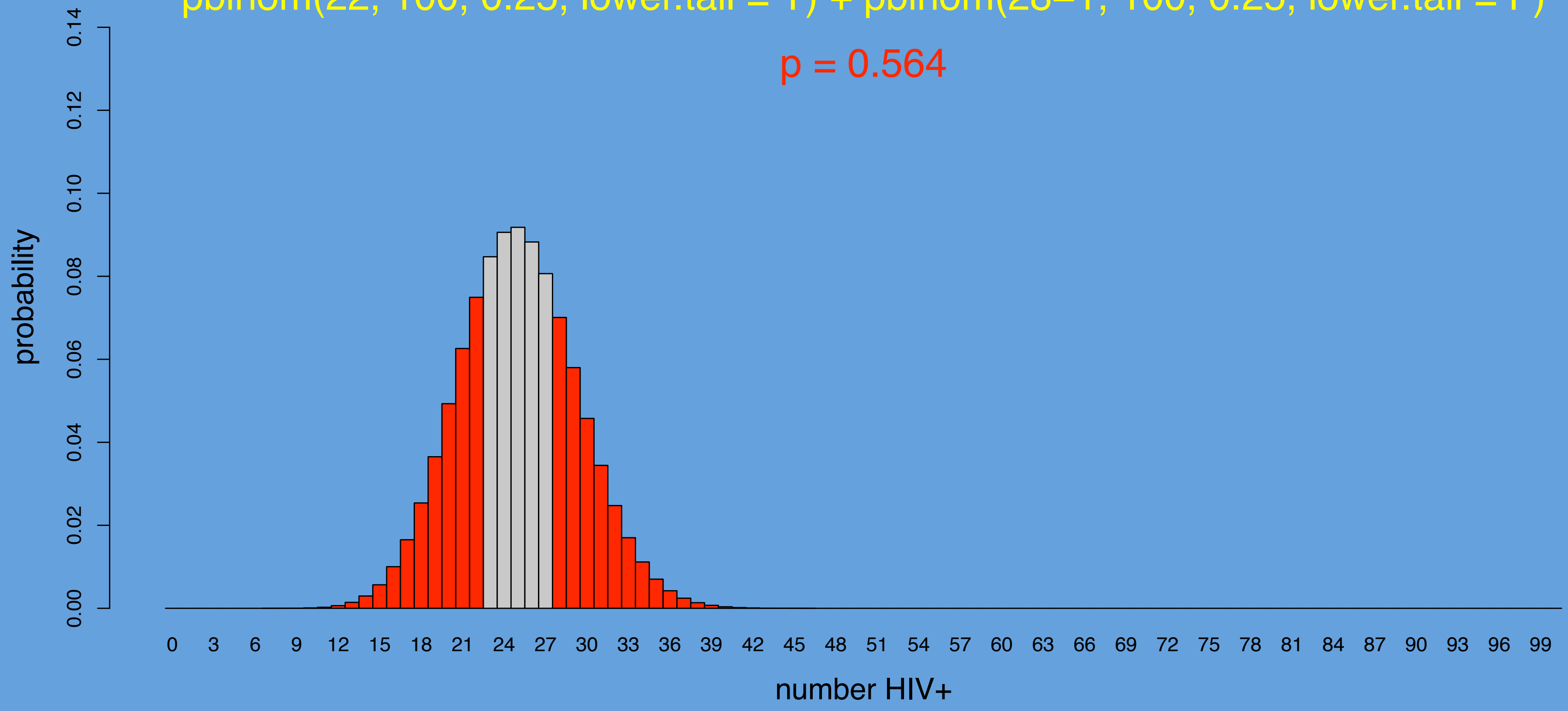

If true prevalence were 30%, then p(28 or more extreme) is

pbinom(28, 100, 0.3, lower.tail = T) + pbinom(32–1, 100, 0.3, lower.tail = F)

p = 0.744

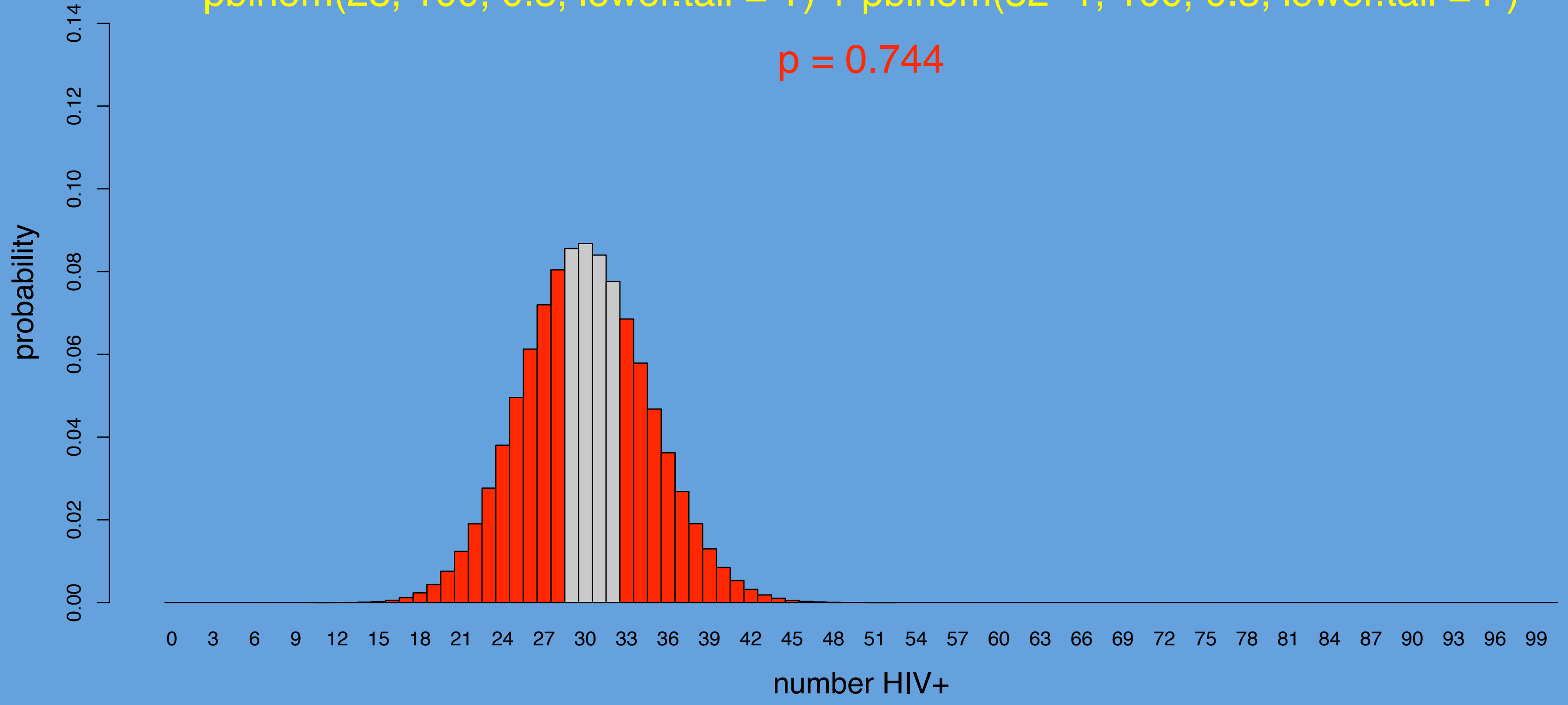

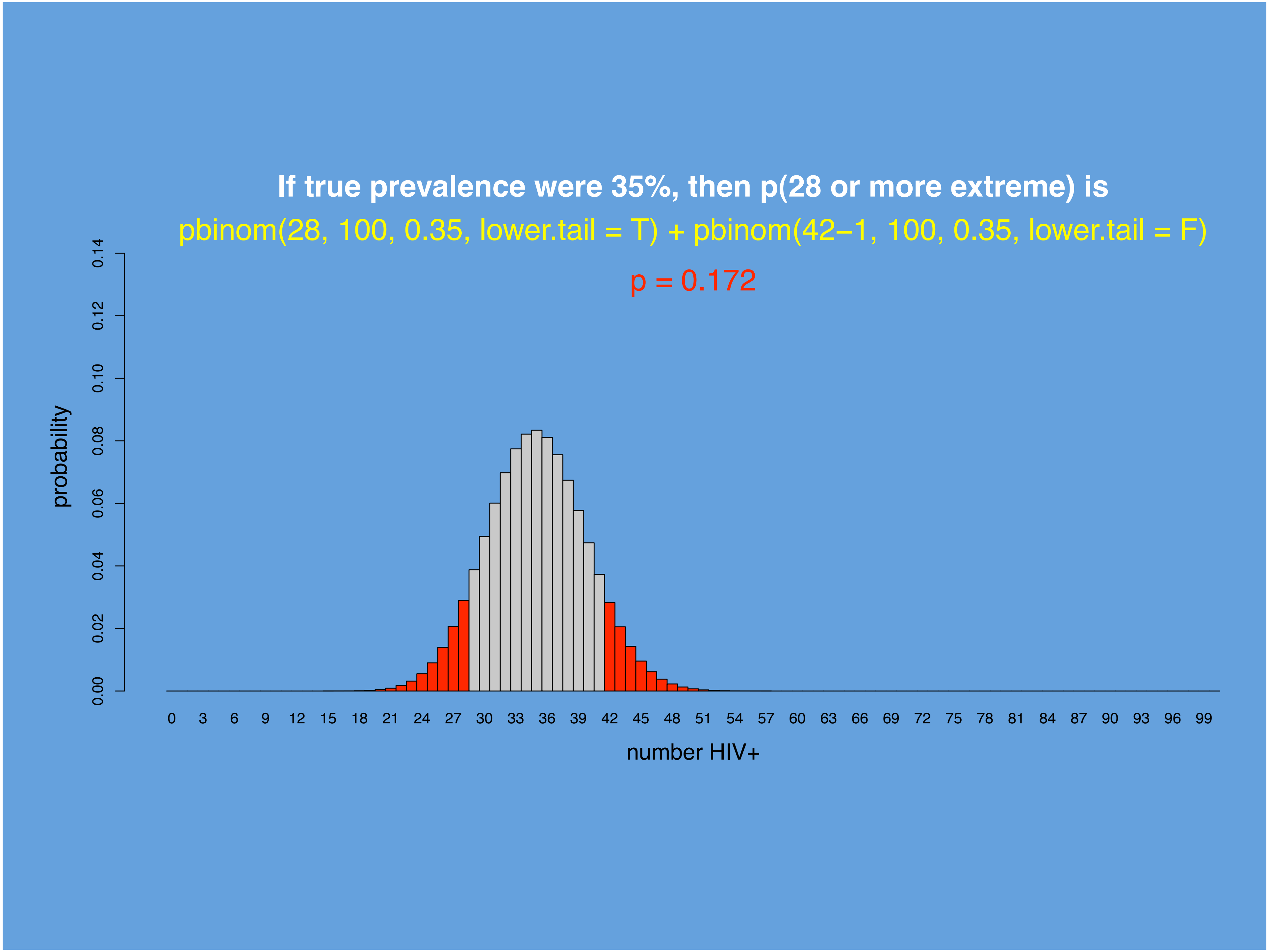
If true prevalence were 35%, then p(28 or more extreme) is
pbinom(28, 100, 0.35, lower.tail = T) + pbinom(42–1, 100, 0.35, lower.tail = F)
p = 0.172
probability
0.00
0.02
0.04
0.06
0.08
0.10
0.12
0.14
0 3 6 9 12 15 18 21 24 27 30 33 36 39 42 45 48 51 54 57 60 63 66 69 72 75 78 81 84 87 90 93 96 99
number HIV+

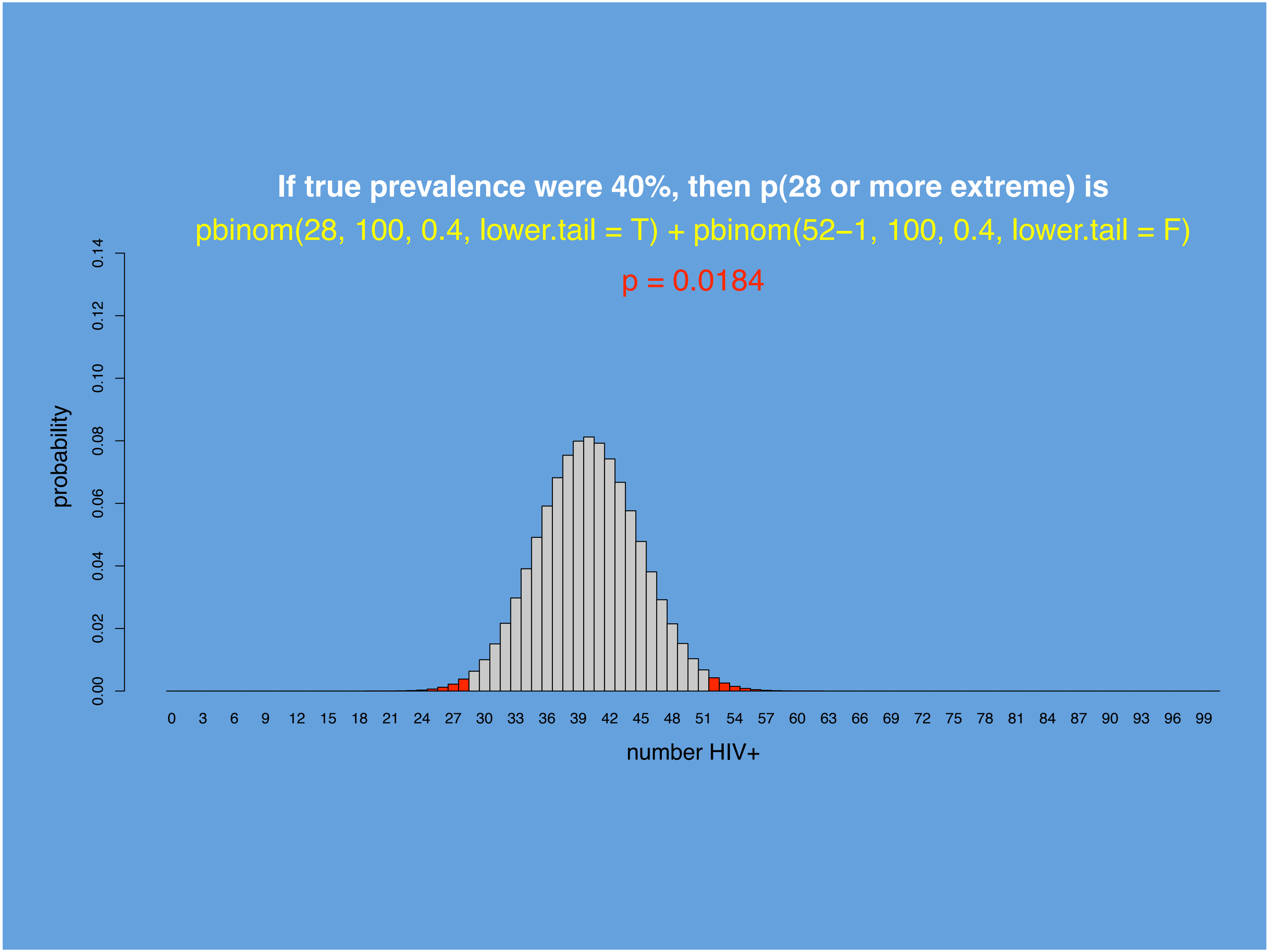

If true prevalence were 40%, then p(28 or more extreme) is
pbinom(28, 100, 0.4, lower.tail = T) + pbinom(52−1, 100, 0.4, lower.tail = F)
p = 0.0184
probability
0.00
0.02
0.04
0.06
0.08
0.10
0.12
0.14
0 3 6 9 12 15 18 21 24 27 30 33 36 39 42 45 48 51 54 57 60 63 66 69 72 75 78 81 84 87 90 93 96 99
number HIV+

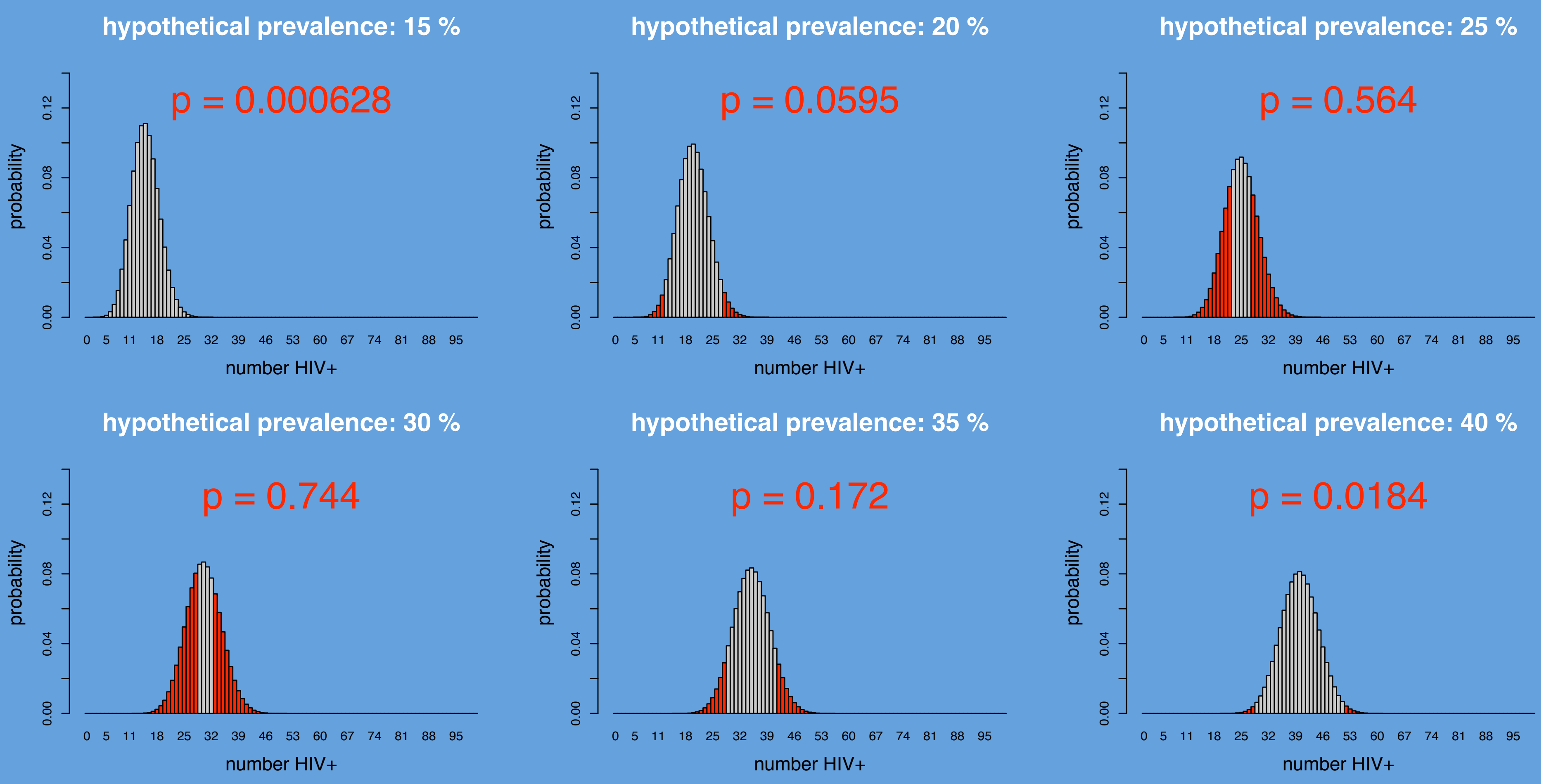
hypothetical prevalence: 15 %
p = 0.000628
hypothetical prevalence: 20 %
p = 0.0595
hypothetical prevalence: 25 %
p = 0.564
hypothetical prevalence: 30 %
p = 0.744
hypothetical prevalence: 35 %
p = 0.172
hypothetical prevalence: 40 %
p = 0.0184
probability
0.00
0.04
0.08
0.12
number HIV+
0 5 11 18 25 32 39 46 53 60 67 74 81 88 95

Which hypotheses do we reject?

IF GIVEN THE HYPOTHESIS

p value < cutoff

THEN REJECT HYPOTHESIS

Cutoff usually chosen as $\alpha = 0.05$

Which hypotheses do we reject?

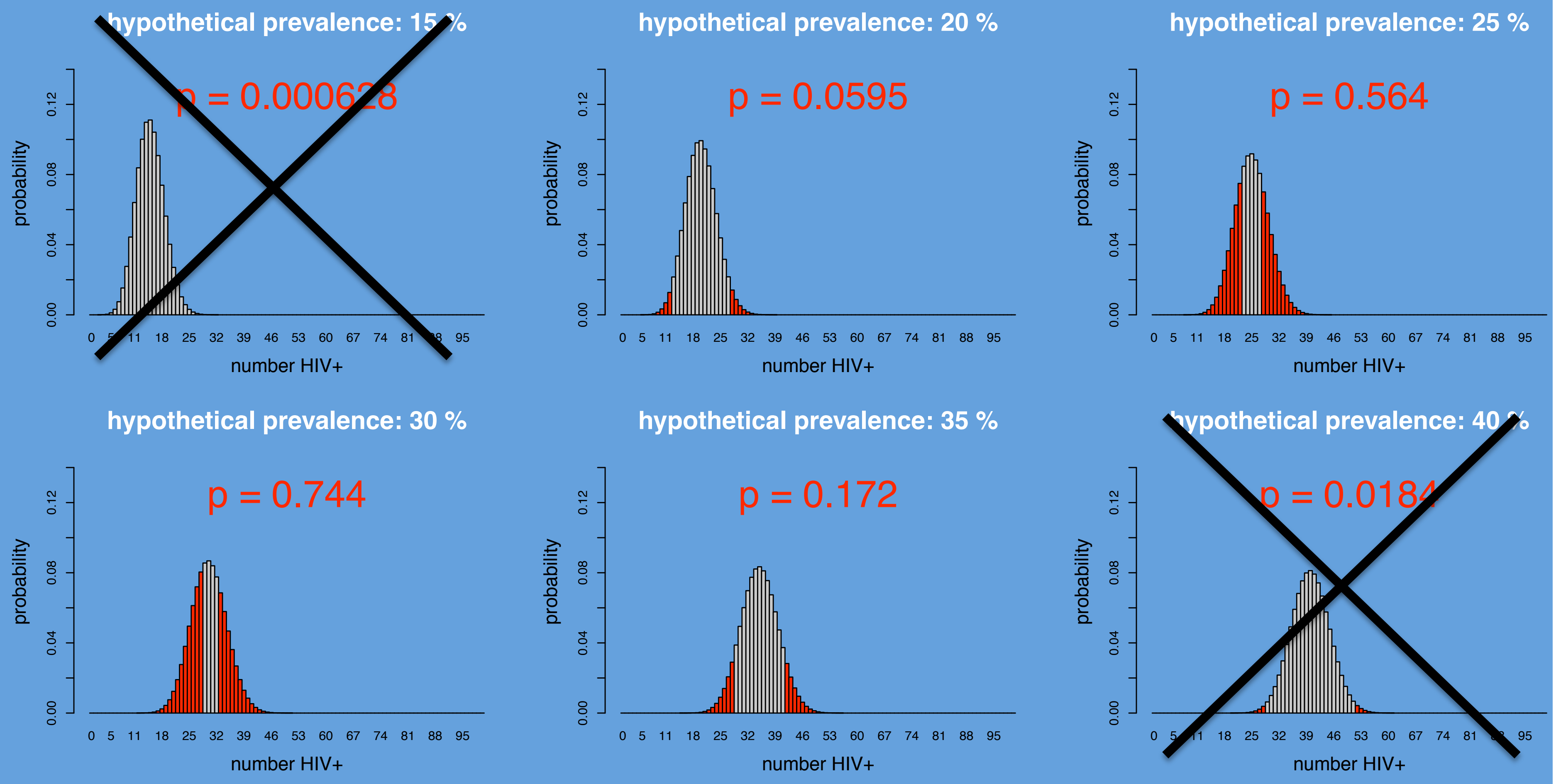

Which hypotheses do we NOT reject:
CONFIDENCE INTERVAL

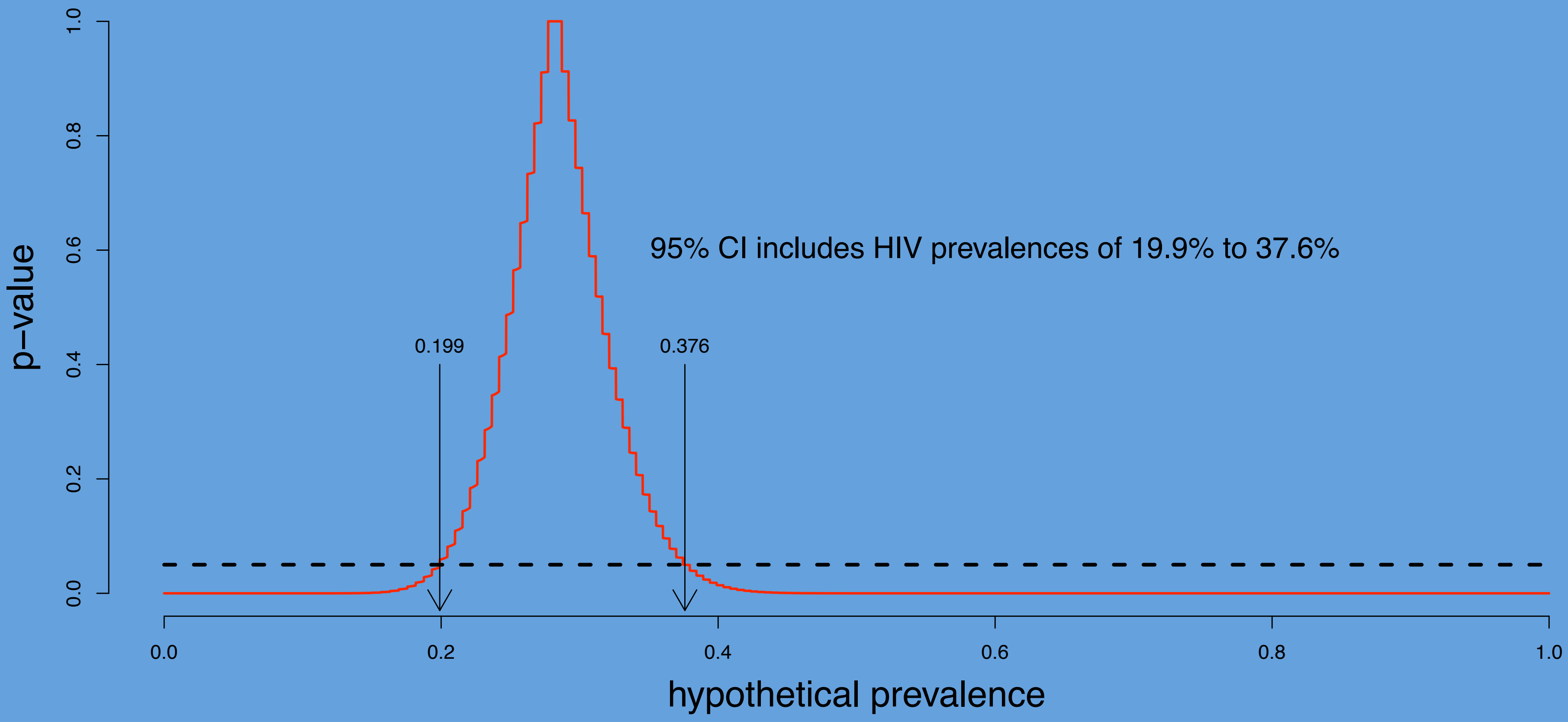

Let's take another approach

We don't know the true prevalence, but the probability that we had exactly 28/100 with 30% prevalence is:

> dbinom(x = 28, size = 100, prob = .3)
[1] 0.08041202

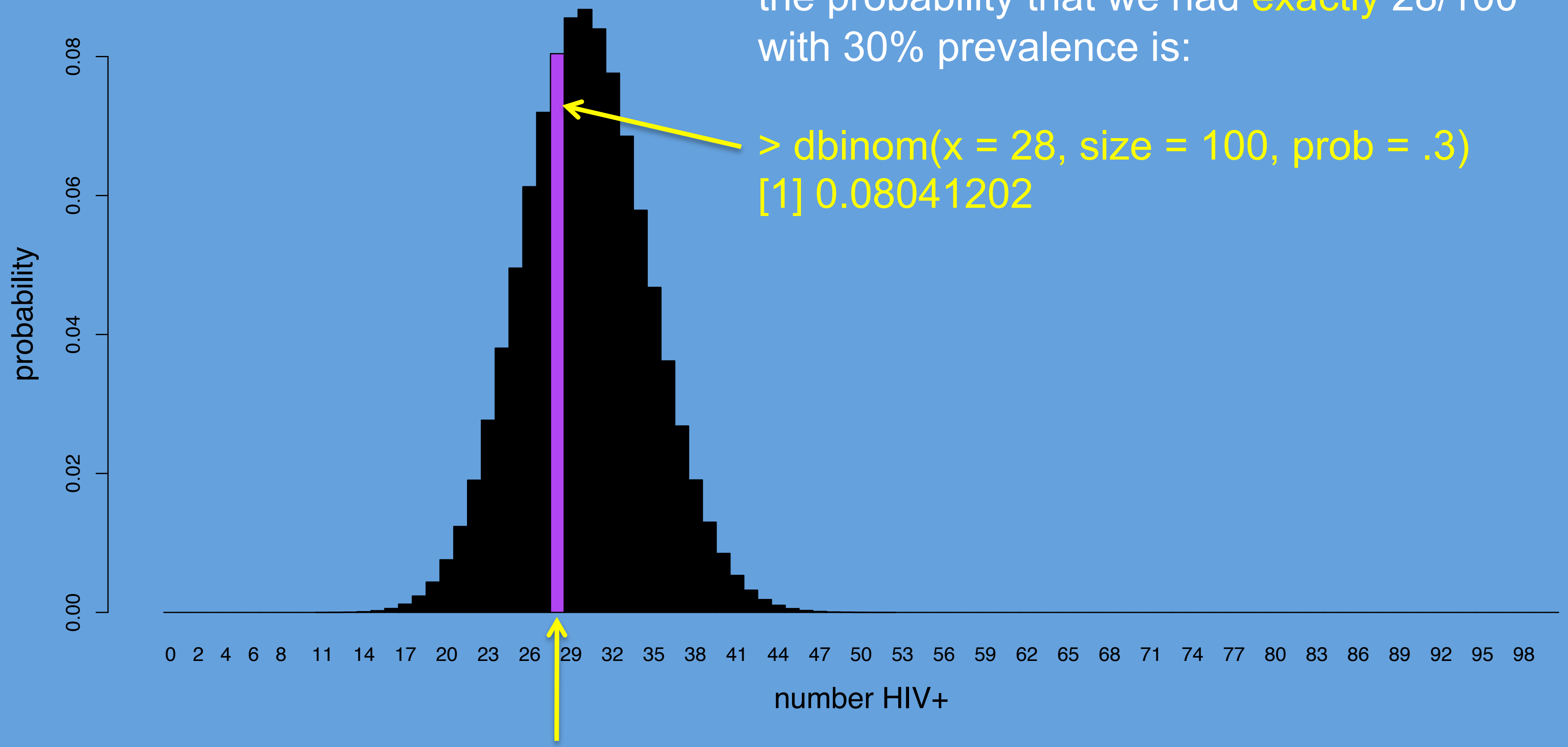

We sample 100 people once and 28 are positive:

> rbinom(n = 1, size = 100, prob = .3)
[1] 28

hypothetical prevalence: 15 %

dbinom(28, 100, 0.15) = 0.00035

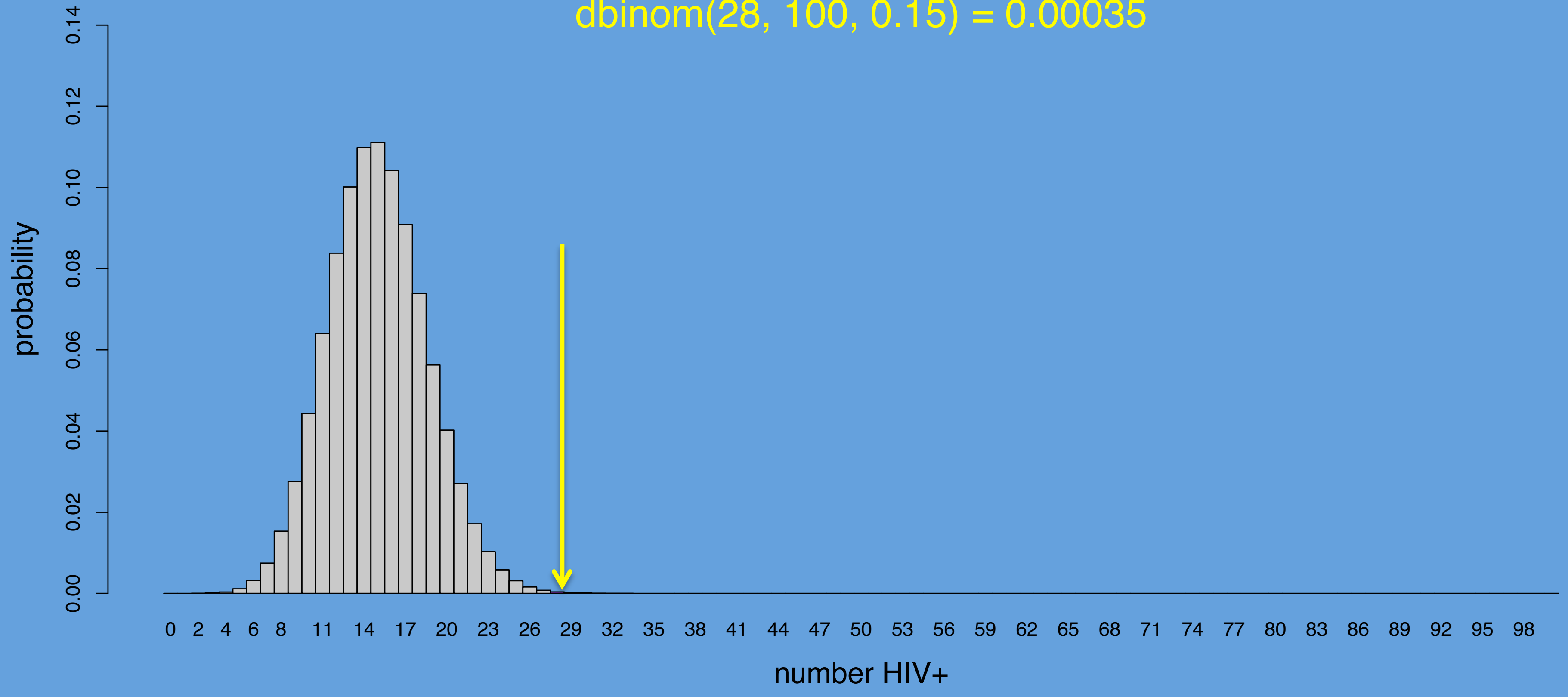

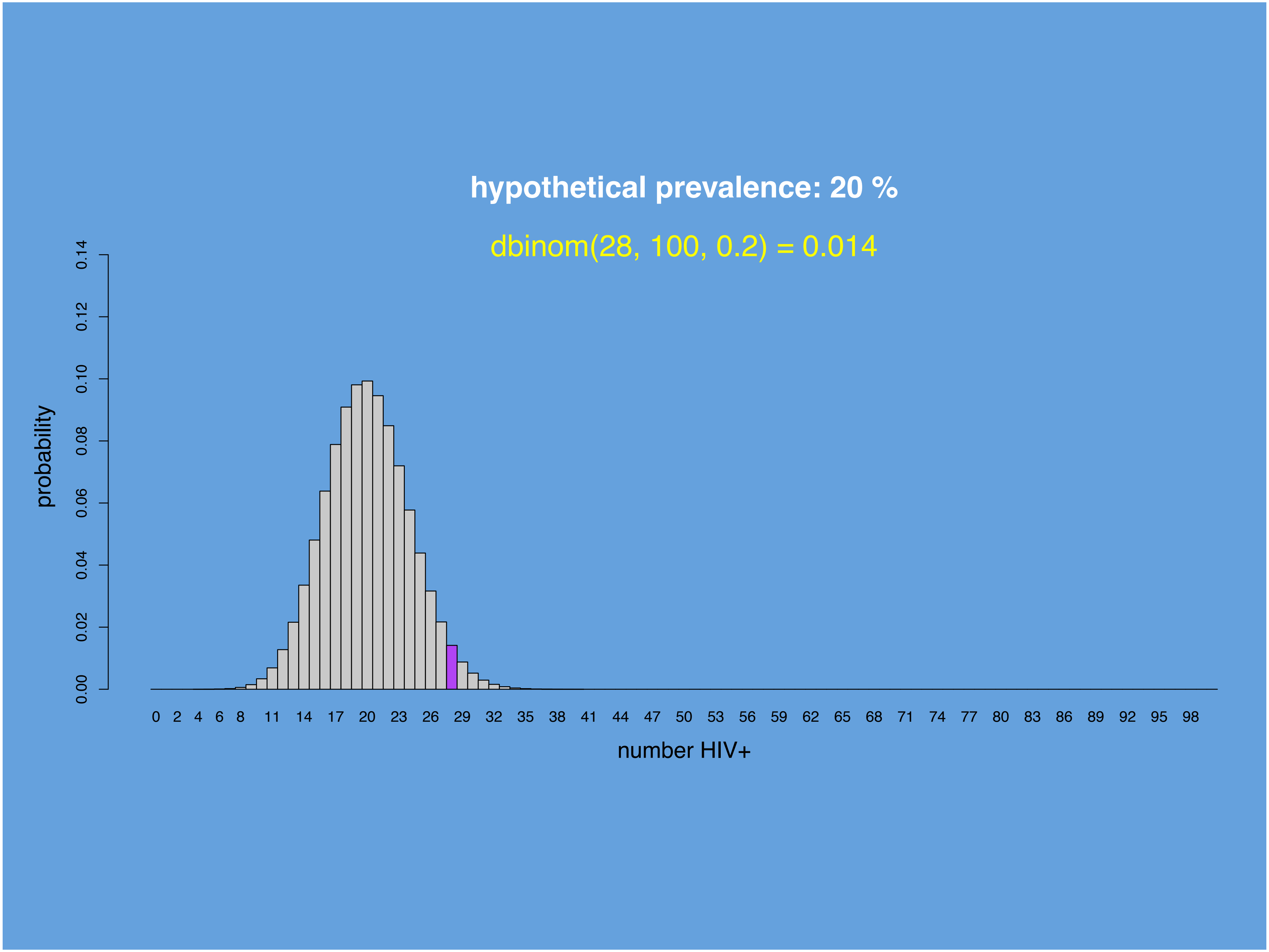

hypothetical prevalence: 20 %
dbinom(28, 100, 0.2) = 0.014
probability
0.00
0.02
0.04
0.06
0.08
0.10
0.12
0.14
0 2 4 6 8 11 14 17 20 23 26 29 32 35 38 41 44 47 50 53 56 59 62 65 68 71 74 77 80 83 86 89 92 95 98
number HIV+

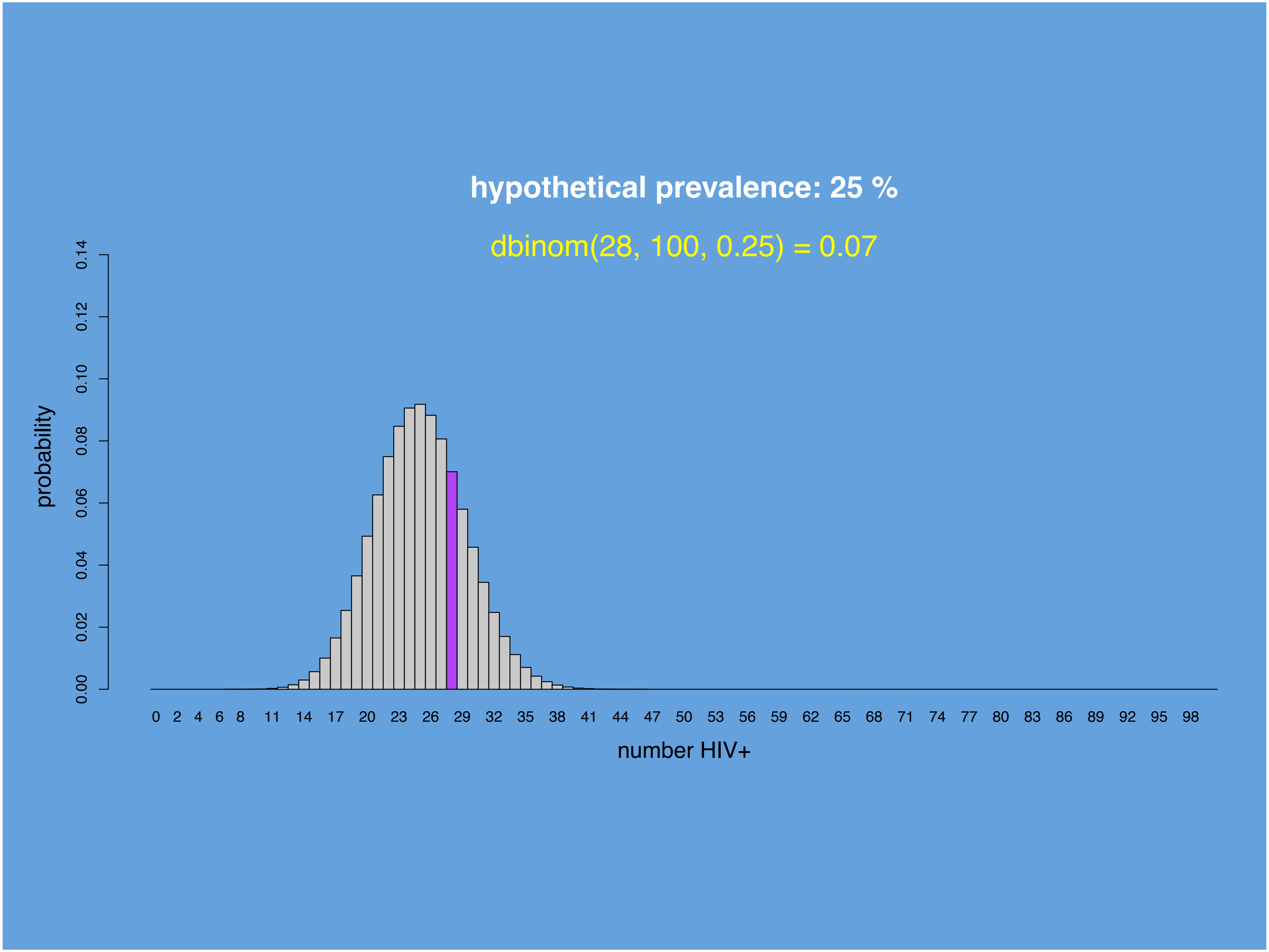
hypothetical prevalence: 25 %
dbinom(28, 100, 0.25) = 0.07
probability
0.00
0.02
0.04
0.06
0.08
0.10
0.12
0.14
0 2 4 6 8 11 14 17 20 23 26 29 32 35 38 41 44 47 50 53 56 59 62 65 68 71 74 77 80 83 86 89 92 95 98
number HIV+

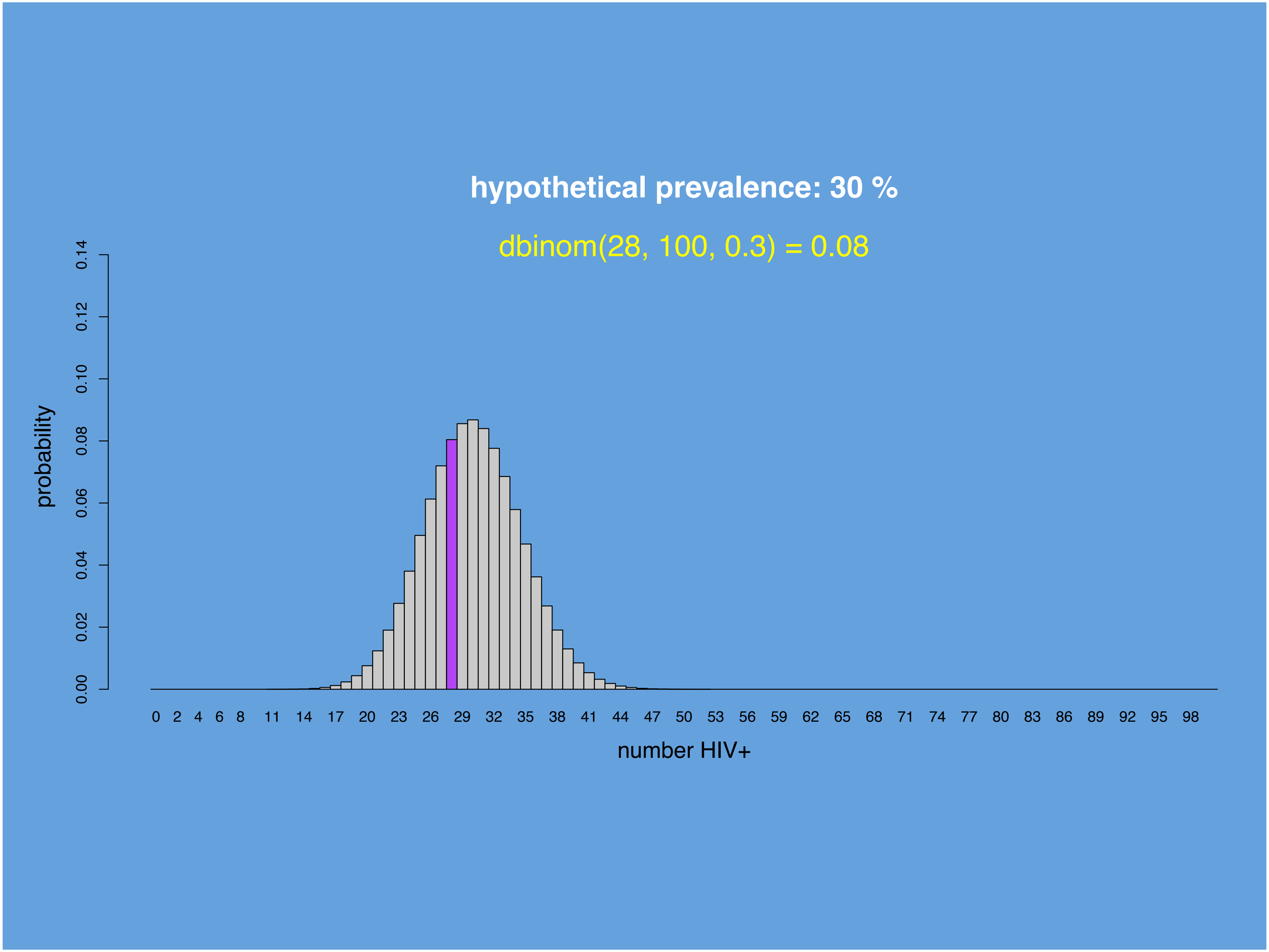

hypothetical prevalence: 30 %
dbinom(28, 100, 0.3) = 0.08
probability
0.00
0.02
0.04
0.06
0.08
0.10
0.12
0.14
0 2 4 6 8 11 14 17 20 23 26 29 32 35 38 41 44 47 50 53 56 59 62 65 68 71 74 77 80 83 86 89 92 95 98
number HIV+

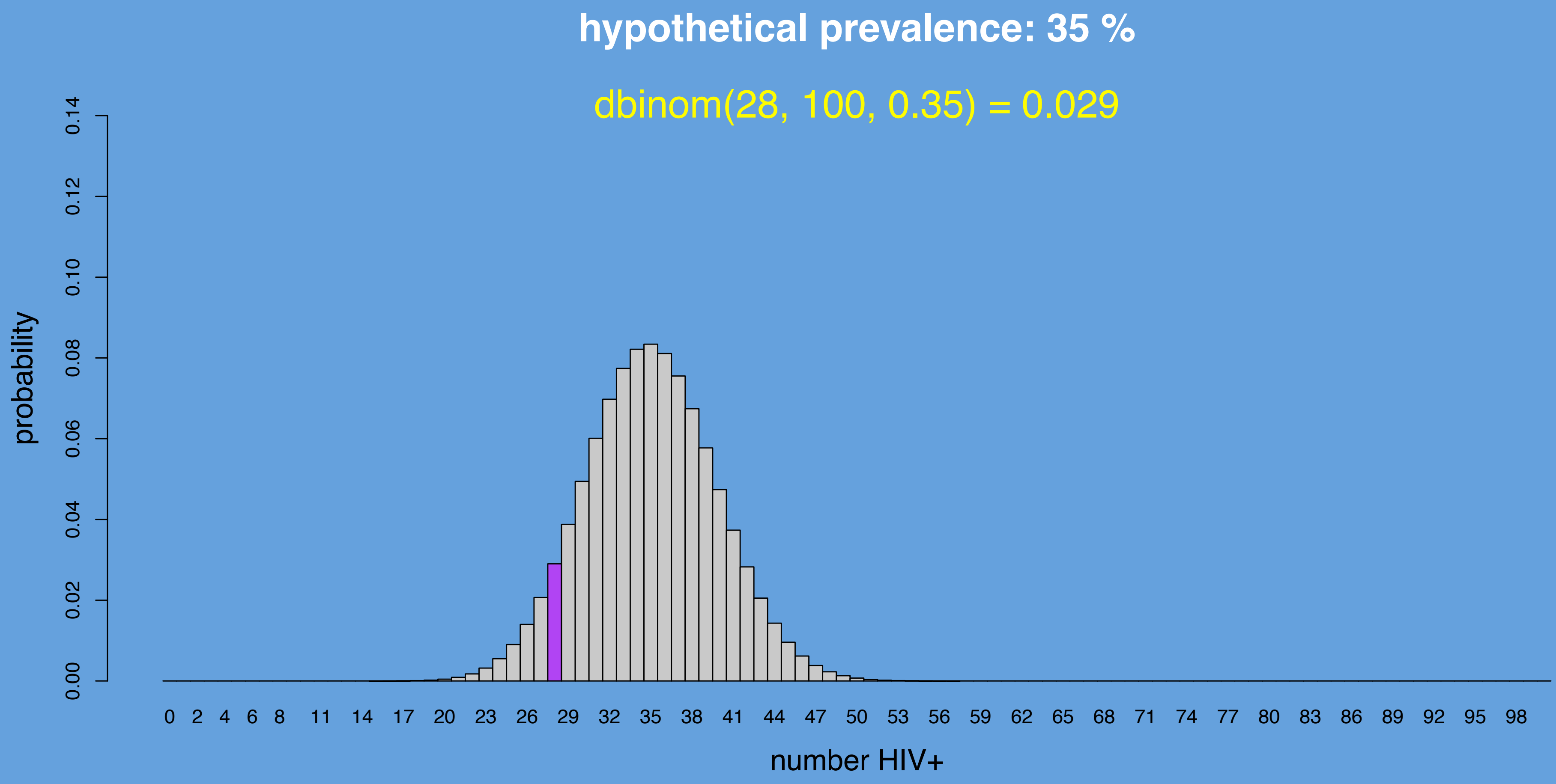

hypothetical prevalence: 35 %
dbinom(28, 100, 0.35) = 0.029
probability
0.00
0.02
0.04
0.06
0.08
0.10
0.12
0.14
0 2 4 6 8 11 14 17 20 23 26 29 32 35 38 41 44 47 50 53 56 59 62 65 68 71 74 77 80 83 86 89 92 95 98
number HIV+

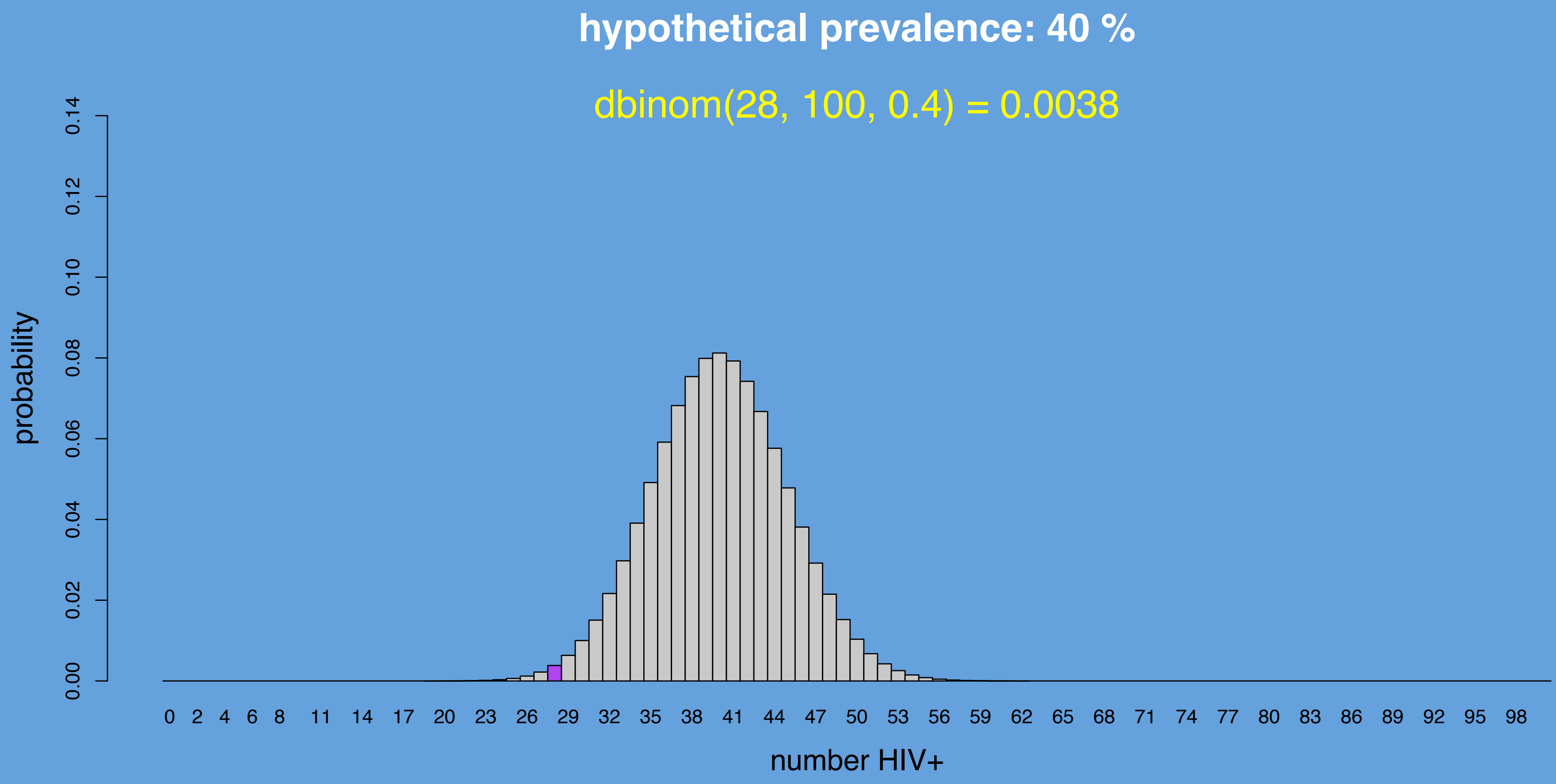

hypothetical prevalence: 40 %
dbinom(28, 100, 0.4) = 0.0038
probability
0.00
0.02
0.04
0.06
0.08
0.10
0.12
0.14
0 2 4 6 8 11 14 17 20 23 26 29 32 35 38 41 44 47 50 53 56 59 62 65 68 71 74 77 80 83 86 89 92 95 98
number HIV+

Which prevalence gives the greatest probability of observing exactly 28/100?

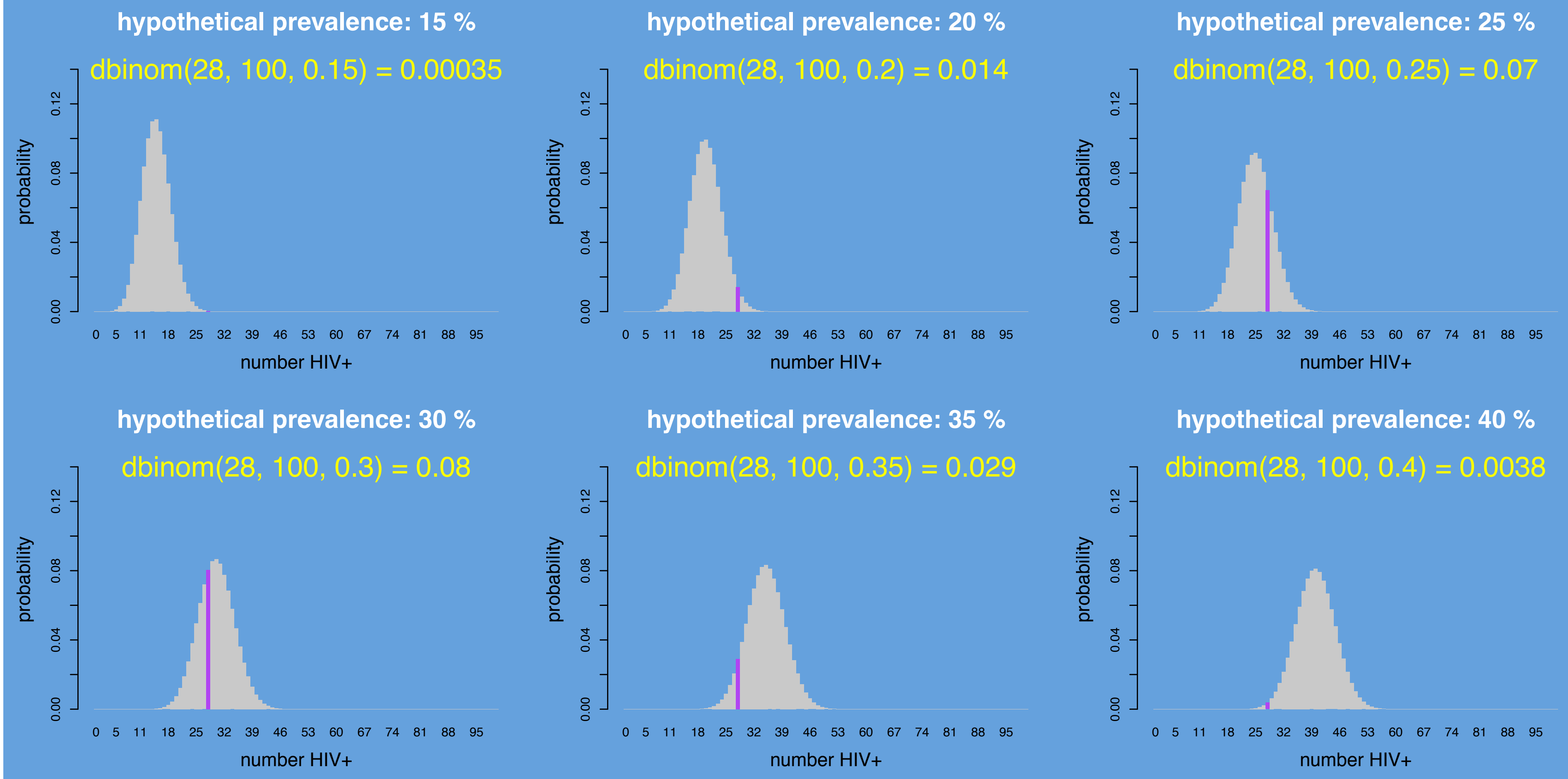

Which of these prevalence values is most likely given our data?

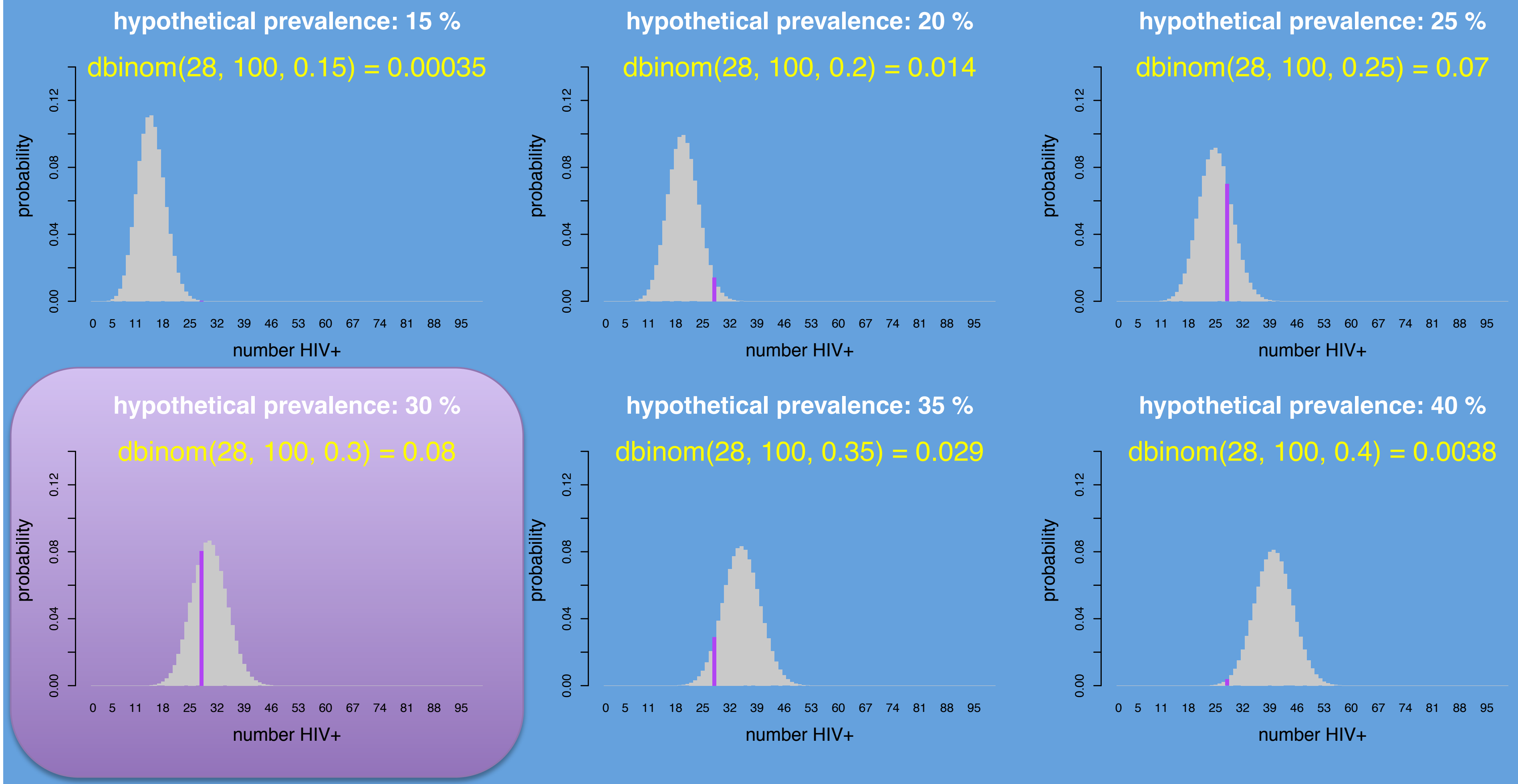

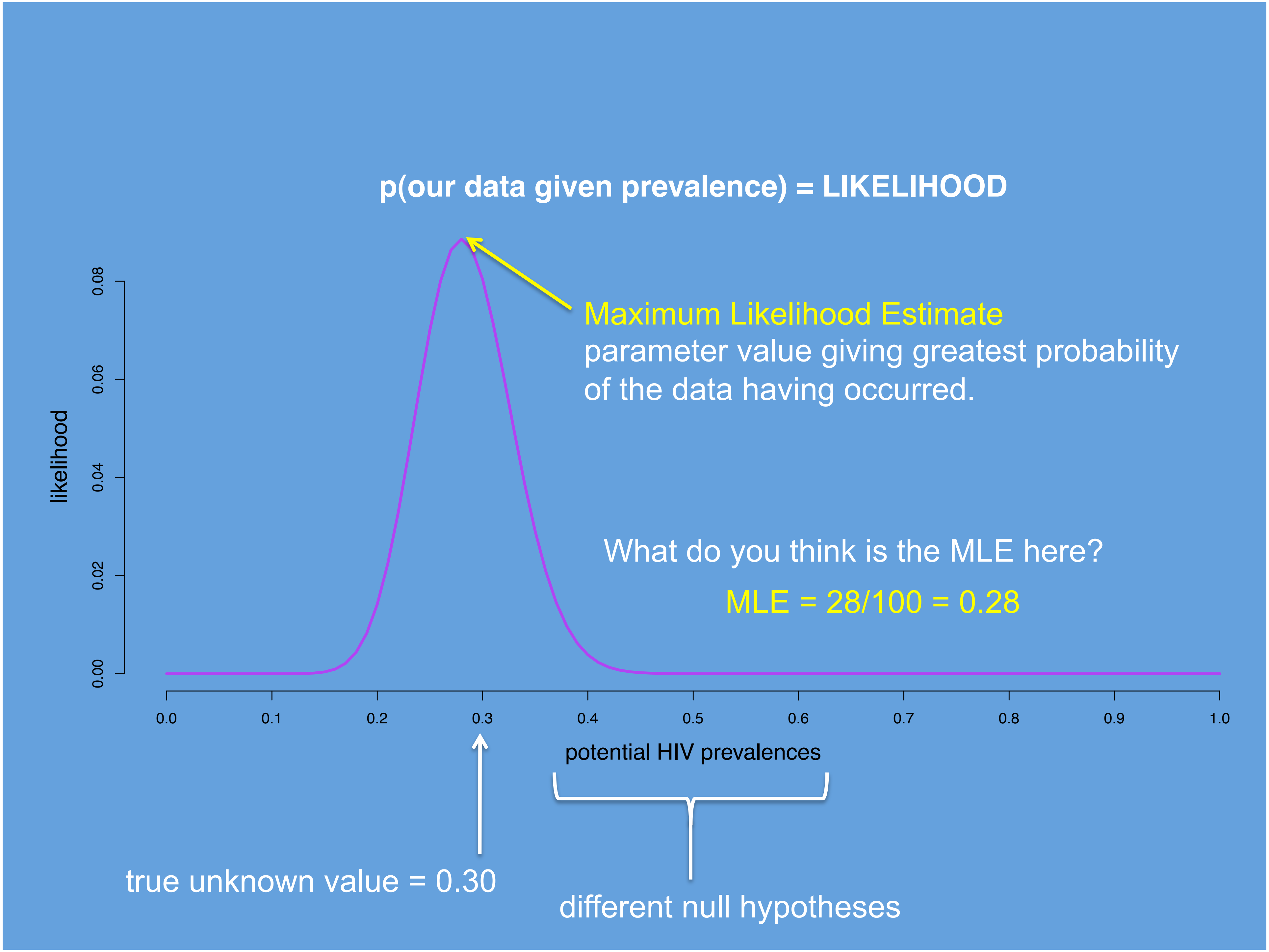
p(our data given prevalence) = LIKELIHOOD
Maximum Likelihood Estimate
parameter value giving greatest probability
of the data having occurred.
What do you think is the MLE here?
MLE = 28/100 = 0.28
likelihood
0.00
0.02
0.04
0.06
0.08
0.0
0.1
0.2
0.3
0.4
0.5
0.6
0.7
0.8
0.9
1.0
potential HIV prevalences
true unknown value = 0.30
different null hypotheses

Defining Likelihood

- L(parameter | data) = p(data | parameter)
- Not a probability distribution.
- Probabilities taken from many different distributions.

function of x

PDF: $f(x \mid p) = \binom{n}{x} p^x (1-p)^{n-x}$

LIKELIHOOD: $L(p \mid x) = \binom{n}{x} p^x (1-p)^{n-x}$

function of p

Deriving the Maximum Likelihood Estimate

maximize

$$L(p) = \binom{n}{x} p^x (1-p)^{n-x}$$

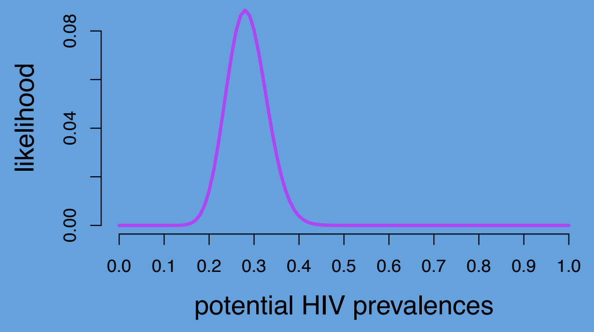

maximize

$$\log(L(p)) = \log\left[\binom{n}{x} p^x (1-p)^{n-x}\right]$$

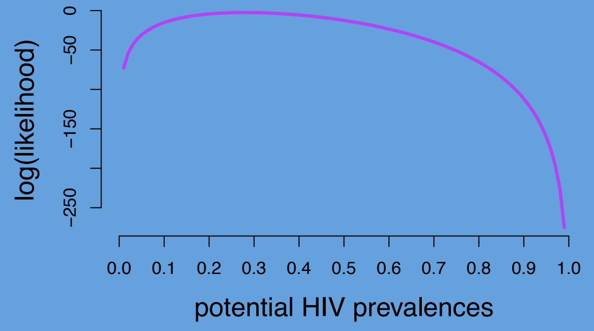

minimize

$$l(p) = -\log\left[\binom{n}{x} p^x (1-p)^{n-x}\right]$$

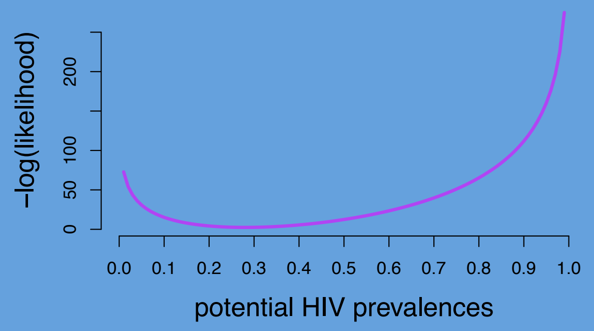

Deriving the Maximum Likelihood Estimate

$$l(p) = -\log(L(p)) = -\log\left[\binom{n}{x} p^x (1-p)^{n-x}\right]$$

$$l(p) = -\log\binom{n}{x} - \log(p^x) - \log((1-p)^{n-x})$$

$$l(p) = -\log\binom{n}{x} - x\log(p) - (n-x)\log(1-p)$$

Deriving the Maximum Likelihood Estimate

$$l(p) = -\log\binom{n}{x} - x\log(p) - (n-x)\log(1-p)$$

$$\frac{dl(p)}{dp} = \quad -\frac{x}{p} - \frac{-(n-x)}{1-p}$$

$$0 = -\frac{x}{\hat{p}} + \frac{n-x}{1-\hat{p}}$$

$$0 = \frac{-x(1-\hat{p}) + \hat{p}(n-x)}{\hat{p}(1-\hat{p})}$$

$$0 = -x + \cancel{\hat{p}x} + \hat{p}n - \cancel{\hat{p}x}$$

$$0 = -x + \hat{p}n$$

$$\hat{p} = \frac{x}{n}$$

The proportion of positives!

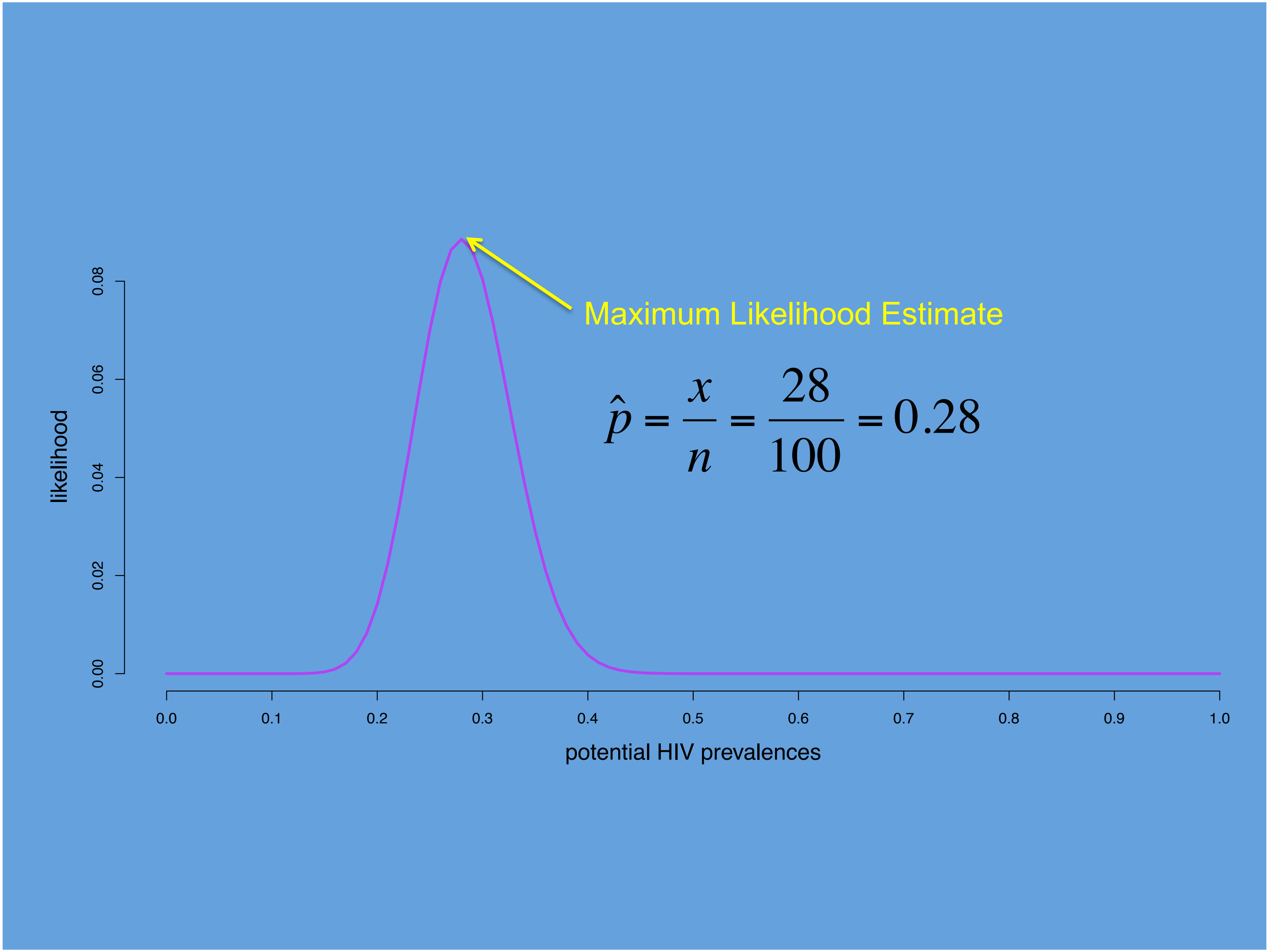

Maximum Likelihood Estimate
$\hat{p} = \frac{x}{n} = \frac{28}{100} = 0.28$
likelihood
0.00
0.02
0.04
0.06
0.08
0.0
0.1
0.2
0.3
0.4
0.5
0.6
0.7
0.8
0.9
1.0
potential HIV prevalences

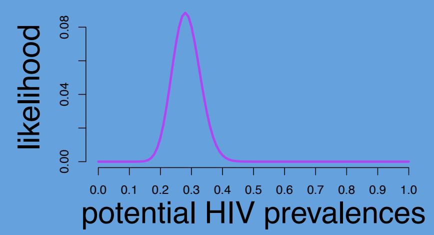

we usually minimize the –log(likelihood)

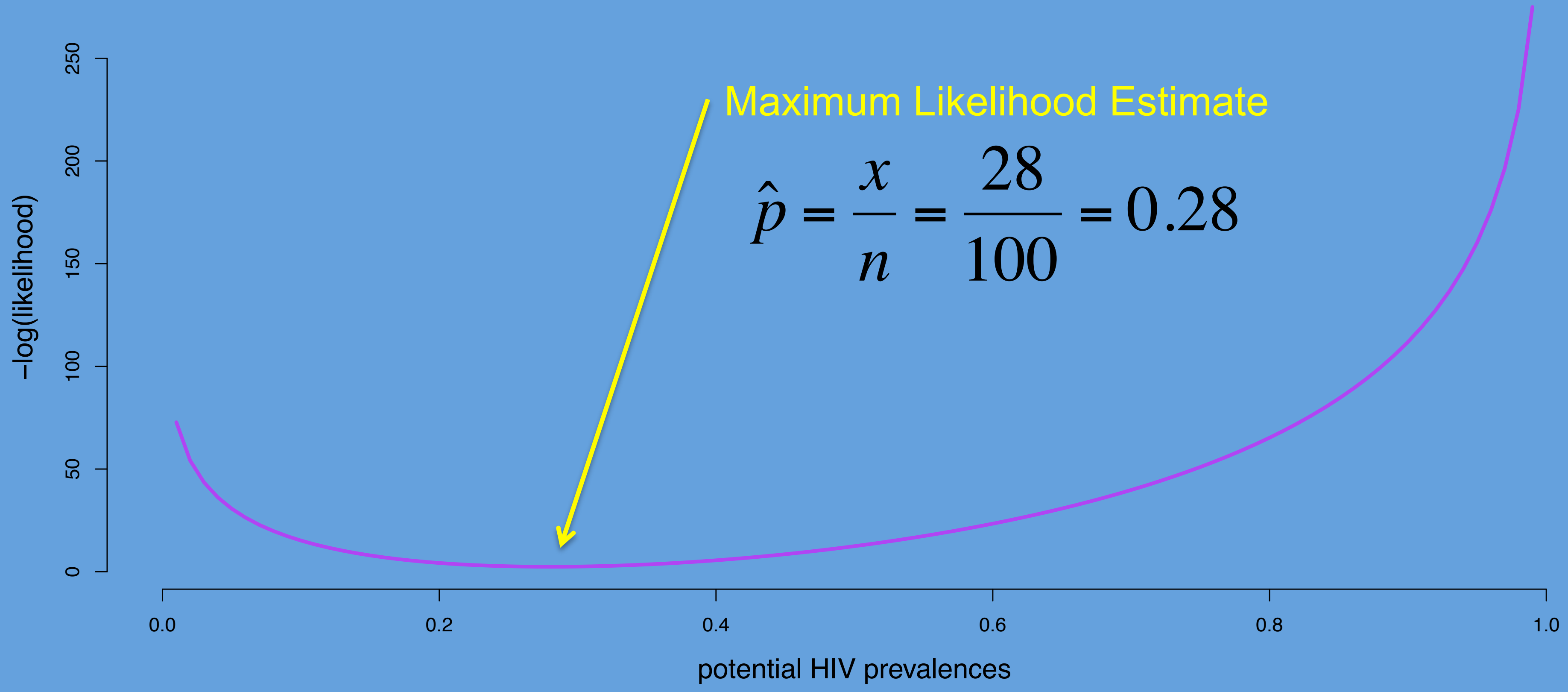

$$\hat{p} = \frac{x}{n} = \frac{28}{100} = 0.28$$

Building Confidence Intervals

Likelihood Ratio Test

If the null hypothesis were true then

$$-2\log\left(\frac{L(\text{null hypothesis})}{L(\text{alternative hypothesis})}\right) \sim \chi^2_{df=1}$$

$$2l_{alternative} - 2l_{null} \sim \chi^2_{df=1}$$

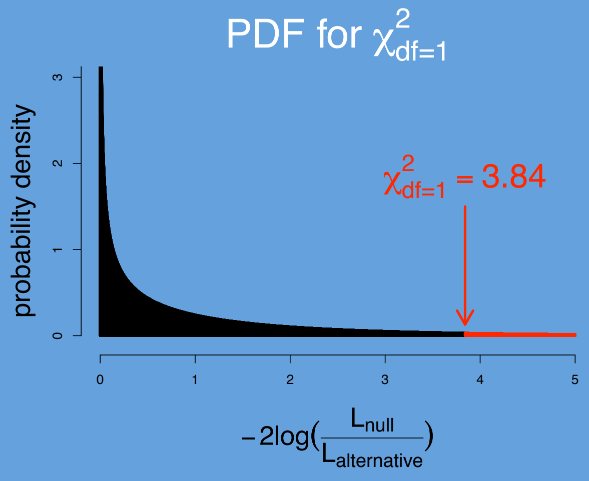

So if our α = .05, then we reject any null hypothesis for which

$$2l_{MLE} - 2l_{null} > \chi^2_{df=1,\ \alpha=0.05} = 3.84$$

```
> qchisq(p = .95, df = 1)
[1] 3.841459
```

$$2l_{MLE} - 2l_{null} > 3.84$$

$$l_{MLE} - l_{null} > 1.92$$

When $l_{MLE} - l_{null} > 1.92$,
we reject that null hypothesis.

Building Confidence Intervals

Likelihood Ratio Test

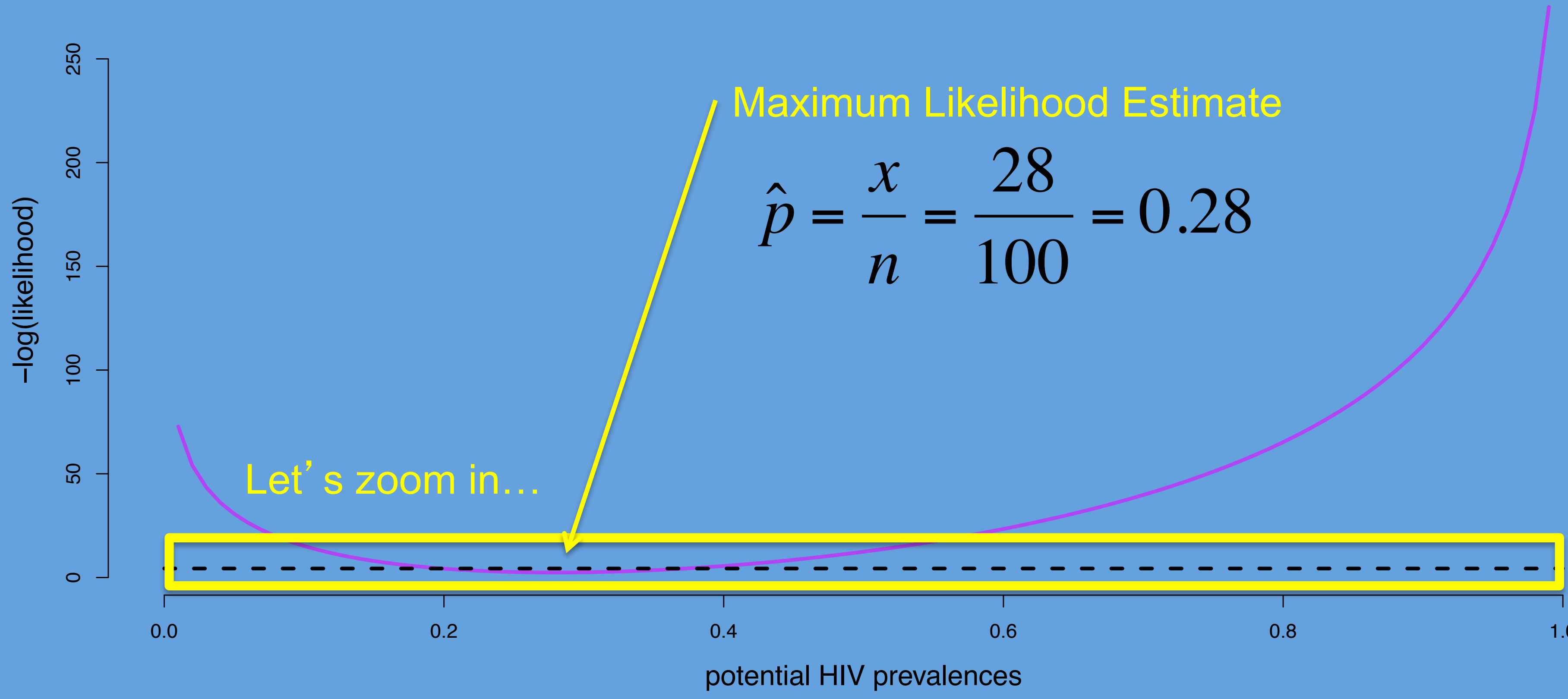

Building Confidence Intervals

Likelihood Ratio Test

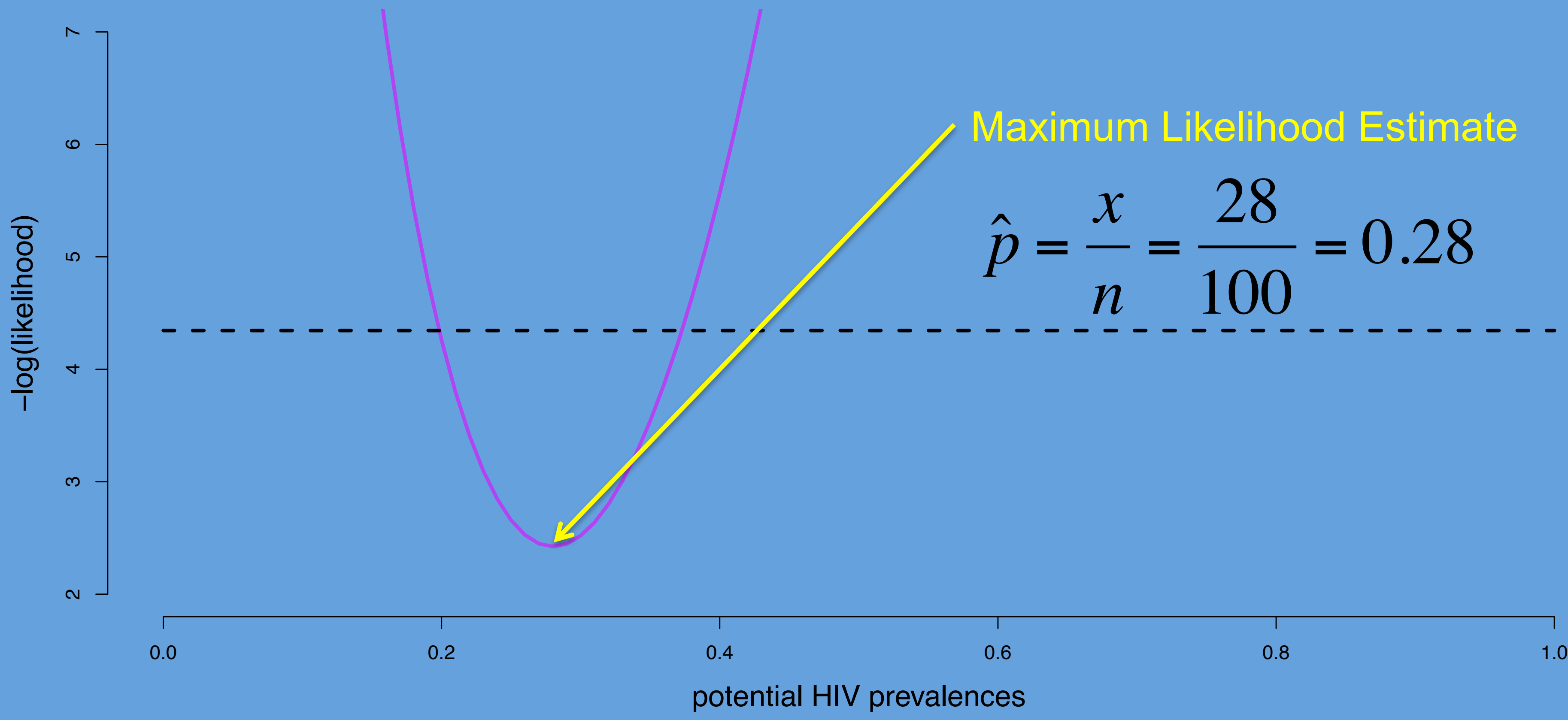

Building Confidence Intervals
Likelihood Ratio Test

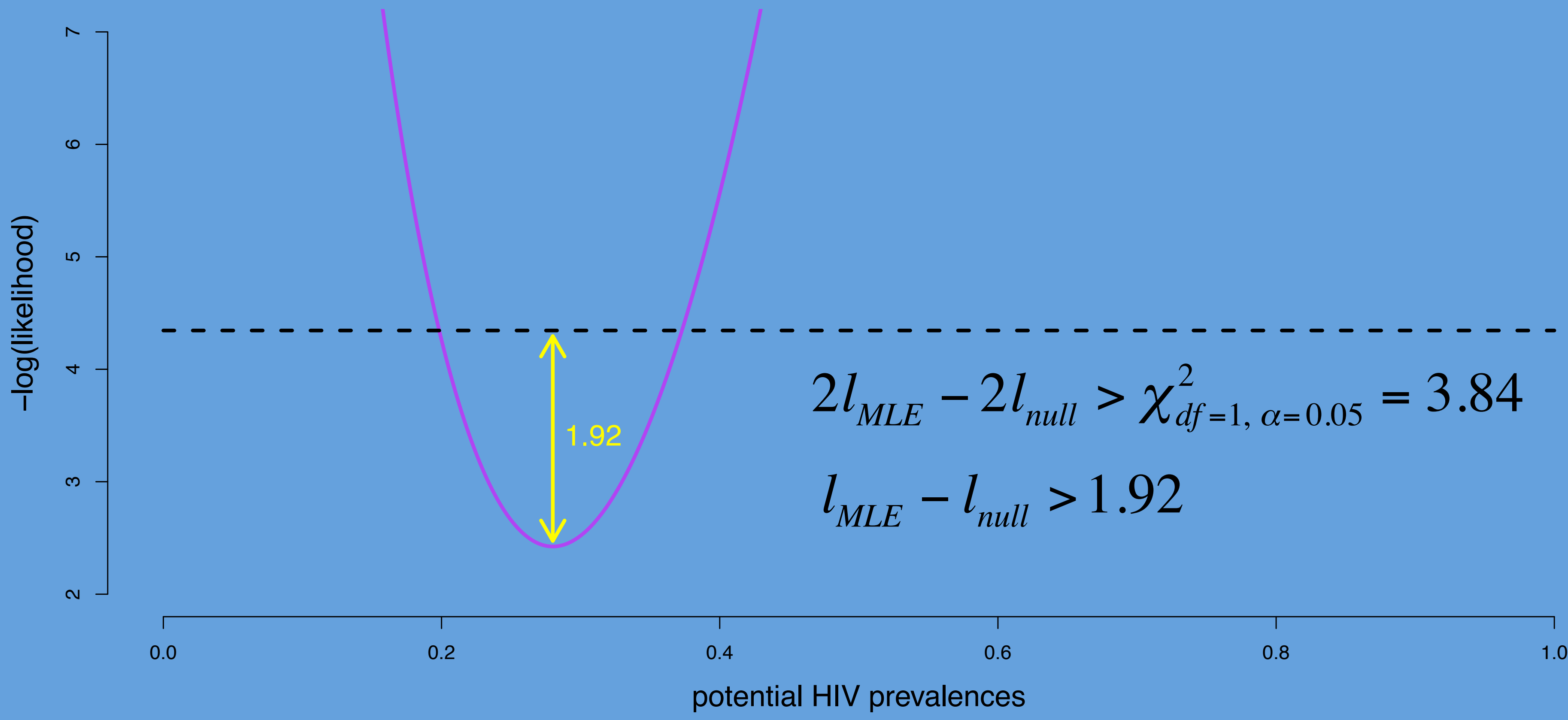

$$2l_{MLE} - 2l_{null} > \chi^2_{df=1,\ \alpha=0.05} = 3.84$$

$$l_{MLE} - l_{null} > 1.92$$

Building Confidence Intervals

Likelihood Ratio Test

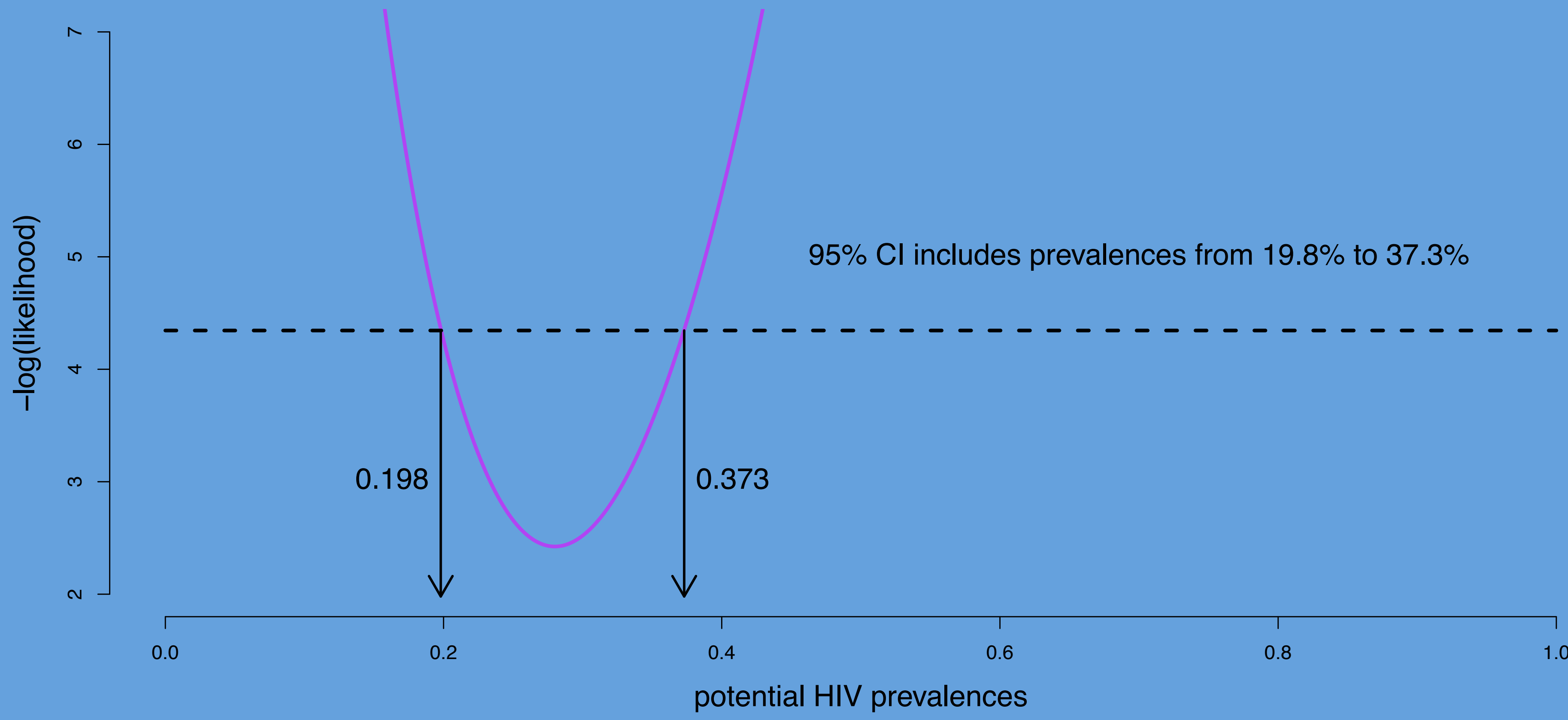

Comparing Confidence Intervals

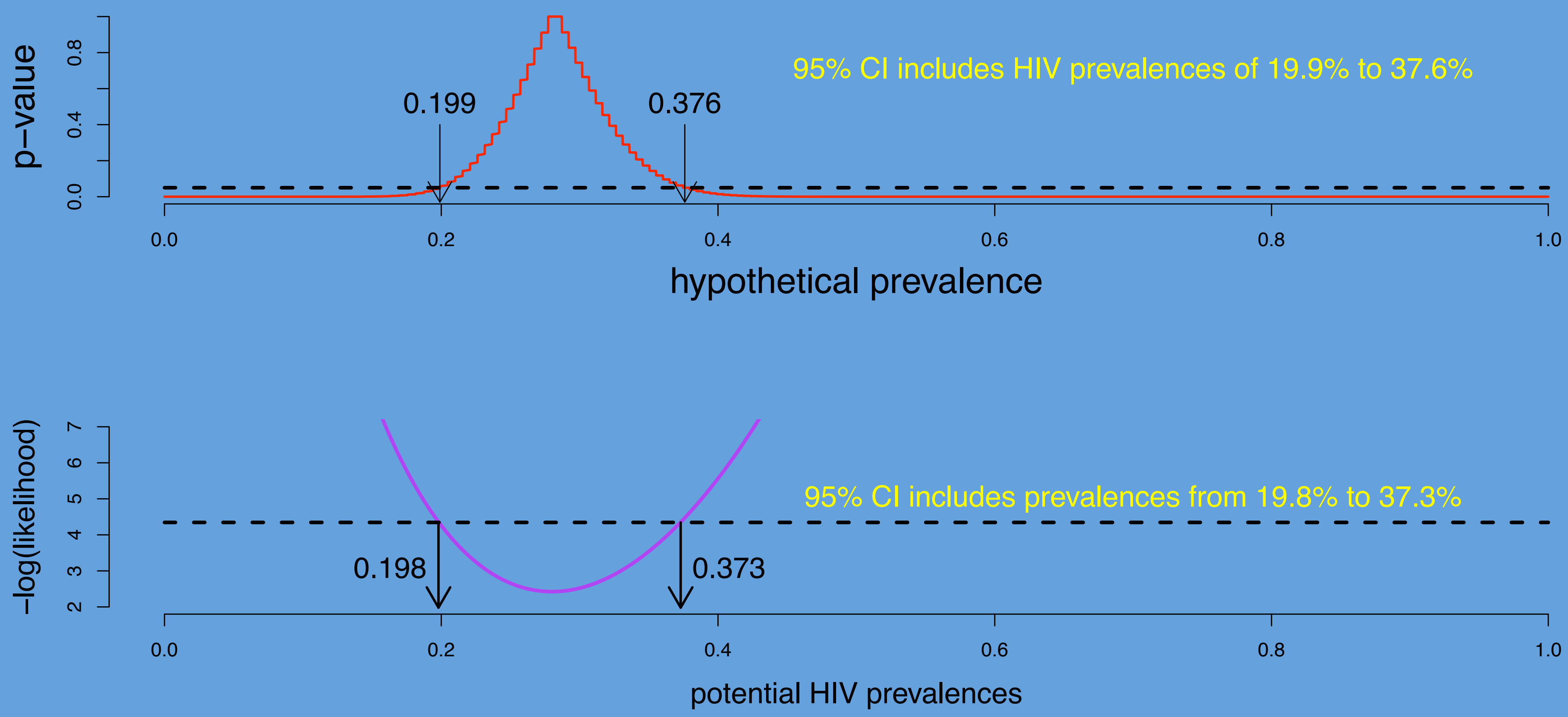

Advantages of Likelihood

- Practical method for
  - estimating parameters
  - estimating variance of our estimates

- Easily adaptable to different probability distributions & dynamic models
