# Supplementary material for: How to Make Epidemiological Training Infectious
Source: PLoS Biol. 2012 Apr 3;10(4):e1001295. doi: 10.1371/journal.pbio.1001295 (PMC3317897; doi:10.1371/journal.pbio.1001295)
Supplement: Figure S7 — Lecture slide 7: likelihood fitting and dynamic models I. (PDF) [file pbio.1001295.s014.pdf]

Fitting Mathematical Models to Data

Adapting Likelihood Based Inference

Meaningful Modeling of Epidemiologic Data, 2010
AIMS, Muizenberg, South Africa

Steve Bellan
MPH Epidemiology
Department of Environmental Science, Policy & Management
University of California at Berkeley

Fitting Dynamic Models to Data

Adapt our dynamic models in a probabilistic framework so we can ask:

What is the probability that a model would have generated the observed data?

What is the likelihood of a model given the data?

Likelihood of parameters
(given data)

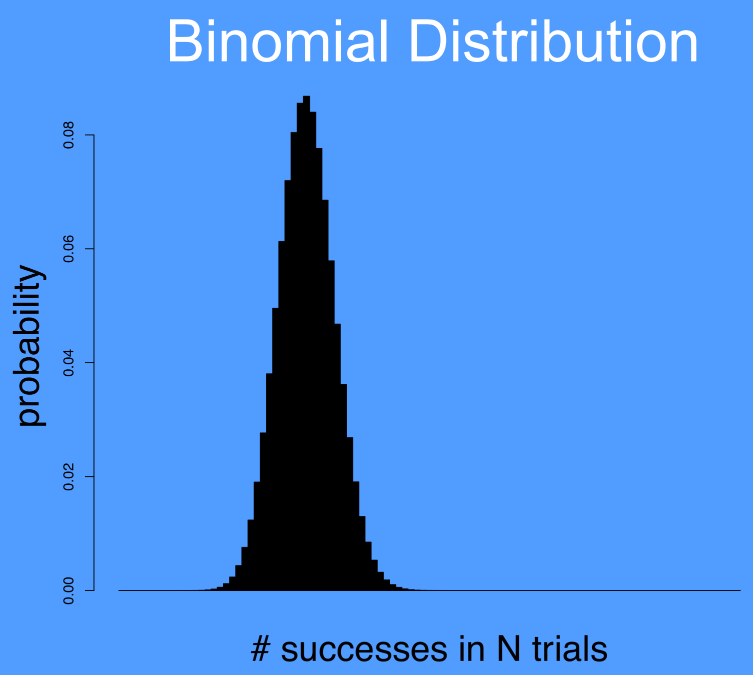

Distribution

↓

Likelihood of parameters (given data)

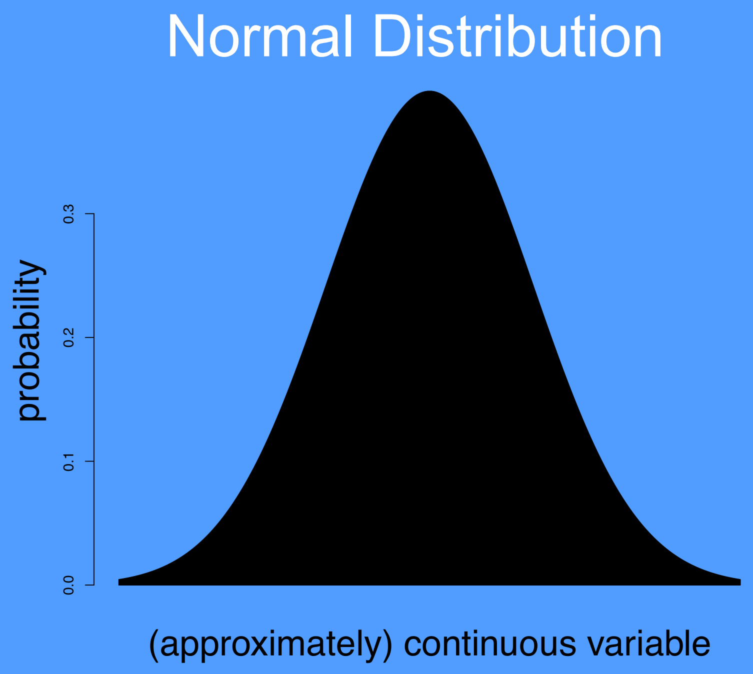

Distribution

↓

Likelihood of parameters
(given data)

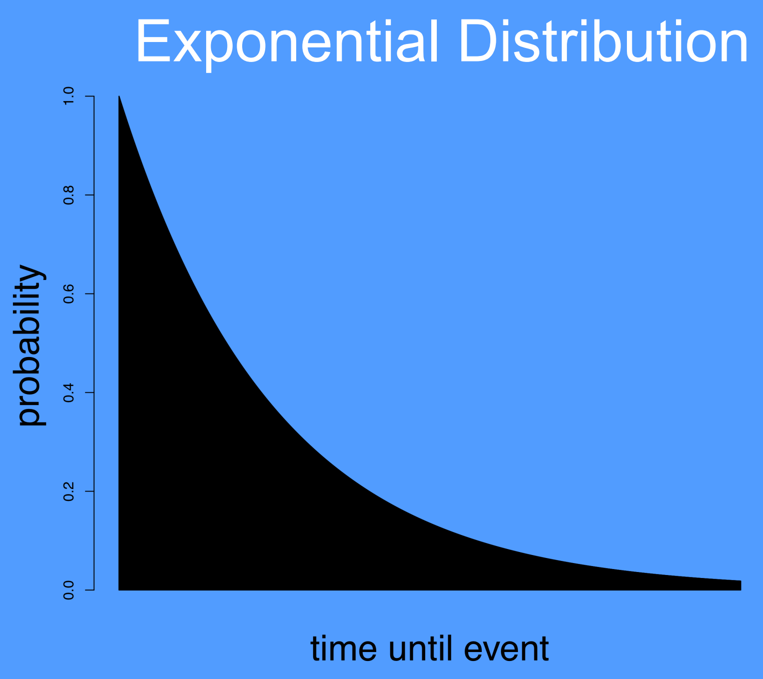

<u>Distribution</u>

<u>Likelihood</u> of parameters
(given data)

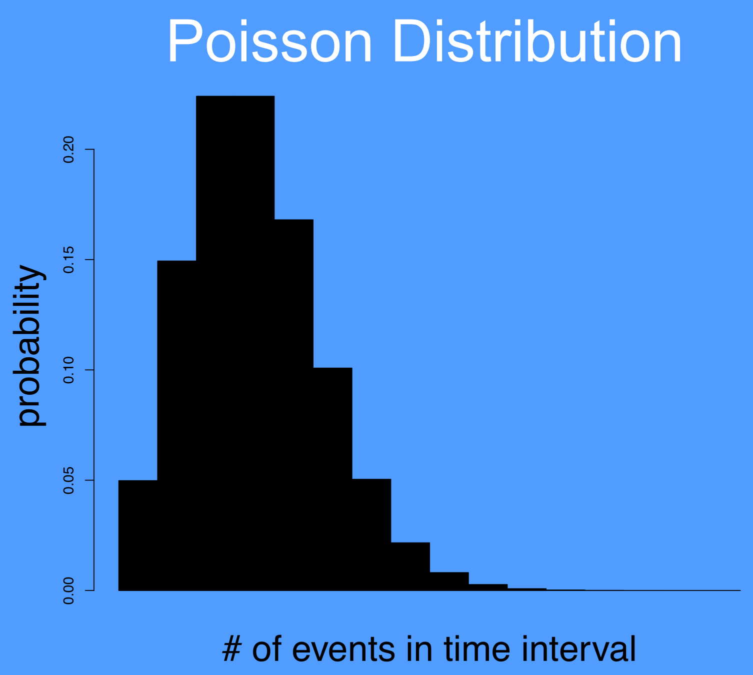

Distribution

Likelihood of parameters
(given data)

Stochastic Component of Model

Binomial Distribution

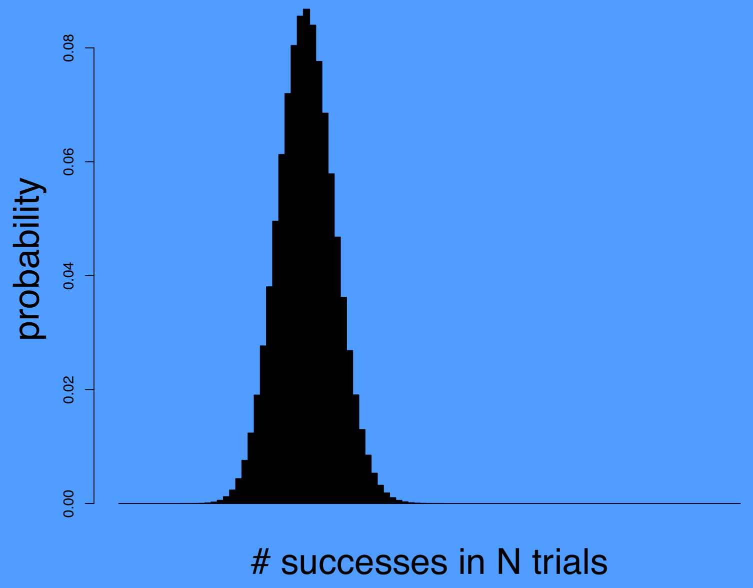

Distribution

Likelihood of parameters
(given data)

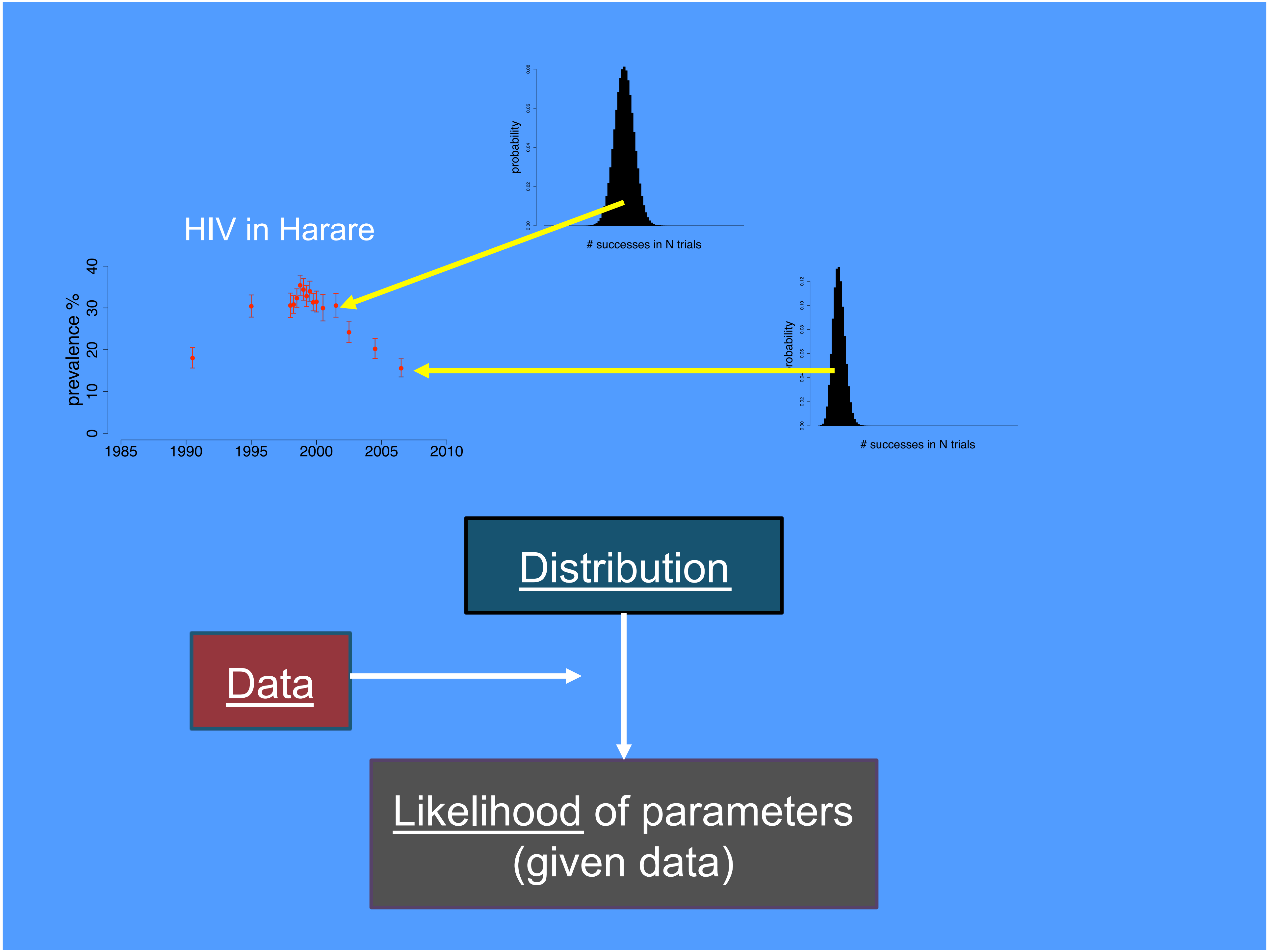

HIV in Harare
prevalence %
0
10
20
30
40
1985
1990
1995
2000
2005
2010
probability
successes in N trials
probability
successes in N trials
Distribution
Data
Likelihood of parameters
(given data)

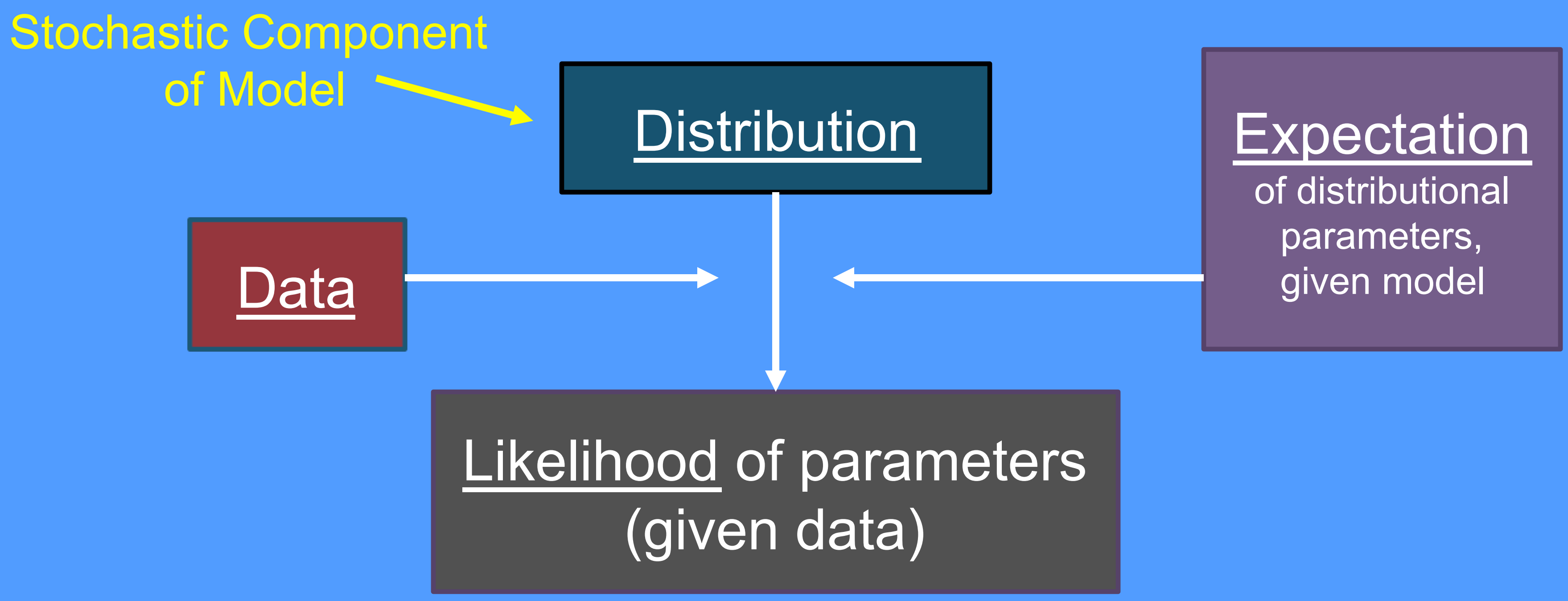
Stochastic Component
of Model
Distribution
Data
Expectation
of distributional
parameters,
given model
Likelihood of parameters
(given data)

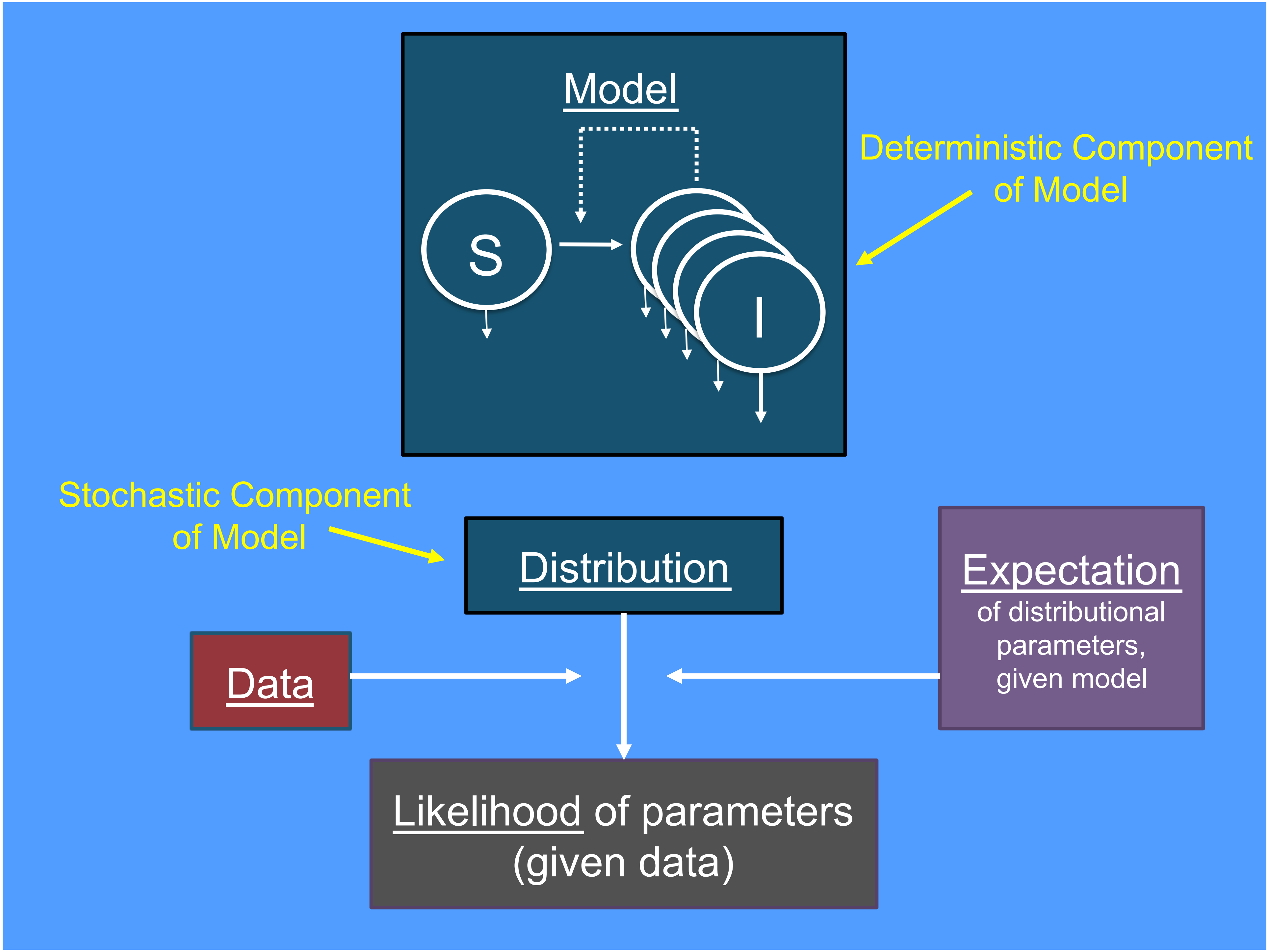
Model
S
I
Deterministic Component of Model
Stochastic Component of Model
Distribution
Data
Expectation
of distributional parameters, given model
Likelihood of parameters (given data)

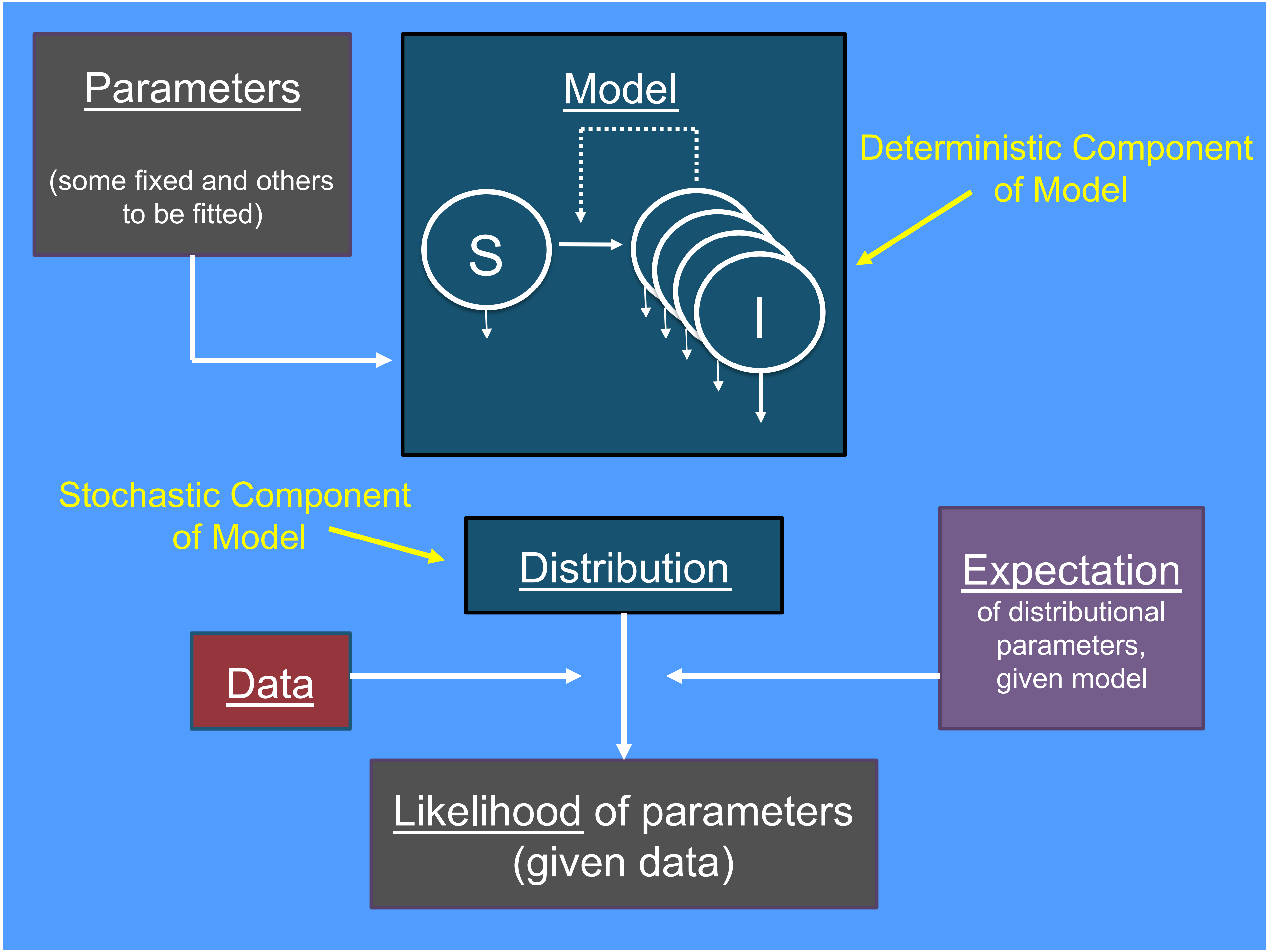
Parameters
(some fixed and others to be fitted)
Model
S
I
Deterministic Component of Model
Stochastic Component of Model
Distribution
Expectation
of distributional parameters, given model
Data
Likelihood of parameters (given data)

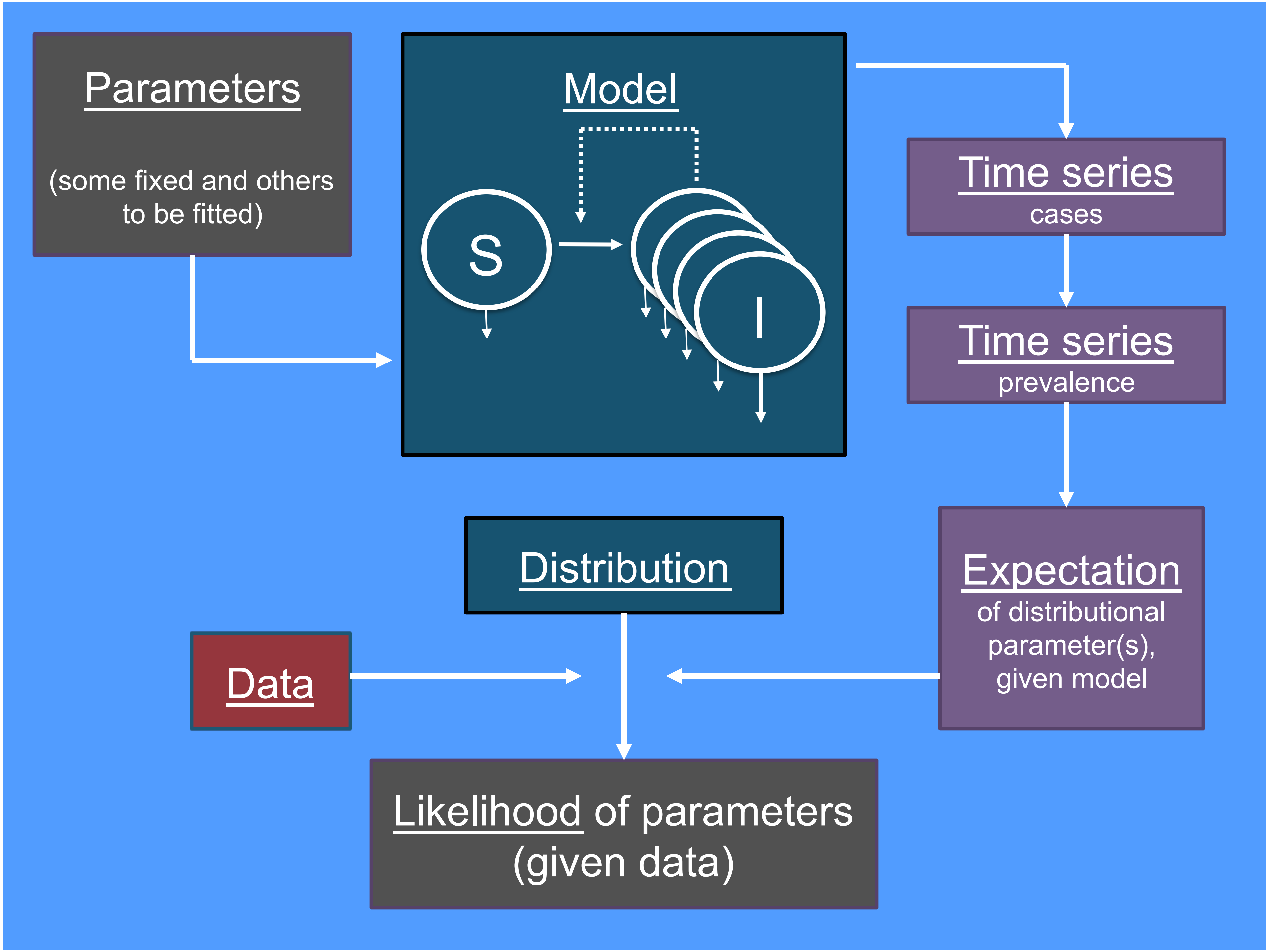
Parameters
(some fixed and others to be fitted)
Model
S
I
Time series
cases
Time series
prevalence
Distribution
Expectation
of distributional parameter(s), given model
Data
Likelihood of parameters (given data)
