# Supplementary material for: How to Make Epidemiological Training Infectious
Source: PLoS Biol. 2012 Apr 3;10(4):e1001295. doi: 10.1371/journal.pbio.1001295 (PMC3317897; doi:10.1371/journal.pbio.1001295)
Supplement: Text S1 — This file includes a glossary and detailed information for instructors on implementation of the exercise, including ideas for development of group projects and description of possible variations. (DOC) [file pbio.1001295.s016.doc]

**Text S1**

**Glossary of Terms**

***Classical epidemiology***:  Building on assumptions of independence between individuals (or clusters thereof), researchers identify causal relationships between risk (or protective) factors and disease . Once having determined a causal relationship between a risk factor and a disease , public health policy can be adjusted accordingly either by, for example, targeting high risk groups, widely implementing protective factors (*i.e.* vaccination), or counseling behavioral changes. This approach is reductionist, attempting to disentangle the effect of each risk factor on disease outcomes without allowing for non-independence due to dynamics of an infectious disease. Classical epidemiology is necessarily rooted in careful study design to avoid sources of bias and is also closely allied with methodological developments in biostatistics that allow sophisticated analyses of diverse data sets. A mainstay of classical epidemiology is the randomized controlled trial. Randomization of interventions to study participants minimizes the chance that a confounding variable is the cause of a spurious relationship, increasing the chance that any observed relationship is indeed causal. However, even when causal relationships are established their underlying biological mechanisms may not be understood. Classical epidemiology blossomed with the rise of chronic disease epidemiology and increasingly sophisticated extensions to classical analyses have been developed to estimate population level effects, account for time-varying confounders, and deal with random variation and dependence at multiple scales .

***Risk Factors***: Classical epidemiology is largely concerned with identifying factors that are associated with an increased probability of disease.  The goal of analytical studies usually is to estimate the relative risk of a given health outcome with a particular risk factor versus without that risk factor. Factors that are associated with a decreased probability of disease are sometimes called protective factors though the term risk factor is loosely used as the name for any variable potentially related to a health outcome.  These include behavioral, genetic, environmental, physiological variables as well as public health interventions (*i.e.* vaccination).

***Stochasticity (Observation Error)***: Classical epidemiology is primarily concerned with random error due to observation error.  Observation error can be due to sampling variability or measurement error.  Sampling variability is error in an estimate due to the fact that only a sample of the population was measured.  Measurement error is due to imperfect measurement of variables.

***Bias*:** Bias (systematic error) is any difference between the observed value and the true value due to any cause other than random error.  Empirical design in classical epidemiology traditionally delineates three types of bias. The first, selection bias, occurs when individuals selected to be in the study are chosen based on a variable that is associated with both the exposure and outcome of interest. The second, information bias, occurs when risk factors or outcomes are systematically misclassified. The third type of bias, confounding, occurs when a third variable is associated with both the risk factor and outcome of interest inducing a spurious (and non-causal) association between them if this third variable is not controlled for in study design or analysis.

***Dynamical Epidemiology***: Dynamical (or mechanistic) epidemiology focuses on modeling the mechanistic processes governing transmission in order to understand how individual-level interactions lead to population level patterns.  This focus on interactions makes dynamical epidemiologist a systems approach, in contrast to the reductionist approach of classical epidemiology. Nonlinear mathematics is used to describe the processes of interest and systems are either solved analytically or by simulation to answer questions of interest. When model parameters are poorly understood, sensitivity analyses—in which model results are assessed over a wide parameter range—can be used to yield more conservative conclusions. Models can also be fit to data directly to epidemiological time series data to estimate unknown parameters and to assess hypotheses governing transmission dynamics. Questions answered can either be theoretical or very applied. Dynamical epidemiology is particularly useful for answering questions that cannot be answered empirically due to ethical or logistical reasons.

***R_0_***: The basic reproduction number, or *R_0_*, is defined as the average number of secondary cases that would be infected by an index infectious individual introduced into a susceptible population. The effective reproduction number, *R_eff_*, is the same quantity but in a population that is not necessarily entirely susceptible. These are fundamental concepts in dynamic epidemiology because it allows characterization of the relative contagiousness of various pathogens as well as the ease with which they can be regulated.  Epidemics cannot occur when *R_eff_* < 1, since this means that on average infectious individuals will create < 1 new infection before they recover and the number of infected individuals will decrease over time. Importantly, *R_0_* is directly related to the proportion of the population that must be immune to cause an epidemic to wane.  For instance if *R_0_* = 4, then 1-1/4 = 0.75 is the proportion of the population that must be immune to eliminate the disease. In that case, of the 4 people normally infected by each individual, 3 would be immune and infected individuals would only replace themselves by one other infected individual, meaning the number of infected individuals could not increase.

***Force of infection*:** Dynamic approaches construct individual-level risk from not only of individual characteristics and risk factors, but also the “force of infection” in the population - *i.e.*, the density of infectious agents.  So, for example, malaria risk depends not only on whether an individual drains standing water and installs window screens, but also on whether the mosquitoes in the area are malarious.

***Depletion of susceptibles*:** At the population level, whether a disease spreads depends not only on intrinsic characteristics of the disease and the population, but on what proportion of the population is currently either infected, or “removed” (rendered immune, taken out of circulation, or killed) by the action of the disease.

***Stochasticity--Process Error*:** While also concerned with observation error as in classical epidemiology, dynamical epidemiology tends to place much greater focus on process error.  Process error or randomness is due to the fact that though any give process has a distribution of outcomes, each event can only take one such value.  For example, *R_0_* = 4 this is the expected number of infections produced by an index case.  However, such an index case my by chance only infect 0 individuals before recovering, meaning an epidemic will never even start. Process error plays a large role when a few individuals have a disproportionate importance in dynamics for instance at the beginning or end of an epidemic.

**Concept and Simulation**

Infectious disease transmission is a complex, nonlinear dynamical phenomenon. Risk factor epidemiology aims to understand what variables put individuals, clusters of individuals, or populations at higher or lower risk of disease by regressing health outcomes against risk (or protective) factors and other potentially confounding covariates . Mathematical epidemiology explicitly analyzes infectious disease epidemics in a nonlinear dynamical framework and focuses on the population level phenomena that emerge from individual level processes . Such studies can also be used towards the same goals as risk factor epidemiology (risk factor identification), for instance in identifying the protective effectiveness of vaccines, vector control, and sanitation measures. Mathematical models can also, however, help us understand mechanisms that are difficult to assess using the methods of risk factor epidemiology, such as the demographic drivers of epidemics (susceptible burnout and replenishment) and the causes of periodicity and seasonality. Furthermore, mathematical models provide a platform to conduct sensitivity analyses to determine the most effective interventions amongst many, particularly when large-scale studies are expensive and logistically difficult or ethically unfeasible.

Stochastic simulation of epidemics with computers is a valuable tool for research purposes and can also be used in training. However, we propose that stochastic real life simulation as a pedagogical alternative that exposes less mathematically inclined students to epidemiological dynamics in a fun and engaging way and exposes mathematicians to the effects of real-world processes that are essential to understand when building data-driven models. The following section outlines our protocol for propagating an epidemic in course participants, though we imagine that this exercise could be done in a variety of other ways. The number of participants and their level of background should determine in large part the complexity of the exercise.

Rather than giving an exact explication of the exercise as run in either year, the below description of the simulation and group exercises is a generic outline of the exercise that incorporates elements of MMF outbreaks and their analysis as conducted at MMED 2010 and 2011.

**The Infectious Agent**

The infectious agent in our exercise is a sheet of paper (Supplementary File S1) that gives the recipient step-by-step instructions in how to transmit the agent to other individuals and how to report to the organizers of the exercise (*i.e.* analogous to the DNA or RNA of biological pathogens). This paper was made available on the MMED website for download, with the link provided in the instructions.­­ While we relied on participants’ access to the internet and a double-sided printer for easy transmission, the exercise organizers could keep a stack of infectious agents ready to give to newly infected individuals. The steps of the transmission and reporting processes as given in the infectious agent itself are described in detail below:

1. A newly infected individual who just received the pathogen should immediately report via email (or other viable alternative) to one of the exercise organizers. This allows unrealistically accurate tracking of the epidemic for comparison with passive symptomatic surveillance.
2. Next, the newly infected individual will use a random number generator to determine how many people s/he will have infectious contacts with. We use a Poisson distribution with rate parameter (*i*.*e*. mean) of 2.5. This is equivalent to *R*_0_, the average number of cases infected per case in an otherwise susceptible population . We chose *R_0_* by running stochastic simulations of epidemics that took into account the number of participants such that an outbreak sufficiently large for the below analyses would occur. Using the command

   > rpois(n = 1, lambda = 2.5)

   R will produce the number of infectious contacts. Random number generation can also be done in most statistical software packages, Excel, or through online applets as well (please feel free to contact us if you have questions). We stress that this step should only be performed *once*. If individuals keep generating numbers until they get a sufficiently large number or small number then the initial secondary case distribution will no longer be Poisson distributed around 2.5. As transmission continues, some of these infectious contacts will not be successful because some individuals will already be infected or recovered.
3. The infected individual will then print (or otherwise acquire from an exercise organizer) the appropriate number of infectious agent papers as determined in step 2.
4. Next, the infected individual will hand each infectious agent (sheet of paper) to a participant in the exercise. *Discretion is a key part of this step*; without it individuals may avoid infected individuals that they believe have already been infected and this would defeat the purpose of the exercise. It also makes the exercise more fun and suspenseful as participants individually become clued in to what is occurring without knowing who else knows. Participants are limited to the closed population of the exercise (in our case, MMED students and instructors). We chose to limit the time period between which individuals became infected and infected others (i.e., the combined latent and infectious period) to 48 hours as MMED only spans 2 weeks.
5. To simulate the underreporting of most diseases, we had infected individuals determine whether they were symptomatic or asymptomatic with a Bernoulli number generator in R:

   > rbinom(n = 1, size = 1, p = 0.8)

   If R returns a 1 individuals are symptomatic, otherwise they are asymptomatic. Symptomatic individuals report to one of the exercise organizers (analogous to a clinician) that they are infected.
6. The infected individual then fills out a table recording who they exposed and when. This provides an unrealistically accurate contact tracing data set. Time of infection also helps with the estimation of latent periods.
7. The infected individual then is instructed to return the infectious agent (sheet of paper) to one of the exercise organizers to prevent other participants from seeing it or transmitting it. This step also allows collection and centralized management of essential data that cannot be collected for real-life epidemics (such as the timing of infectious events and the actual number of infectious events that did not produce cases due to immunity (or, in some cases, nonparticipation)).
8. The last and not least important step discusses immunity. Individuals can only be infected *once*. If they are handed a second form they do not repeat steps 1-6 but are instructed to hand the form into an exercise organizer. Without this step the realistic susceptible depletion that causes epidemics to burn out would not occur.

**Variations of the Exercise**

One of the virtues of this exercise is that it can flexibly incorporate many variations that can simulate the dynamics of biological infectious agents. While innumerable complexities are possible we caution against developing too complex an exercise in a small group as the complexities will be difficult to tease apart in the analyses. The following are some examples of variations:

1. Circulate multiple pathogen strains that differ in infectiousness (*R*_0_).
2. Circulate multiple pathogen strains that differ in the symptomatic proportion.
3. Circulate multiple pathogen strains that differ in the duration of the infectious period.
4. Increase (or decrease) heterogeneity in the secondary case distribution by using a negative binomial distribution instead of a Poisson distribution .
5. Allow for food-borne transmission by distributing the infectious agent papers discreetly with certain food items and then allow secondary person-to-person transmission to propagate.
6. Allow for disease treatment such that all (or a proportion) of individuals reporting as symptomatic to the “physician” are cured and cannot further infect individuals (*i*.*e*. the “physician” takes their sheets away).
7. Allow for population-wide control measures, such as unavailability of the website or printer for generating new infectious agents.
8. Use exponential or gamma distributed waiting times to determine the latent period (*i.e*. time between infection of an individual and when s/he can infect others).
9. Use exponential or gamma distributed waiting times to determine the infectious period (*i.e*. time between when the latent period ends and an individual recovers (must have made all infectious contacts)).

**Group Work**

Our students conducted a number of studies on MMF by analyzing data collected during the outbreak and collecting new data to investigate hypotheses of MMF spread. Participants were divided into groups of 4-6 students, each paired with 1 or 2 mentors. Each group focused on a specific set of questions and the methodologies appropriate to answer them. On the final day of the workshop, each group presented their methods and findings to the whole group, which ensured that all participants had some exposure to each approach. Below we briefly describe each group project. Commented R code for each project is provided in Supporting Information file Text S3.

**Risk Factor Epidemiology Analyses**

***Survey design***. Students conducting case-control, cohort, and contact tracing studies designed two questionnaires to collect data on potential risk factors for MMF infection, including information on demographic characteristics and contacts prior to and during the workshop. The groups were provided with references on questionnaire design and given brief instruction in writing closed-ended questions and consideration of variable types for data collection (e.g., continuous numeric values, mutually exclusive categories, and true/false options). Groups designing questionnaires initially met to discuss the types of information they were interested in gathering and received input from the mentors on the feasibility of data collection on specific topics of interest. The participants in each group then drafted an initial questionnaire, modified it according to feedback from mentors and each other, discussed instructions for asking each question, and tested the modified version by administering the questionnaire to each other and the group mentors. The final questionnaires (e.g., Text S5) were produced by making necessary modifications after testing and re-testing among group participants. The group members then divided the MMED participant list equally and interviewed all available participants (e.g., in 2010, 46 of 50 people on the list). Each group member was interviewed by one of the other group members rather than administering the questionnaires to themselves.

***Data entry, management, and cleaning***. Data collected during the outbreak (including infection and recovery times, information on reported infections, etc) were entered and cleaned by the mentors, and these data were provided to the appropriate groups as CSV files. Questionnaire data were entered and cleaned by the groups conducting survey design. These data were entered into spreadsheets using Google Documents to allow simultaneous data entry by multiple group members. Due to time constraints data could not double-entered, so group members spot-checked data entered by other group members to identify potential inconsistencies in data entry. Data were then exported to CSV files and imported into R for further cleaning and analysis. Group members were given example code showing how to ensure that all variables were in an appropriate format for analysis (i.e., appropriately defined as factors, numerical values, binary vectors, character vectors, date/time vectors, etc) and that all values of each variable fell within a reasonable range or defined category.

***Case-control and retrospective cohort.*** Students reviewed basic observational study designs and measures of effect (*i.e.* odds ratios, risk ratios). Subsequently, participants utilized questionnaire data to identify potential risk factors associated with MMF infection. Analyses focused on examining 2x2 tables and calculating crude odds ratios along with 95% confidence intervals. Additional statistical techniques including Fisher’s Exact Test, Chi-square tests, Wald tests, and Logistic Regression with multiple explanatory variables were also considered (Figure 2). For the latter, students incorporated subject matter knowledge to examine the presence of both confounding and interaction in multiple regression models. Because MMED clinic participants represented the only susceptible population (i.e. only clinic participants could become infected with MMF), and time at risk could be reasonably estimated based on participants’ arrival at the clinic, students also calculated estimates of relative risk including both cumulative incidence ratios and incidence density ratios. Poisson regression models could then be constructed to identify risk factors for MMF after controlling for potentially confounding variables.

**Mathematical Epidemiology Analyses**

The following analyses are summarized in Table 1.

***Visual inspection of the epidemic time series*.** Groups visualized the epidemic by converting infection and recovery events into a time series and then plotting the number of susceptible, infected, and recovered/immune individuals over time (e.g., Figure 1).

***Estimation of R*_0_.** The basic reproductive number (*R*_0_, defined as the expected number of infections caused by a single infected individual in an otherwise susceptible population) was estimated directly from the contact tracing data available from the MMF infection forms. The effective reproductive number (*R_effective_*, defined as the expected number of infections caused by a single infected individual in a partially immune population) was then plotted over time by multiplying *R*_0_ times the proportion of the population that was susceptible.

***Estimation of incubation/latent & infectious periods.*** We estimated the infectious period for this exercise, but serial interval (distribution of time between successive infection) and number of transmission events could be estimated in the same way. Students conducted maximum likelihood fits to reported time intervals by coding in R. They chose to model infectious periods as being gamma distributed as the gamma distribution is a simple, commonly used distribution with a reasonable amount of flexibility. They also discussed the limitations to fitting data of this sort. The main obstacle identified to simple model fits was the large importance of time of day, given both the fast time scales of the epidemic, and the nature of transmission.

***Stochastic simulation of outbreaks.*** Groups used the gillespieSSA package in R or wrote their own Gillespie algorithms to model what sorts of outbreaks would be expected given what was known about disease parameters. They constructed a simple, event-based simulator for an SEIR infection, and did two sets of simulations. The first set of 1000 simulations used maximum-likelihood estimates to explore the range of different outcomes expected under fixed parameters. The second used parameters sampled at random using the likelihood distributions calculated above. These reflect what sort of variation would be expected from another realization of a similar outbreak, given the amount of information we have from the observed outbreak. Using the best estimates from the 2011 MMF outbreak, 1000 outbreaks were simulated at a variety of levels of population immunity to show how the distribution of outbreak size would be expected to vary with different levels of pre-existing immunity (either from previous outbreaks or vaccination; Figure 2B).

**Presentation of Results**

We suggest that each project group should present their analyses (in 10-20 minute talks) to the full group of participants, as this motivates participants to focus on the goal and conclusions of their analyses rather than the methodological tool itself. At the MMED clinics, presentations also provided an opportunity for groups to learn about many different methods—particularly the challenges dealt with and conclusions made from each analysis— since each group only had time to learn and implement one or two types of analyses each.

**Take Home Messages**

After all the presentations have been given is a good time for the organizers (or volunteering participants) to summarize the goal and utility of each method presented, providing a clear take home message on how mathematical and risk factor tools in epidemiology differ but complement each other. This is also an appropriate time to stress that these tools need not remain distinct and that mathematical models can be used to test hypotheses regarding risk factors, but that this becomes difficult when very little is known about the causal relationship between a risk factor and the disease outcome of interest. We also believe this is a good point to provide information on how participants can learn more about these methods (*i.e.* links to websites, names of books, relevant journal articles). As many of MMED participants are from African institutions with little access to resources, we particularly stress open access journals and books. For a list of some of these resources, see:

<http://lalashan.mcmaster.ca/theobio/mmed/index.php/Resources>

**Cited References**

1. Galea S, Riddle M, Kaplan GA (2009) Causal thinking and complex system approaches in epidemiology. International Journal of Epidemiology 39: 97-106.

2. Koopman JS, Lynch JW (1999) Individual causal models and population system models in epidemiology. Am J Public Health 89: 1170-1174.

3. Susser M, Susser E (1996) Choosing a future for epidemiology: II. From black box to Chinese boxes and eco-epidemiology. Am J Public Health 86: 674-677.

4. Rothman KJ, Greenland S (2005) Causation and causal inference in epidemiology. Am J Public Health 95 Suppl 1: S144-150.

5. Susser M, Susser E (1996) Choosing a future for epidemiology: I. Eras and paradigms. Am J Public Health 86: 668-673.

6. Grassly NC, Fraser C (2008) Mathematical models of infectious disease transmission. Nat Rev Micro 6: 477-487.

7. Hethcote HW (2000) The mathematics of infectious diseases. Siam Review 42: 599-653.

8. Lloyd-Smith JO, Schreiber SJ, Kopp PE, Getz WM (2005) Superspreading and the effect of individual variation on disease emergence. Nature 438: 355-359.

9. Gillespie DT (1977) Exact stochastic simulation of coupled chemical reactions. The Journal of Physical Chemistry 81: 2340-2361.
