# Supplementary material for: How to Make Epidemiological Training Infectious
Source: PLoS Biol. 2012 Apr 3;10(4):e1001295. doi: 10.1371/journal.pbio.1001295 (PMC3317897; doi:10.1371/journal.pbio.1001295)
Supplement: Text S2 — The infection notification form used as the infectious agent. Before use, the form should be tailored to the specific course or classroom setting by adjusting the parts of the form highlighted in yellow. Instructors may also want to change the name of the “disease” to fit the context of their course. (DOC) [file pbio.1001295.s017.doc]

You have been

INFECTED

with Muizenberg Mathematic Fever (MMF)!!!

Please complete the following steps:

1. **Send an email to Instructor** ([user@domain.net](mailto:user@domain.net)) with the subject:

“I’VE GOT THE FEVER!”

In the body of the message, include the **date and time** you received this piece of paper.

1. **Figure out how many people you will infect** using the following code in the programming language R. R is on all the AIMS computers and can be accessed by typing R in the terminal window (you’ll all know how to do this by the second day):

   rpois(n = 1, lambda = 2.5)

   ONLY DO THIS ONCE! The number R returns is the number of people you will infect.

**Write down the value R returned:**

1. **Go to the following webpage** and print out the number of infection notifications you need to hand out (i.e., the number R returned in step 2):

<http://www.coursewebsite.com/location_of_this_sheet_for_download.pdf>

1. **Hand each infection notification to another course participant**. A few notes:
   1. ONLY hand the sheet to people listed on the below web site (you may hand the sheet to ANYONE listed on the website)
      <http://www.coursewebsite.com/participant_list.html>
   2. Hand out all of your infection notifications **within 48 hours** of having received your own infection notification.
   3. When you hand people an infection notification, be sure to fold it so other people cannot see what is written on it. Hand it to them quietly and when no one else is around so that other participants do not notice.
2. **Symptoms**: Type the following code into R:

   rbinom(n = 1, size = 1, p = .8)

ONLY ENTER THIS ONCE! **If R returns a 1, tell Another Instructor that you have MMF** as soon as possible. **If R returns a 0, do not tell anyone** that you have been infected.

**Write down the value R returned:**

1. In the table below, write down the names of the people you hand the infection notifications to and the date and time that you hand them the notification:

| **Name** | **Date** | **Time** |
| --- | --- | --- |
|  |  |  |
|  |  |  |
|  |  |  |
|  |  |  |
|  |  |  |
|  |  |  |

**Also write down your name:**

1. **Hand in THIS PIECE OF PAPER to An Instructor** after giving out all of your infection notifications**.**
2. **IMMUNITY**: MMF confers lifetime immunity. You can only be infected once! If someone hands you another infection notification, take it and don’t say anything. Do NOT repeat steps 1-6, but do hand the paper to Instructor in Step 7.

***If you need help with any of the steps, do not ask anyone for help except Specified Instructors!!***
