# Supplementary material for: How to Make Epidemiological Training Infectious
Source: PLoS Biol. 2012 Apr 3;10(4):e1001295. doi: 10.1371/journal.pbio.1001295 (PMC3317897; doi:10.1371/journal.pbio.1001295)
Supplement: Text S5 — Survey developed by MMED participants following the 2011 MMF outbreak to gather data on potential risk factors. (PDF) [file pbio.1001295.s020.pdf]

MMF SURVEY

NAME:___________________________________

Demographic Questions

1. How old are you? _______

2. Gender? M F

3. Level of study? Honours Masters Ph.D. Post-doc Professor Other:__________

4. When did you arrive? Before Monday Monday Tuesday After Tues.
   a. What was their first day they participated in MMED? __________

MMF Questions

1. Did you show symptoms of MMF? Y N
   a. If yes, when? MON TUES WED THURS FR

2. Were you at MMED last year? Y N
   a. If yes, did you get MMF? Y N

Social Questions

1. Did you go to the Ice-breaking activities last week? Y N
   a. Were you there for the big knot? Y N
   b. Did you play cards afterwards? Y N

2. Did you go to drumming? Y N

3. Did you go to coffee/tea early last week (up to Wednesday)? 6 4-5 2-4 <2

4. Did you wear your name-tag early last week? Y N
   a. Did you stop wearing it? Y N
   b. If so, when did you stop? MO TU W TH FR

5. Where are you staying? AIMS WHALE WATCHERS
   a. Floor? ________
   b. Room-mate? Y_____________ N
